# Treating patients with driving phobia by virtual reality exposure therapy – a pilot study

**DOI:** 10.1371/journal.pone.0226937

**Published:** 2020-01-07

**Authors:** Y. Kaussner, A. M. Kuraszkiewicz, S. Schoch, Petra Markel, S. Hoffmann, R. Baur-Streubel, R. Kenntner-Mabiala, P. Pauli

**Affiliations:** 1 Department of General Practice, University of Würzburg, Würzburg, Germany; 2 Department of Psychology 1 (Biological Psychology, Clinical Psychology, and Psychotherapy), University of Würzburg, Würzburg, Germany; 3 Würzburg Institute for Traffic Sciences (WIVW), Veitshöchheim, Germany; 4 Center of Mental Health, Medical Faculty, University of Würzburg, Würzburg, Germany; Brown University, UNITED STATES

## Abstract

**Objectives:**

Virtual reality exposure therapy (VRET) is a promising treatment for patients with fear of driving. The present pilot study is the first one focusing on behavioral effects of VRET on patients with fear of driving as measured by a post-treatment driving test in real traffic.

**Methods:**

The therapy followed a standardized manual including psychotherapeutic and medical examination, two preparative psychotherapy sessions, five virtual reality exposure sessions, a final behavioral avoidance test (BAT) in real traffic, a closing session, and two follow-up phone assessments after six and twelve weeks. VRE was conducted in a driving simulator with a fully equipped mockup. The exposure scenarios were individually tailored to the patients’ anxiety hierarchy. A total of 14 patients were treated. Parameters on the verbal, behavioral and physiological level were assessed.

**Results:**

The treatment was helpful to overcome driving fear and avoidance. In the final BAT, all patients mastered driving tasks they had avoided before, 71% showed an adequate driving behavior as assessed by the driving instructor, and 93% could maintain their treatment success until the second follow-up phone call. Further analyses suggest that treatment reduces avoidance behavior as well as symptoms of posttraumatic stress disorder as measured by standardized questionnaires (Avoidance and Fusion Questionnaire: p < .10, PTSD Symptom Scale–Self Report: p < .05).

**Conclusions:**

VRET in driving simulation is very promising to treat driving fear. Further research with randomized controlled trials is needed to verify efficacy. Moreover, simulators with lower configuration stages should be tested for a broad availability in psychotherapy.

## Introduction

There are about 5.770.000 police-reported traffic crashes in the U.S. each year [1]. Rates of PTSD and other psychopathologies like acute stress reactions, adjustment disorders or specific (isolated) phobias following a traffic accident range from 8 to over 30% [2–4]. These disorders often go along with fear of driving, rumination about traffic catastrophes and worries about suffering from or causing an accident again. In result, these patients avoid driving in general; avoid specific driving situations, like highways or unknown routes [5–7], or cope with the fear-inducing situations by overcautious driving.

According to several guidelines, exposure-based interventions are recommended as the treatment of choice for anxiety disorders [8–10] including PTSD and specific phobias [11–14]. Virtual reality exposure therapy (VRET) works just like in vivo or in sensu (imaginal) exposure, with one difference: the fear-inducing stimuli or situations are simulated by a computer. Nevertheless, the patients are active participants in this method [15, 16]. The advantages of VRET in comparison to in vivo exposure were described repeatedly (e.g. [3, 17–20]): Importantly, refusal rates are lower (3% for VRET versus 27% for in vivo exposure) and—if a direct choice is offered—acceptance is higher (76% preference of VRET versus in-vivo exposure) [21]. Furthermore, VRET is characterized by high situational and therapeutic control as the relevant exposure situations can be selectively designed and purposefully repeated or prolonged so that they fit the patients’ individual anxiety hierarchy perfectly without unexpected interferences.

Treatment efficacy of VRE has been proven for PTSD (e.g. [17]) and various specific phobias and panic disorder (e.g. [15, 22–24]). However, studies examining the efficacy of VRET to treat fear of driving are rare [24]. Beck et al. [19] treated six patients with PTSD after a motor vehicle accident with eight one-hour sessions of therapist supported exposure in a motion-based driving simulator. Post-treatment assessment revealed treatment success as indicated by less re-experience of anxiety, avoidance and emotional numbing. Wald [25, 26] treated five patients with fear of driving that completed eight weekly graded VRET sessions. Driving phobia and avoidance declined in three subjects, the remaining two showed no significant changes. Walshe, Lewis, Kim, O’Sullivan and Wiederhold [27] examined the effectiveness of twelve graded VRE sessions on seven patients with driving phobia after an accident. Post-treatment results indicated significant reduction in reported distress, driving anxiety and travel avoidance. Additionally, a decline in posttraumatic stress symptoms, depression symptoms and heart rate during exposure was reported.

Despite the quite small study samples, these studies indicate very promising effects. However, VRE effects on real driving behavior as assessed by a behavior avoidance test (BAT) have not been evaluated yet. The present pilot study is the first one focusing on effects of VRET on patients with driving fear as measured by a post-treatment BAT in real traffic. Maintenance of treatment success was assessed by six and twelve week follow-up interviews.

## Methods

### Participants

**Fig 1:**
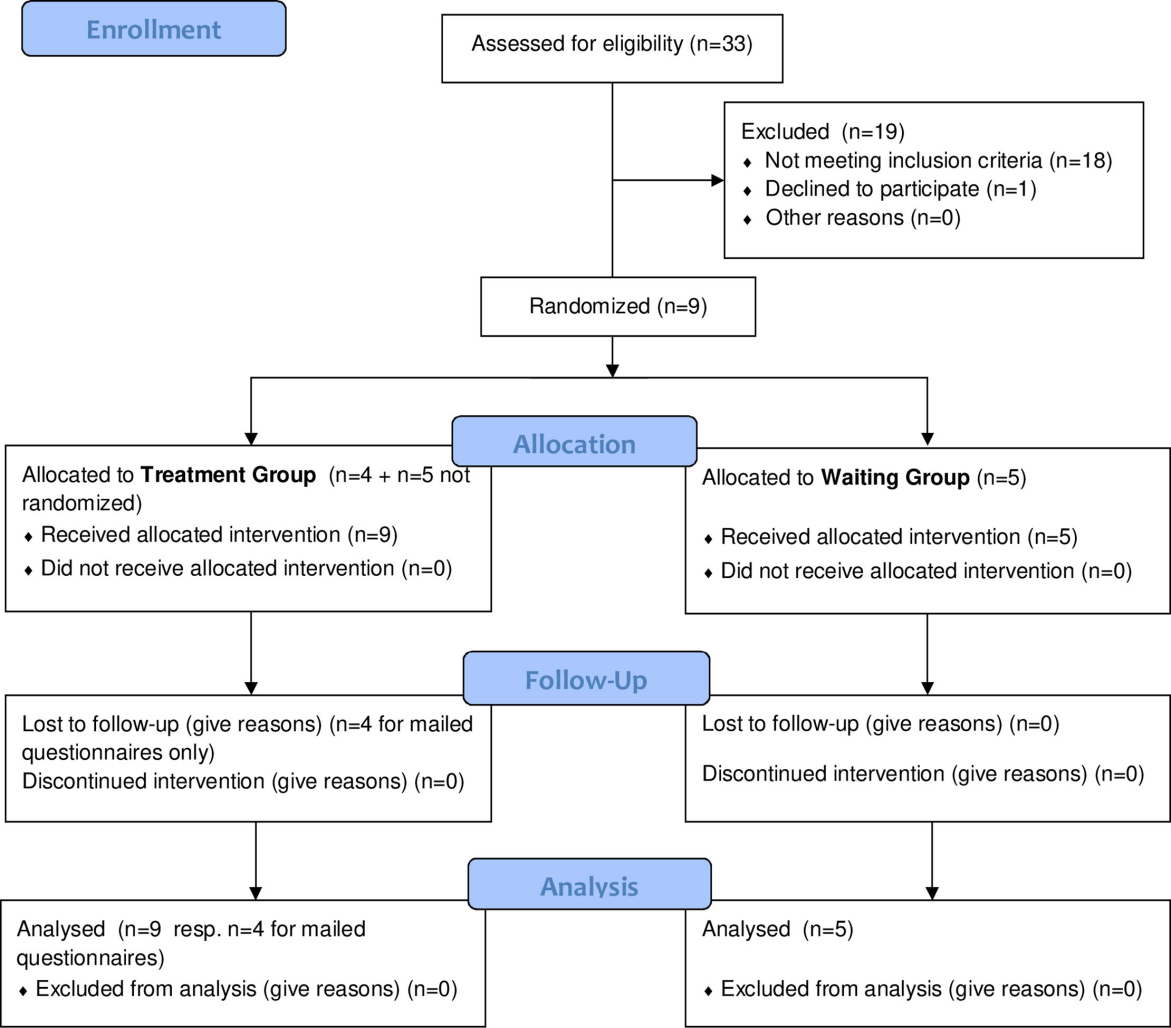
CONSORT flow chart.

Patients were referred by clerical assistants of the DGUV (German Social Accident Insurance) or by the psychotherapeutic outpatient clinic of the University of Würzburg. Due to the exploratory nature of this pilot study, no formal sample size calculation was performed. Patient flow is presented in Fig 1. In total 33 patients were screened, 16 patients were included in the study. Two patients dropped out (illness, only selective fear of driving trucks). The final sample consisted of 14 patients, 5 male and 9 female with a mean age of 40.36 years (*sd* = 8.57 yrs; *min* = 28 yrs, *max* = 53 yrs). Mean duration of driving fear and avoidance of driving was 18.0 months (*sd* = 17.0 months, *min* = 2.5 months, *max* = 49 months). Six patients were full avoiders and eight only avoided specific driving situations (such as highways or larger urban areas). Eight subjects were diagnosed having PTSD, six persons met the criteria for specific phobia. Four patients had started in vivo exposures before but discontinued very early on. All 14patients participated at the booster and the follow-up evaluation by telephone, but only 11 returned the follow-up questionnaires.

To control time effects, spontaneous remissions and possible effects of study-related activities, participants were randomly assigned to either a waiting group (WG, control group) or a treatment group (TG). However, due to logistic reasons and in order to facilitate the organization of the study, the last five patients were allocated to the TG without randomization according to their individual preferences. In consequence, the TG comprised of nine patients and the WG of only five patients. Due to the small size of the WG group, we do not differentiate between TG and WG in the analyses but do all analyses with the final sample of N = 14.

Inclusion criteria were: aged 18 to 63 years, valid driving license, severe symptoms of fear and avoidance of driving a car (complete avoidance or of specific car driving situations) for at least four weeks triggered by an accident or another critical event in traffic; regular driving experience prior to the triggering event; diagnosis of adjustment disorder, specific (isolated) phobia or PTSD; no previous exposure therapy (exceptions: mere exposure to sitting in a car or cancelled exposure); no or stable therapy with psychoactive drugs in the last four weeks.

Exclusion criteria were: alcohol/drug addiction, the influence of alcohol/drugs during the triggering event, suicidal tendencies, a positive pregnancy test (for females only), psychosis or other premorbid mental disorders or any contraindication to an exposure therapy. Prior to their participation, all eligible patients gave their written informed consent.

The study was approved by the ethics committee of the Bavarian Medical Association (No. 15077). Recruitment started in January 2016, the last follow-up assessment took place in August 2017.

### Design, procedure and treatment

The block therapy based on a treatment manual developed by the authors. Participants were randomly assigned either to WG or TG by drawing lots.

Table 1 gives a detailed overview of the treatment program.

**Table 1:** Overview of the therapy program.

4 weeks before	day 1 Mon	day 2 Tue	day 3 Wed	day 4 Thu	day 5 Fri	day 9 Tue	days 10–12 Wed–Fri	+ 6 weeks	+ 12 weeks
screening call	medical examination	psycho-therapeutic session I	psycho-therapeutic session II	simulator: exposure 2 (medium hierarchy level)	simulator: exposure 4 (repetition of scenarios from medium and upper level)	therapy finished	therapy finished	booster call	follow-up call evaluation by post post-measurement (questionnaires)
	psycho-therapeutic assessment with pre- measurement	familiarizing with the simulator	simulator: exposure 1 (lower hierarchy level)	simulator: exposure 3 (upper hierarchy level)	BAT psycho-therapeutic closing session				

The psychotherapeutic assessment at day 1 was supposed to establish a sustainable relationship between patient and therapist: Relevant data (accident event, symptoms, “hot spots” etc.) were collected and the diagnosis was confirmed. In contrast to the study protocol the driving test prior to treatment was only based on the patient´s report because of a recommendation of the project’s advisory board of the German statutory accident insurance (DGUV).

The psychotherapeutic sessions at day 2 included psychoeducation about driving fear, the presentation of the explanatory model and the illustration of the treatment rationale. An individual anxiety hierarchy was established which was used for the proceeding of the exposure sessions. Patients were familiarized with the simulator in order to reduce possible symptoms of simulator sickness and to practice handling the simulator vehicle (steering, accelerating and braking). In sum, five exposure sessions were conducted from day 3 to 5.

During the last exposure session, routes of the medium and upper level were repeated. Afterwards, the behavioral avoidance test took place in real traffic. In the psychotherapeutic closing session, the contents of the therapy and individual achievements were summarized. Methods for relapse prophylaxis were developed and exercises for the following week were recommended.

Six resp. twelve weeks follow-up: The therapist conducted a booster resp. follow-up call to determine the maintenance of treatment success and refresh the patients’ skills regarding dealing with their fear. Participants were asked to send back evaluation and symptoms questionnaires (post-measurement).

### Driving simulator and virtual environments

**Fig 2:**
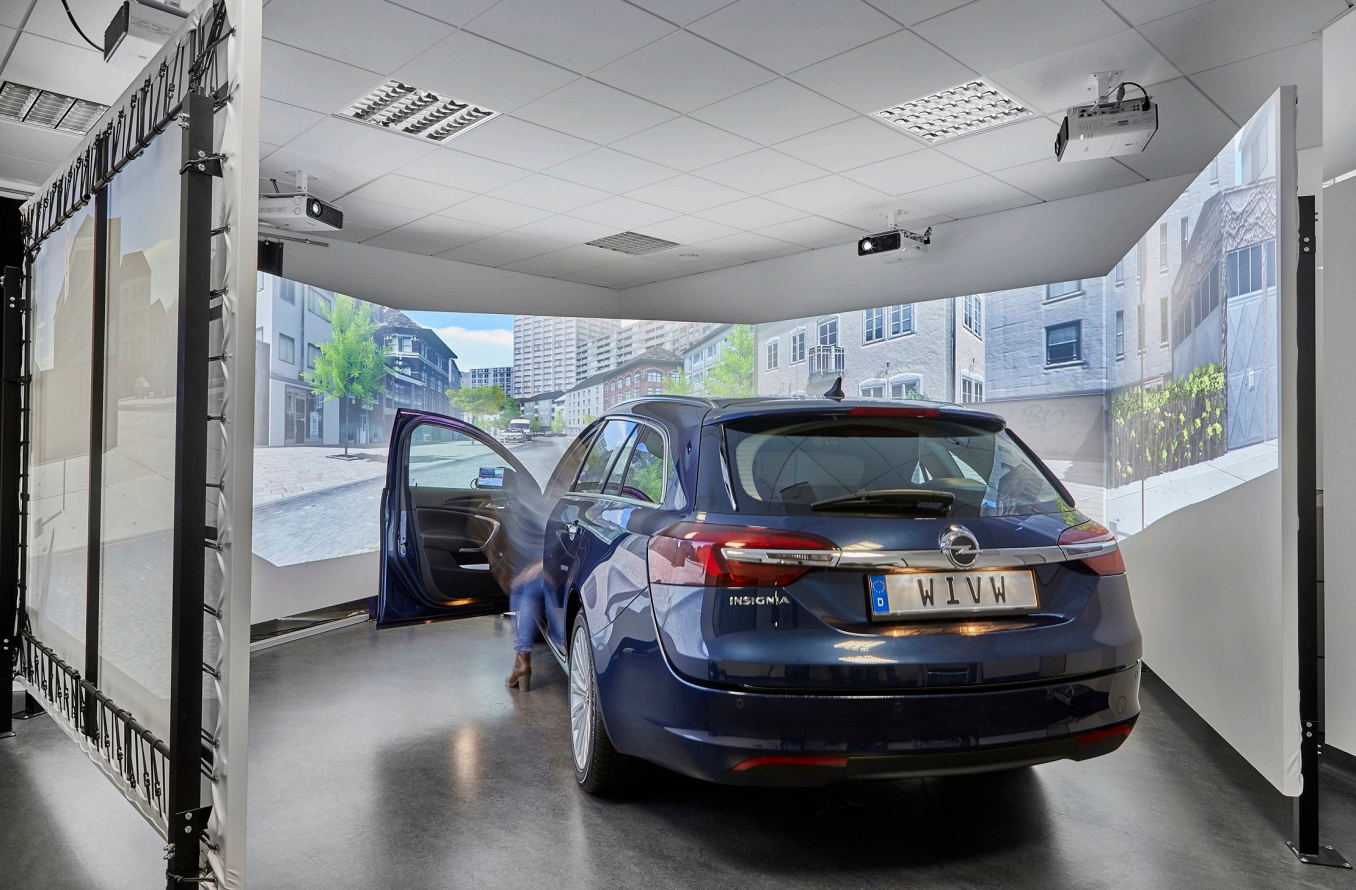
Static driving simulator of WIVW.

VRET sessions were conducted in the high-fidelity fixed base driving simulator of the Würzburg Institute for Traffic Sciences (WIVW, see Fig 2).

**Fig 3:**
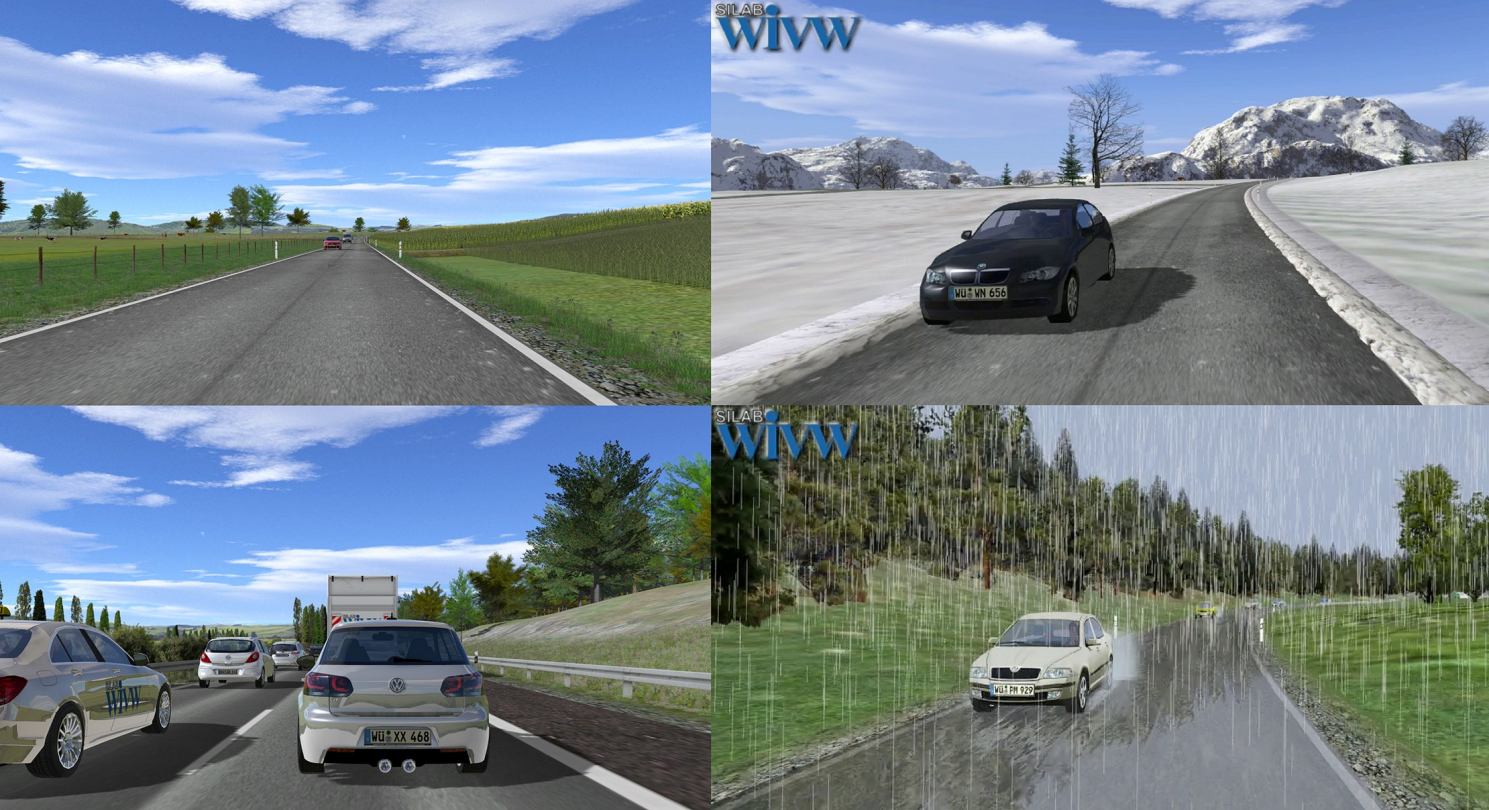
Exemplary virtual scenarios for different hierarchical levels.

The visual system comprises five image channels that provide a view of 300° horizontally and 47° vertically as well as a four-channels sound system. Rear view and outside mirrors are simulated by LCD-displays. The mockup is a real Opel Insignia with automatic transmission. The psychotherapist could accompany the exposure as a passenger or in a separate room next to the simulator which is connected to the patient by microphone, allowing talking to the patients during the drives. The simulation was run by the software SILAB^®^ (www.wivw.de, Fig 3).

The driving scenarios were specifically designed for this study and tailored to the patients’ individual anxiety hierarchy. This hierarchy was elaborated with the psychotherapist in the preparative sessions. For each VRE session, up to four scenarios were implemented and provided, beginning with situations on the lower anxiety level. All scenarios were programmed to be driven in an infinite loop. The duration of each drive depended on the current level of anxiety of the individual patient. During the drives, patients were repeatedly asked to assess their current anxiety on the subjective units of distress (SUD) scale (0 = no distress, totally relaxed; 10 = highest anxiety, loss of control). A reduction of at least two points on this scale was required to finish a scenario and to move on to the next scenario with a higher anxiety level. Consequently, there were major inter-individual differences with respect to the content as well as the duration of the exposure.

### Outcome measures

The primary outcome criterion was the successful completion of driving tasks in the final real driving test with the driving instructor. These tasks were considered not manageable before the treatment by the patient in the reported driving test which consisted of the following seven questions:

“If you had the chance to drive with a driving instructor right now, would you … (1) sit behind the wheel?” … (2) drive around a parking area?” … (3) drive around the block?”… (4) drive on a rural road?” … (5) drive on a main road with two lanes?” … (6) drive on a highway?” … (7) drive through an urban area (Würzburg, Germany)?”

If the answer was “yes”, patients rated the level of anticipated anxiety on the SUD scale.

For the real driving test after the treatment (BAT), only those tasks that have been reported to be avoided in the psychotherapeutic assessment were selected. However, for logistic reasons no more than four tasks could be realized. Patients were considered to be successfully treated (treatment responders) if they mastered at least one of these tasks in the final BAT.

Anxiety and habituation during the VRET sessions were assessed by ratings on the SUD scale as well as the heart rate. Simulator sickness was assessed before and after each exposure session according to Kennedy, Lane, Berbaum and Lilienthal [28].

Driving performance in the BAT was assessed by the driving instructor on an ordinal rating scale, commonly used for official driving tests to evaluate driving ability in Germany ([29] 0: normal, 1: slightly conspicuous, 2: substantially conspicuous, 3: severely conspicuous). A rating of 0 or 1 was defined as adequate driving performance.

Maintenance or extension of treatment success was assessed by the follow-up phone call twelve weeks after the treatment. For some analyses patients were subdivided into full- vs. part- vs. non-responders. Full responders were defined as patients who had mastered the final BAT with an adequate driving performance and could maintain their success until the follow-up phone call. Part responders were those who had mastered in the final BAT at least one task originally reported to be avoided, but either their driving performance had not been adequate as assessed by the driving instructor or they had not been able to maintain their success. Non-responders were defined as persons who did not master any of the tasks primarily reported to be avoided.

Phobic avoidance by means of the “Accident Fear Questionnaire, AFQ”[30], PTSD symptoms by means of the “Posttraumatic Stress Scale Self-Report PSS-SR” [31], depression by means of the “Beck-Depression-Inventory II (BDI-II)” [32], and anxiety by means of the “Beck-Anxiety-Inventory (BAI)” [33]were assessed before (psychotherapeutic assessment I) and after the treatment (follow-up).

Age, sex, duration of symptoms (fear and avoidance), initial preference of VRET versus in vivo exposure (yes vs. no), and aspects of therapy motivation as assessed by the FPTM (“Fragebogen zur Psychotherapiemotivation”) [34] were analyzed as potential predictors of treatment success.

### Statistical analysis

Generally, due to the small sample size and a lack of normal distributions, nonparametric tests were selected. Due to the exploratory character of this pilot study, a possible alpha inflation was disregarded. The same holds true for an estimation of effect sizes and the computation of confidence intervals. A significance level of 5% was set for all analyses.

To assess changes of SUD-ratings and heart rate over the four VRET sessions, Friedman tests were performed. Significant effects were followed by pairwise Wilcoxon signed rank tests. Changes *within* VRET sessions (comparisons of maximum and end values for both SUD and heart rate) were assessed by means of Wilcoxon signed rank tests.

Only descriptive percentages were calculated for the single tasks. The same holds true for driving performance in the BAT, maintenance of success in the follow-up phone call and evaluation data.

Differences of scores of the AFQ, the BDI-II, the BAT and the PSS-SR for the treatment group by comparing before vs. after the treatment were tested by Wilcoxon signed rank tests.

The analyses were performed by the computer software IBM SPSS Statistics for Windows (version 20.0).

## Results

### Behavioral avoidance test (BAT)

The VRET was helpful to overcome driving fear and avoidance as indicated by the performance on the behavioral driving test after the therapy (see Table 2).

**Table 2:** Number of patients (and percentages) who anticipated mastering the various tasks in the reported BAT before the treatment, and number of patients who actually mastered it according to the real BAT after treatment*.

	n (%) before	n (%) after
1. sit behind the wheel	12 (86%)	14 (100%)
2. drive around parking area	10 (71%)	14 (100%)
3. drive around the block	8 (57%)	14 (100%)
4. drive on rural road	5 (36%)	13 (93%)
5. drive on main road (2 lanes)	1 (7%)	11 (79%)
6. drive on highway	0 (0%)	6 (43%)
7. drive through urban area	2 (14%)	8 (57%)

*If patients were not exposed post-treatment to the appropriate task avoided pre-treatment it was assumed that they would still have avoided it (conservative assumption).

At pre-treatment, 43% of the patients were full avoiders. In the post-treatment BAT, all 14 patients mastered at least one of the tasks they had previously reported to avoid. 13 of the 14 patients even completed all of the offered driving tasks.

According to the driving instructor, 10 of the 14 patients (71%) showed an adequate driving performance during the BAT (4 normal, 6 slightly conspicuous). Three patients drove substantially conspicuous, one even severely conspicuous. In two cases the driving instructor had to intervene to avoid critical situations. Mostly, driving performance was rated as being conspicuous because of an anxious driving style (driving too slowly, unsteady longitudinal control, overcautious securing behavior).

13 out of 14 (93%) patients could maintain or even extend their treatment success until the follow-up phone call. 57% of the patients even reported that by then they were able to complete all driving tasks. Only one patient did not maintain the success and regressed to avoid all tasks previously mastered in the BAT. One patient mostly maintained his success by still driving three of the four tasks completed in the BAT. Another patient avoided urban areas again but extended his success to highways instead, which had not been offered in the BAT because of time constraints.

### VRET sessions

On average, three drives were completed per exposure session (sd = 0.76; *min* = 2, *max* = 4) with a mean duration of 19.6 minutes per drive (*sd* = 11.6, *min* = 5, *max* = 60 minutes). The large range in duration was due to inter-individual differences in anxiety and habituation.

#### Anxiety ratings

**Fig 4:**
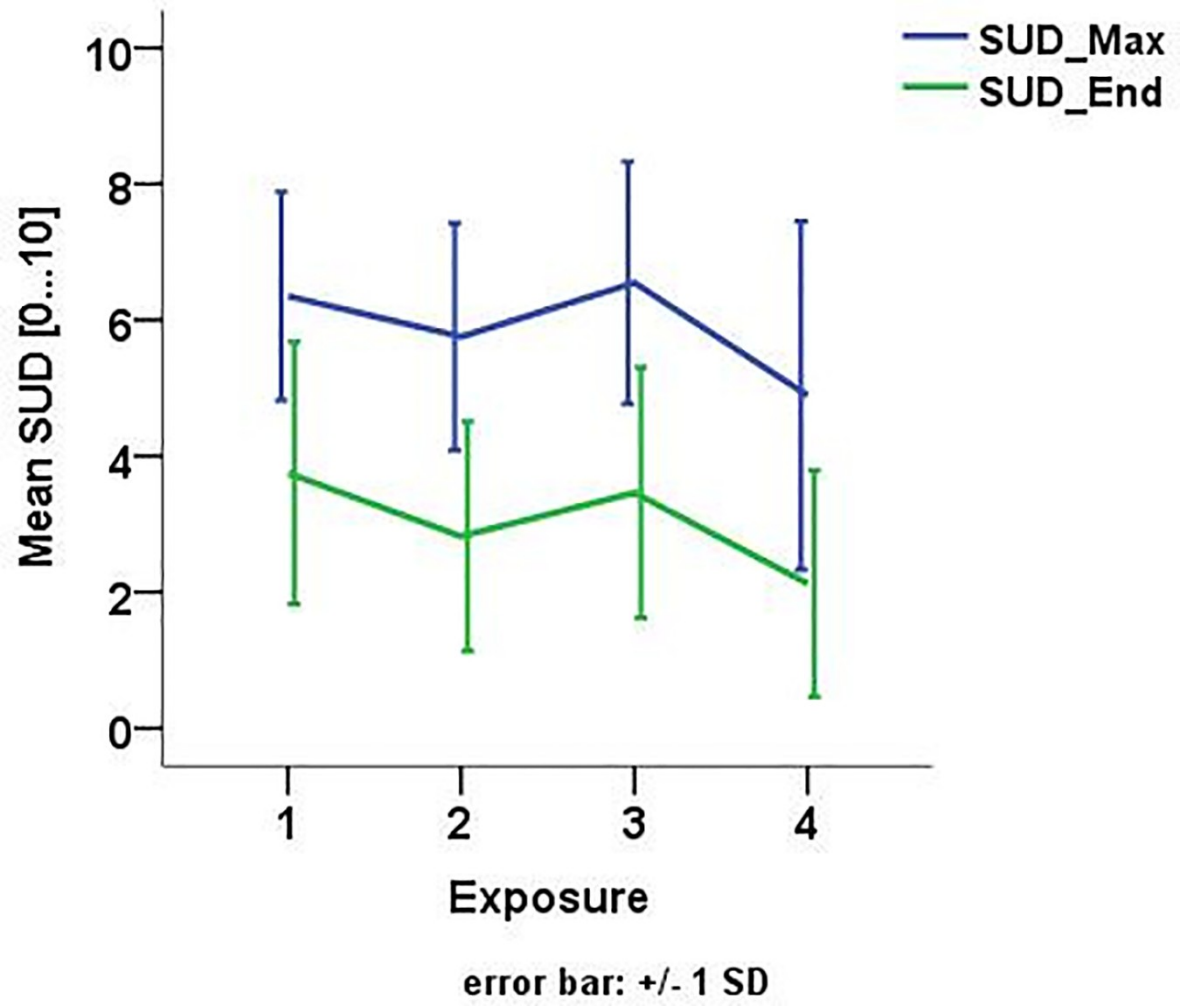
Mean maximum and end SUD score over the four exposure sessions.

SUD scores of 6 or higher were reached by 93% of the participants in the first session but only by 79%, 86%, 71% resp. in the following sessions. Similarly, mean maximum SUD scores of each session (see Fig 4) progressively decreased over sessions (Friedman test Χ^2^ = 14.85; *p* = .002; see Fig 4) with higher anxiety ratings in the first compared to the second (*Z* = -1.96, *p* = .050) and fourth session (*Z* = -1.99, *p* = .046). The mean maximum SUD in the third session, when scenarios of upper hierarchy levels were driven, was as high as in the first session (*Z* = -0.76; *p* = .861). Hence, VR drives elicited considerable anxiety which generally decreased between sessions.

All patients habituated successfully within the sessions i.e. fear ratings decreased significantly by at least 2 points on the SUD scale during all the virtual drives and within each session (Wilcoxon signed rank test: session 1: *Z* = -3.30; *p* = .001; session 2: *Z* = -3.30; *p* = .001; session 3: *Z* = -3.30; *p* = .001; session 4: *Z* = -3.30; *p* = .001; see also Fig 4).

The mean baseline heart rate before exposure was 69.70 bpm (*sd* = 10.75) with no significant difference between the sessions (Χ^2^ = 2.92; *p* = .572). Overall, baseline corrected heart rates indicate physiological arousal during virtual drives, but no clear habituation between sessions (see Fig 4) as the mean maximum heart rate did not significantly differ between sessions (Χ^2^ = 5.90, *p* = .117).

**Fig 5:**
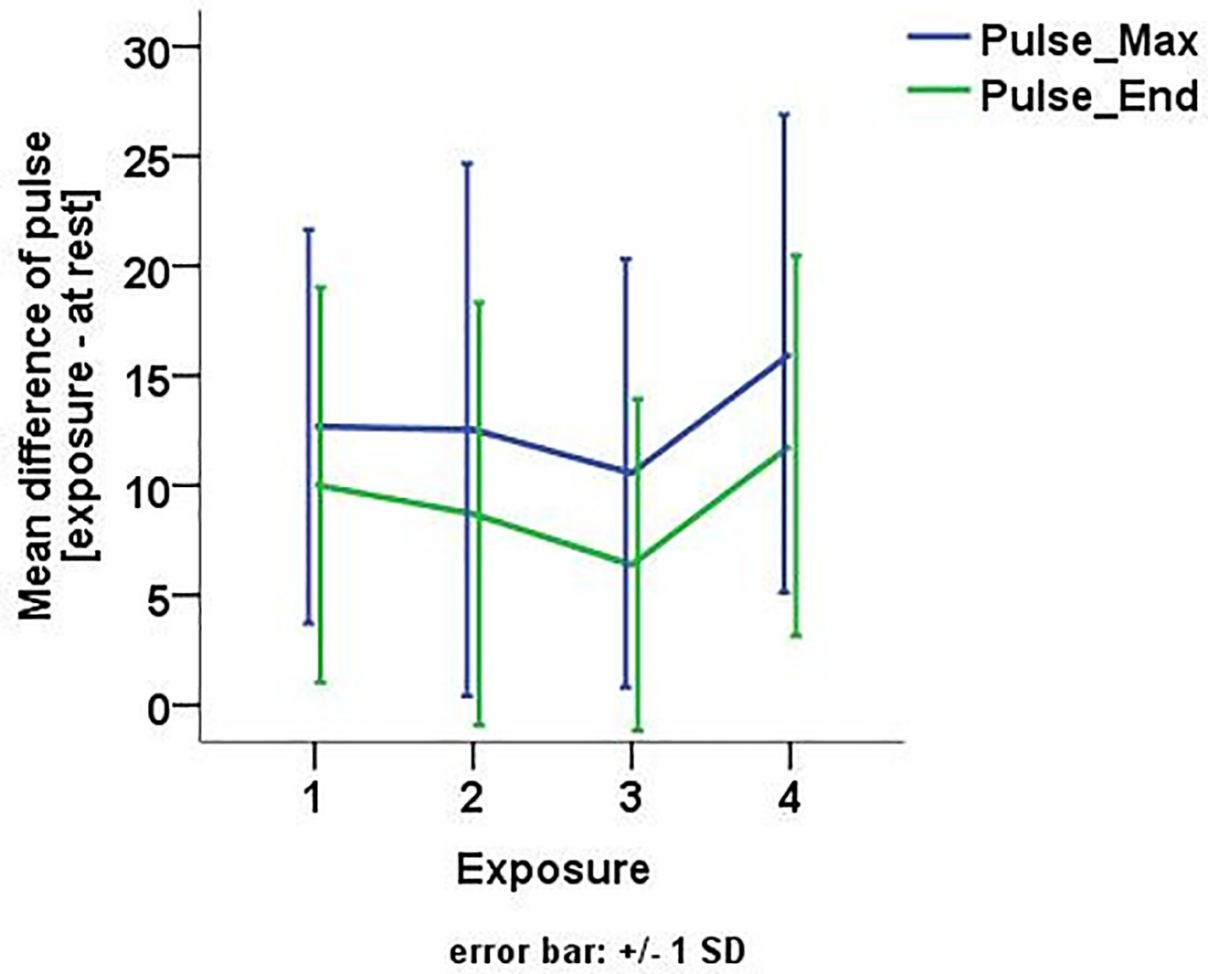
Mean maximum heart rate and mean heart rate at the end over the four exposure sessions. Values are presented after a baseline correction with positive values indicating an increase compared to the heart rate during relaxation.

Within all sessions, we observed significant differences between the maximum heart rate and the heart rate at the end of the session (Wilcoxon signed rank test: session 1: *Z* = -2.98; *p* = .003; session 2: *Z* = -2.10; *p* = .035; session 3: *Z* = -2.20; *p* = .028; session 4: *Z* = -2.36; *p* = .018; see also Fig 5).

### Psychopathological symptoms

Questionnaire data assessed before and after the treatment indicate a slight improvement of psychopathological symptoms (Table 3).

**Table 3:** Pretreatment and post-treatment outcome measures of questionnaires.

	n*	pre m (sd)	post m (sd)	Wilcoxon test
AFQ	10	31.3 (12.5)	21.7 (20.9)	*Z* = -1.66, *p* = .097 (p < .010)
PSS-SR	10	19.5 (12.8)	10.8 (9.0)	*Z* = -1.99; *p* = .047 (p < .050)
BDI-II	11	14.8 (9.4)	12.9 (10.5)	*Z* = -.51, *p* = .61 (n.s.)
BAI	11	24.0 (12.9)	20.2 (16.1)	*Z* = -.59, *p* = .55 (n.s)

* Follow-up data was missing for three patients, another patient could not execute the AFQ and the PSS-SR due to insufficient verbal skills.

For the PSS-SR, a significant mean reduction of about nine points and for the AFQ, a descriptive improvement of 10 points was observed. Given the small sample size these differences deserve consideration and may reflect clinical improvement. For the Becks Inventories, no meaningful changes were observed.

### Evaluation

Twelve weeks after the therapy, patients received an evaluation questionnaire. Data of three participants, who were full responders and reported high satisfaction with the treatment at the last personal contact, are missing. Thus, no positive distortion of the following data is assumed.

**Fig 6:**
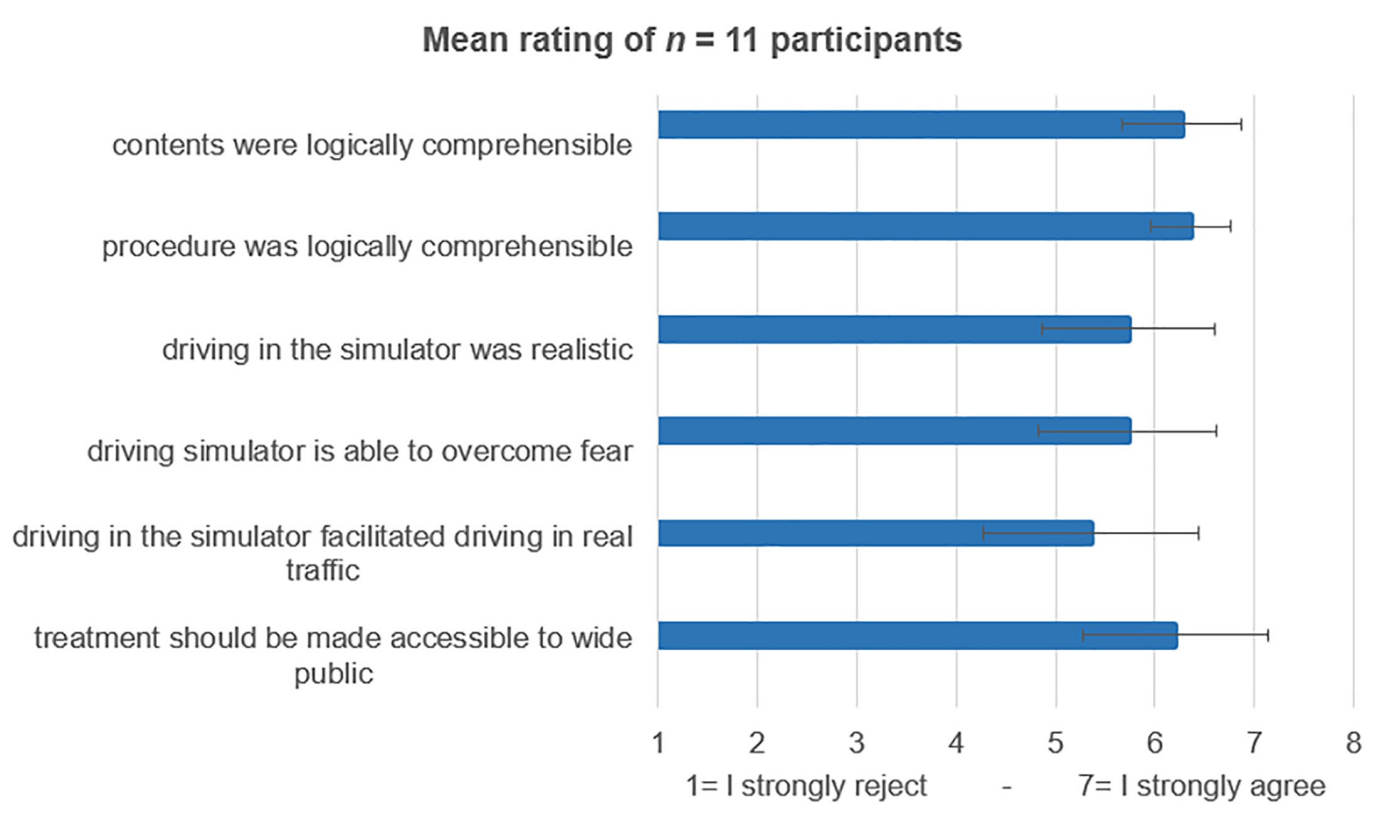
Mean rating of patients on the various items of the evaluation questionnaire.

Participants rated the tasks (*m* = 3.91, *sd* = 0.54; scale ranging from 1 = too easy to 7 = too difficult), the procedural speed (*m* = 4.09, *sd* = 0.94; scale ranging from 1 = too slow to 7 = too fast) and the time frame of treatment (*m* = 3.64, *sd* = 1.03; scale ranging from 1 = too short to 7 = too long) as appropriate. The contents of treatment and the use of the simulator were evaluated very positively (see Fig 6).

The general assessment rating (ranging from 1 = very good to 6 = insufficient/failed) was good to very good (*m* = 1.63; *sd* = 0.89). Most patients were overwhelmed by their success after the BAT.

## Discussion and conclusions

The present study examined the efficacy of a manualized VRET in a sample of 14 patients suffering from driving fear after a traffic accident or another critical traffic event. This study is the first with a BAT to evaluate treatment success. The distinct individualization of driving scenarios resulted in high levels of reported anxiety during the VRET sessions and sufficient habituation within and across sessions. After the VRET treatment, driving fear and avoidance were markedly reduced: All patients were able to master at least one driving task in the BAT that they reported to avoid prior to treatment. 13 of 14 patients even mastered all of the offered driving tasks successfully and 71% of them showed adequate driving behavior. All but one patient (93%) could maintain or even extend treatment success until the phone call at twelve weeks follow-up. Nine patients were considered “full responders” as they completed the BAT with adequate driving behavior and could maintain this success until the follow-up, the remaining five patients were “part responders” as they successfully completed the BAT but showed some impairment in driving performance and/or could not maintain their BAT success until the follow-up.

In contrast to other studies [25, 26], we had a rather weak criterion for proceeding from one VRET scenario to the next, more anxiety-provoking scenario. The realized criterion (an anxiety reduction of at least two points on the SUD scale to a level of four or below) allows more flexibility and takes into account individual response biases. Thereby, it is still sufficient to allow patients to experience habituation effects and to motivate them for the next exposure level.

As indicated by the AFQ and PSS-SR measures before and after the therapy, there were also a reduction of phobic avoidance by trend and a significant amelioration of PTSD symptoms. Even though these findings are not impressive, they deserve clinical consideration specifically given the small sample size.

There are some limitations of the study: The small sample size of 14 patients is a methodological limitation, leading to limited statistical analysis and explanatory power of the results. Furthermore, there was no proper randomized control group and the size of the WG (*n* = 5) is not satisfactory. A strong limitation of this study is that for ethical reasons, no real driving test could be offered prior to treatment. Thus, the comparison of the data surveyed by the reported driving test before treatment and the outcome in the real driving test at the end is only a compromise solution that is not fully scientifically sound. With respect to the follow-up evaluation, it cannot be excluded that the real drive with the driving instructor had a treatment effect for the maintenance of success (in terms of an in vivo exposure).

The applied hierarchical exposure stands in contrast to the recommended “flooding” treatment for specific phobias and PTBS. However, we decided to realize hierarchical exposure because of a reduced risk of reluctance and dropping out [14] and in favor of preventing simulator sickness, which is more likely for more demanding driving maneuvers [35].

In conclusion, this study indicates that VRET with driving simulation is highly efficient to treat patients with driving fear and avoidance. Treatment success was evident in post-treatment real driving and was maintained at least for three months. However, further studies are needed to evaluate VRET efficacy for driving fear with a randomized controlled trial, preferably with a waiting list group with longer delay or an active control group (e.g. exposure therapy in vivo, in sensu or just a talking therapy) in order to differentiate precisely between mere VRET effects and general treatment effects.

## Supporting information

S1 Checklist TREND statement checklist. (DOCX)

S1 Dataset Minimal anonymized data set. (XLSX)

S1 Variables Description Description if the variables of the S1 Dataset. (PDF)

S1 Protocol (PDF)

S2 Protocol (PDF)
