# Supplementary material for: Treating patients with driving phobia by virtual reality exposure therapy – a pilot study
Source: PLoS One. 2020 Jan 7;15(1):e0226937. doi: 10.1371/journal.pone.0226937 (PMC6946146; doi:10.1371/journal.pone.0226937)
Supplement: S1 Variables Description — (PDF) [file pone.0226937.s003.pdf]

| Code | Description of Item | Range | Labels |
|---|---|---|---|
| **Statistics** | | | |
| RandomNo | Randomisation Number of patient | | |
| Group | Experimental Group | | 1=Treatment Group<br>2=Waiting Group |
| Sex | Sex | | 1=male<br>2=female |
| ExpoSession | Exposure session | 1 – 4 | |
| Number | Number of drives per exposure session | | |
| Duration | Total duration of drives in [min] per driver and exposure session | | |
| Symptoms_Duration | [months] | | |
| Responder | Responder: completed BAT with adequate driving behavior and could maintain success until the follow-up | | 1=yes<br>0=no |
| S_Preference_Expo | Preference of Exposure before treatment (screening) | | 0=in vivo<br>1=virtual<br>2=combination of both |
| **Psychological Questionnaires** | | | |
| afq_pa_pre | Score AFQ prior to treatment (phobic avoidance items) | | |
| afq_pre1_WG | Score AFQ anamnesis 1 | | |
| afq_pre2_WG | Score AFQ anamnesis 2 | | |
| afq_pa_post | Score AFQ post exposure | | |
| afq_pa_diff | Difference of AFQ score AFQ_pa_pre - afq_pa_post | | |
| pss_sr_pre | Score PSS-SR prior to treatment | | |
| pss_sr_post | Score PSS-SR post treatment | | |
| pss_sr_diff | Difference of PSS-SR score pss_sr_prä - pss_sr_post | | |
| bai_pre | Score BAI prior to treatment, anamnesis 1 | | |
| bai_pre2 | Score BAI prior to treatment, anamnesis 2 (WG) | | |
| bai_post | Score BAI post treatment | | |
| bai_diff | Difference BAI score bai_pre – bai_post | | |
| bdi_pre | Score BDI-II prior to treatment, anamnesis 1 | | |
| bdi_pre2 | Score BDI-II prior to treatment, anamnesis 2 (WG) | | |
| bdi_post | Score BDI-II post treatment | | |
| bdi_diff | Difference score BDI-II bdi_pre – bdi_post | | |
| FPTM_strain | Score FPTM psychological strain | | |
| FPTM_hope | Score FPTM hope for improvement | | |

| | | | |
|---|---|---|---|
| FPTM_denial | Score FPTM denial of psychological need for care | | |
| FPTM_knowledge | Score FPTM knowledge about therapy | | |
| FPTM_initiative | Score FPTM patient's effort to receive the therapy | | |
| FPTM_care | Score FPTM perceived attention by others due to symptoms | | |
| **Exposure Sessions** | | | |
| ES1_S1_SUD_Max | ES1: Exposure Session 1; S1: Scenario 1; highest SUD during this drive | Subjective units of distress (SUD) scale:<br>0=no distress to 10=highest anxiety, loss of control | |
| ES1_S1_Pulse_Max | ES1: Exposure Session 1; S1: Scenario 1; highest pulse during this drive | | |
| ES1_S1_SUD_End | ES1: Exposure Session 1; S1: Scenario 1; SUD at the end of this drive | Subjective units of distress (SUD) scale:<br>0=no distress to 10=highest anxiety, loss of control | |
| ES1_S1_Pulse_End | ES1: Exposure Session 1; S1: Scenario 1; pulse at the end of the drive | | |
| ES1_S2_SUD_Max | ES1: Exposure Session 1; S2: Scenario 2; highest SUD during this drive | Subjective units of distress (SUD) scale:<br>0=no distress to 10=highest anxiety, loss of control | |
| ES1_S2_Pulse_Max | ES1: Exposure Session 1; S2: Scenario 2; highest pulse during this drive | | |
| ES1_S2_SUD_End | ES1: Exposure Session 1; S2: Scenario 2; SUD at the end of this drive | Subjective units of distress (SUD) scale:<br>0=no distress to 10=highest anxiety, loss of control | |
| ES1_S2_SUD_End | ES1: Exposure Session 1; S2: Scenario 2; pulse at the end of the drive | | |
| ES1_S3_SUD_Max | ES1: Exposure Session 1; S3: Scenario 3; highest SUD during this drive | Subjective units of distress (SUD) scale:<br>0=no distress to 10=highest anxiety, loss of control | |
| ES1_S3_Pulse_Max | ES1: Exposure Session 1; S3: Scenario | | |

| | | | |
|---|---|---|---|
| | 3; highest pulse during this drive | | |
| ES1_S3_SUD_End | ES1: Exposure Session 1; S3: Scenario 3; SUD at the end of this drive | Subjective units of distress (SUD) scale:<br>0=no distress to 10=highest anxiety, loss of control | |
| ES1_S3_SUD_End | ES1: Exposure Session 1; S3: Scenario 3; pulse at the end of the drive | | |
| ES1_S4_SUD_Max | ES1: Exposure Session 1; S4: Scenario 4; highest SUD during this drive | Subjective units of distress (SUD) scale:<br>0=no distress to 10=highest anxiety, loss of control | |
| ES1_S4_Pulse_Max | ES1: Exposure Session 1; S4: Scenario 4; highest pulse during this drive | | |
| ES1_S4_SUD_End | ES1: Exposure Session 1; S4: Scenario 4; SUD at the end of this drive | Subjective units of distress (SUD) scale:<br>0=no distress to 10=highest anxiety, loss of control | |
| ES1_S4_Pulse_End | ES1: Exposure Session 1; S4: Scenario 4; pulse at the end of the drive | | |
| ES1_PulseBase | ES1: Exposure Session 1; Pulse baseline during relaxation at the end of the session. | | |
| ES2_S[1-4]_SUD_Max | ES2: Exposure Session 2; S[1-4]: Scenarios 1-4; highest SUD during each of the drives | | |
| ES2_S[1-4]_Pulse_Max | ES2: Exposure Session 2; S[1-4]: Scenarios 1-4; highest pulse during each of these drives | | |
| ES2_S[1-4]_SUD_End | ES2: Exposure Session 2; S[1-4]: Scenarios 1-4; SUD at the end of each drive | | |
| ES2_S[1-4]_Pulse_End | ES2: Exposure Session 2; S[1-4]: Scenarios 1-4; pulse at the end of each drive | | |
| ES2_PulseBase | ES2: Exposure Session 2; Pulse baseline during relaxation at the end of the session. | | |
| ES3_S[1-4]_SUD_Max | ES3: Exposure Session 3; S[1-4]: Scenarios 1-4; highest SUD during each of the drives | | |
| ES3_S[1-4]_Pulse_Max | ES3: Exposure Session 3; S[1-4]: Scenarios 1-4; highest pulse during each of these drives | | |

| | | | |
|---|---|---|---|
| ES3_S[1-4]_SUD_End | ES3: Exposure Session 3; S[1-4]: Scenarios 1-4; SUD at the end of each drive | | |
| ES3_S[1-4]_Pulse_End | ES3: Exposure Session 3; S[1-4]: Scenarios 1-4; pulse at the end of each drive | | |
| ES3_PulseBase | ES3: Exposure Session 3; Pulse baseline during relaxation at the end of the session. | | |
| ES4_S[1-4]_SUD_Max | ES4: Exposure Session 4; S[1-4]: Scenarios 1-4; highest SUD during each of the drives | | |
| ES4_S[1-4]_Pulse_Max | ES4: Exposure Session 4; S[1-4]: Scenarios 1-4; highest pulse during each of these drives | | |
| ES4_S[1-4]_SUD_End | ES4: Exposure Session 4; S[1-4]: Scenarios 1-4; SUD at the end of each drive | | |
| ES4_S[1-4]_Pulse_End | ES4: Exposure Session 4; S[1-4]: Scenarios 1-4; pulse at the end of each drive | | |
| ES4_PulseBase | ES4: Exposure Session 4; Pulse baseline during relaxation at the end of the session. | | |
| mean_max_SUD_ES1 | Mean maximum SUD during Exposure session 1 (ES1_S1_SUD_Max, ES1_S2_SUD_Max, ES1_S3_SUD_Max, ES1_S4_SUD_Max) | | |
| mean_max_SUD_ES2 | Mean maximum SUD during Exposure session 2 (ES2_S1_SUD_Max, ES2_S2_SUD_Max, ES2_S3_SUD_Max, ES2_S4_SUD_Max) | | |
| mean_max_SUD_ES3 | Mean maximum SUD during Exposure session 3 (ES3_S1_SUD_Max, ES3_S2_SUD_Max, ES3_S3_SUD_Max, ES3_S4_SUD_Max) | | |
| mean_max_SUD_ES4 | Mean maximum SUD during Exposure session 4 (ES4_S1_SUD_Max, ES4_S2_SUD_Max, ES4_S3_SUD_Max, ES4_S4_SUD_Max) | | |
| mean_end_SUD_ES1 | Mean end SUD of exposure session 1 (ES1_S1_SUD_End, ES1_S2_SUD_End, ES1_S3_SUD_End, ES1_S4_SUD_End) | | |
| mean_end_SUD_ES2 | Mean end SUD of exposure session 2 (ES2_S1_SUD_End, ES2_S2_SUD_End, ES2_S3_SUD_End, ES2_S4_SUD_End) | | |
| mean_end_SUD_ES3 | Mean end SUD of exposure session 3 | | |

| | | | |
|---|---|---|---|
| | (ES3_S1_SUD_End, ES3_S2_SUD_End, ES3_S3_SUD_End, ES3_S4_SUD_End) | | |
| mean_end_SUD_ES4 | Mean end SUD of exposure session 4 (ES4_S1_SUD_End, ES4_S2_SUD_End, ES4_S3_SUD_End, ES4_S4_SUD_End) | | |
| ES1_S[1-4]_Puls_max_diff | Baseline corrected maximum heart rate:<br>ES1: Exposure 1; S[1-4]: Scenarios 1-4; Difference (ES1_S[1-4]_Puls_Max - ES1_PulsBase) | | |
| ES2_S[1-4]_Puls_max_diff | Baseline corrected maximum heart rate:<br>ES2: Exposure 2; S[1-4]: Scenarios 1-4; Difference (ES2_S[1-4]_Puls_Max - ES2_PulsBase) | | |
| ES3_S[1-4]_Puls_max_diff | Baseline corrected maximum heart rate:<br>ES3: Exposure 3; S[1-4]: Scenarios 1-4; Difference (ES3_S[1-4]_Puls_Max - ES3_PulsBase) | | |
| ES4_S[1-4]_Puls_max_diff | Baseline corrected maximum heart rate:<br>ES4: Exposure 4; S[1-4]: Scenarios 1-4; Difference (ES4_S[1-4]_Puls_Max - ES4_PulsBase) | | |
| ES1_S[1-4]_Puls_End_diff | Baseline corrected heart rate at the end of each drive:<br>ES1: Exposure 1; S[1-4]: Scenarios 1-4; Difference (ES1_S[1-4]_Puls_end - ES1_PulsBase) | | |
| ES2_S[1-4]_Puls_End_diff | Baseline corrected heart rate at the end of each drive:<br>ES2: Exposure 2; S[1-4]: Scenarios 1-4; Difference (ES2_S[1-4]_Puls_end - ES2_PulsBase) | | |
| ES3_S[1-4]_Puls_End_diff | Baseline corrected heart rate at the end of each drive:<br>ES3: Exposure 3; S[1-4]: Scenarios 1-4; Difference (ES3_S[1-4]_Puls_end - ES3_PulsBase) | | |
| ES4_S[1-4]_Puls_End_diff | Baseline corrected heart rate at the end of each drive:<br>ES4: Exposure 4; S[1-4]: Scenarios 1-4; Difference (ES4_S[1-4]_Puls_end - ES4_PulsBase) | | |
| mean_max_Puls_ES1 | MEAN baseline corrected maximum heart rate per exposure session 1 (ES1_S1_Puls_max_diff, ES1_S2_Puls_max_diff, ES1_S3_Puls_max_diff, ES1_S4_Puls_max_diff) | | |
| mean_max_Puls_ES2 | MEAN baseline corrected maximum | | |

| | | | |
|---|---|---|---|
| | heart rate per exposure session 2 (ES2_S1_Puls_max_diff, ES2_S2_Puls_max_diff, ES2_S3_Puls_max_diff, ES2_S4_Puls_max_diff) | | |
| mean_max_Puls_ES3 | MEAN baseline corrected maximum heart rate per exposure session 3 (ES3_S1_Puls_max_diff, ES3_S2_Puls_max_diff, ES3_S3_Puls_max_diff, ES3_S4_Puls_max_diff) | | |
| mean_max_Puls_ES4 | MEAN baseline corrected maximum heart rate per exposure session 4 (ES4_S1_Puls_max_diff, ES4_S2_Puls_max_diff, ES4_S3_Puls_max_diff, ES4_S4_Puls_max_diff) | | |
| mean_End_Puls_ES1 | MEAN baseline corrected heart rate at the end of exposure session 1 (ES1_S1_Puls_End_diff, ES1_S2_Puls_End_diff, ES1_S3_Puls_End_diff, ES1_S4_Puls_End_diff) | | |
| mean_End_Puls_ES2 | MEAN baseline corrected heart rate at the end of exposure session 2 (ES2_S1_Puls_End_diff, ES2_S2_Puls_End_diff, ES2_S3_Puls_End_diff, ES2_S4_Puls_End_diff) | | |
| mean_End_Puls_ES3 | MEAN baseline corrected heart rate at the end of exposure session 3 (ES3_S1_Puls_End_diff, ES3_S2_Puls_End_diff, ES3_S3_Puls_End_diff, ES3_S4_Puls_End_diff) | | |
| mean_End_Puls_ES4 | MEAN baseline corrected heart rate at the end of exposure session 4 (ES4_S1_Puls_End_diff, ES4_S2_Puls_End_diff, ES4_S3_Puls_End_diff, ES4_S4_Puls_End_diff) | | |
| **Driving performance in the BAT** | | | |
| wheel_sud.0 | Hypothetical question during screening: “If you had the chance to drive with a driving instructor right now, would you sit behind the wheel?” | Subjective units of distress (SUD) scale: 0=no distress to 10=highest anxiety, loss of control; 11=would not do it | |
| wheel_dic.0 | Hypothetical SUD sitting behind the | | 0=not avoided |

| | | | |
|---|---|---|---|
| | wheel (screening) dichotomized | | (SUD <6)<br>1=avoided<br>(SUD ≥6) |
| wheel_sud.1 | Hypothetical question during anamnesis 1:<br>"If you had the chance to drive with a driving instructor right now, would you sit behind the wheel?" | SUD scale:<br>0=no distress to 10=highest anxiety, loss of control;<br>11=would not do it | |
| wheel_dic.1 | Hypothetical SUD sitting behind the wheel (anamnesis 1) dichotomized | | 0=not avoided (SUD <6)<br>1=avoided<br>(SUD ≥6) |
| wheel_sud.2 | Hypothetical question during anamnesis 2 (WG), anamnesis 1 (TG):<br>"If you had the chance to drive with a driving instructor right now, would you sit behind the wheel?" | SUD scale:<br>0=no distress to 10=highest anxiety, loss of control;<br>11=would not do it | |
| wheel_dic.2 | Hypothetical SUD sitting behind the wheel (anamnesis 2 (WG), anamnesis 1 (TG)) dichotomized | | 0=not avoided (SUD <6)<br>1=avoided<br>(SUD ≥6) |
| wheel_sud.3 | Hypothetical question post exposure:<br>"If you had the chance to drive with a driving instructor right now, would you sit behind the wheel?" | SUD scale:<br>0=no distress to 10=highest anxiety, loss of control;<br>11=would not do it | |
| wheel_dic.3 | Hypothetical SUD sitting behind the wheel (post exposure) dichotomized | | 0=not avoided (SUD <6)<br>1=avoided<br>(SUD ≥6) |
| wheel_dic.4 | SUD during BAT: sitting behind the wheel | | 0=not avoided (any more)<br>1=still avoided |
| wheel_dic.42 | SUD during BAT: sitting behind the wheel (1 also for tasks originally mastered) | | 0=not avoided (any more)<br>1=still avoided |
| wheel_dic.5 | Hypothetical SUD during booster interview: sitting behind the wheel | | 0=not avoided (any more)<br>1=still avoided |
| wheel_dic.6 | Hypothetical SUD during follow-up interview: sitting behind the wheel | | 0=not avoided (any more)<br>1=still avoided |
| parking_sud.0 | Hypothetical question during screening:<br>"If you had the chance to drive with a driving instructor right now, would | SUD scale:<br>0=no distress to 10=highest anxiety, loss of | |

| | | | |
|---|---|---|---|
| | you drive around a parking area?” | control;<br>11=would not do it | |
| parking_dic.0 | Hypothetical SUD driving around a parking area (screening) dichotomized | | 0=not avoided (SUD <6)<br>1=avoided (SUD ≥6) |
| parking_sud.[1-3] | See descriptions for wheel_sud | | |
| parking_dic.[1-6] | See descriptions for wheel_dic | | |
| block_sud.0 | Hypothetical question during screening:<br>“If you had the chance to drive with a driving instructor right now, would you drive around the block?” | SUD scale:<br>0=no distress to 10=highest anxiety, loss of control;<br>11=would not do it | |
| block_dic.0 | Hypothetical SUD driving around the block (screening) dichotomized | | 0=not avoided (SUD <6)<br>1=avoided (SUD ≥6) |
| block_sud.[1-3] | See descriptions for wheel_sud | | |
| block_dic.[1-6] | See descriptions for wheel_dic | | |
| ruralroad_sud.0 | Hypothetical question during screening:<br>“If you had the chance to drive with a driving instructor right now, would you drive on a rural road?” | SUD scale:<br>0=no distress to 10=highest anxiety, loss of control;<br>11=would not do it | |
| ruralroad_dic.0 | Hypothetical SUD driving on rural road (screening) dichotomized | | 0=not avoided (SUD <6)<br>1=avoided (SUD ≥6) |
| ruralroad_sud.[1-3] | See descriptions for wheel_sud | | |
| ruralroad_dic.[1-6] | See descriptions for wheel_dic | | |
| twolane_sud.0 | Hypothetical question during screening:<br>“If you had the chance to drive with a driving instructor right now, would you drive on a main road with two lanes?” | SUD scale:<br>0=no distress to 10=highest anxiety, loss of control;<br>11=would not do it | |
| twolane_dic.0 | Hypothetical SUD driving on main road with two lanes (screening) dichotomized | | 0=not avoided (SUD <6)<br>1=avoided (SUD ≥6) |
| twolane_sud.[1-3] | See descriptions for wheel_sud | | |
| twolane_dic.[1-6] | See descriptions for wheel_dic | | |
| highway_sud.0 | Hypothetical question during screening:<br>“If you had the chance to drive with a driving instructor right now, would | SUD scale:<br>0=no distress to 10=highest anxiety, loss of | |

| | | | |
|---|---|---|---|
| | you drive on a highway?” | control;<br>11=would not do it | |
| highway_dic.0 | Hypothetical SUD driving on a highway (screening) dichotomized | | 0=not avoided (SUD <6)<br>1=avoided (SUD ≥6) |
| highway_sud.[1-3] | See descriptions for wheel_sud | | |
| highway_dic.[1-6] | See descriptions for wheel_dic | | |
| urban_sud.0 | Hypothetical question during screening:<br>“If you had the chance to drive with a driving instructor right now, would you drive on an urban area?” | SUD scale:<br>0=no distress to 10=highest anxiety, loss of control;<br>11=would not do it | |
| urban_dic.0 | Hypothetical SUD driving on an urban area (screening) dichotomized | | 0=not avoided (SUD <6)<br>1=avoided (SUD ≥6) |
| urban_sud.[1-3] | See descriptions for wheel_sud | | |
| urban_dic.[1-6] | See descriptions for wheel_dic | | |
| hypBAT_before | Hypothetical BAT before number of tasks mastered | | |
| actBAT_after | Actual BAT after number of tasks mastered | | |
| BAT_instructor_assessm | General assessment of driving performance during the BAT, as rated by the driving instructor | | 0=normal<br>1=slightly conspicuous<br>2=substantially conspicuous<br>3=severely conspicuous |
| BAT_instructor_interven | Number of interventions by the driving instructor during the BAT | | |
| BAT_Psych_gs1 | Number of driving errors regarding driving with higher speed than speed limit | | |
| BAT_Psych_gs2 | Number of driving errors regarding maladjusted speed (maladjusted acceleration or deceleration; offensive driving) | | |
| BAT_Psych_gs3 | Number of driving errors regarding driving too slow (unconfident driving) | | |
| BAT_Psych_ab12 | Number of driving errors regarding longitudinal distances being too small | | |
| BAT_Psych_ab3 | Number of driving errors regarding lateral distances being too small | | |
| BAT_Psych_fb1 | Number of driving errors regarding poor lane keeping | | |
| BAT_Psych_fb2 | Number of driving errors regarding lange changes | | |

| | | | |
|---|---|---|---|
| BAT_Psych_fb3 | Number of driving errors regarding driving on inappropriate lanes | | |
| BAT_Psych_fb4 | Number of driving errors regarding driving on inadmissible lanes | | |
| BAT_Psych_si1 | Number of driving errors regarding missing shoulder glances | | |
| BAT_Psych_si2 | Number of driving errors regarding right of way | | |
| BAT_Psych_si3 | Number of driving errors regarding overcautious shoulder glances | | |
| BAT_Psych_ko1 | Number of driving errors regarding signaling | | |
| BAT_Psych_ko2 | Number of driving errors regarding unclear communication | | |
| BAT_Psych_ko3 | Number of driving errors regarding interaction with other traffic participants' signaling | | |
| BAT_Psych_nav | Number of driving errors regarding navigation | | |
| BAT_Psych_gf1 | Number of driving errors regarding endangering VRU | | |
| BAT_Psych_gf2 | Number of driving errors regarding endangering motorized traffic participants | | |
| **Follow-Up: Evaluation** | | | |
| FU_Preference_Expo | Preference of Exposure after the treatment (follow-up) | | 0=in vivo<br>1=virtual<br>2=combination of both |
| FU_E_1 | The content of the treatment was comprehensible. | 1=do not agree at all<br>7=agree strongly<br>8=don't know | |
| FU_E_2 | The procedures of the treatment were comprehensible. | | |
| FU_E_8 | Driving in the simulation helped me overcome my fear. | | |
| FU_E_9 | I experienced driving in the simulation to be realistic. | | |
| FU_E_10 | Driving in the simulation made driving under real traffic conditions easier for me. | | |
| FU_E_11 | Treatment should be made accessible to broad public. | | |
| FU_E_12 | The difficulty of driving tasks was …. | 1=too easy<br>4=appropriate<br>7=too difficult<br>8=don't know | |
| FU_E_13 | The speed of the proceedings was… | 1=too slow<br>4=appropriate<br>7=too fast<br>8=don't know | |

| | | | |
|---|---|---|---|
| FU_E_15 | The time frame was… | 1=too short<br>4=appropriate<br>7=too long<br>8=don't know | |
| FU_E_19 | Overall evaluation of the treatment | German school grade:<br>1=very good<br>6=fail | |
