# Supplementary material for: Treating patients with driving phobia by virtual reality exposure therapy – a pilot study
Source: PLoS One. 2020 Jan 7;15(1):e0226937. doi: 10.1371/journal.pone.0226937 (PMC6946146; doi:10.1371/journal.pone.0226937)
Supplement: S1 Protocol — (PDF) [file pone.0226937.s004.pdf]

Study Protocol

Treatment of driving fear caused by traffic accidents – development and evaluation of exposure therapy in the driving simulation.

- a pilot study

Dr. Yvonne Kaussner[1]
Dr. Ramona Kenntner-Mabiala[1]
Dr. Nora Walz[2]
Dr. Petra Markel[2]
Prof. Paul Pauli[2]

[1]WIVW GmbH
Würzburger Institut für Verkehrswissenschaften
Robert-Bosch-Straße 4
97209 Veitshöchheim

[2]Hochschulambulanz für Psychotherapie
Institut für Psychologie
Universität Würzburg
Marcusstraße 9-11
97070 Würzburg

Amended Version 24.09.2015

1 PROBLEM STATEMENT

Each year about 2.500.000 traffic accidents happen in Germany. Approximately 300.000 of these accidents include damage to persons, with about 400.000 persons being in an accident (i.e. minor to severe injuries, fatalities; Statistisches Bundesamt, 2014).

Following an accident, up to 50% of the involved persons suffer from related stress symptoms, and approximately 8% develop a posttraumatic stress disorder (PTSD). This means that approximately 1 out of 12 persons who were involved in a medium severe to severe traffic accident will develop a posttraumatic stress disorder which needs to be treated (Maercker, 2003). Additional psychopathologies, such as acute stress reactions, adjustment disorders, or specific (isolated) phobias, resulting in driving fear subsequent to traffic accidents, may occur as well, so that it may be reckoned with a higher psychological morbidity rate of up to 30% (Nyberg et al., 2003). The resulting driving fear can be specific, for example driving on highways or unfamiliar routes, or general. Thoughts circle around catastrophes in road traffic or worries to cause an accident or injure others. When the persons concerned try to drive despite the fear of driving, they react with severe psychological symptoms, which result in avoiding these situations and driving in general.

For professional drivers the driving fear can result in a reduction in earning capacity or even occupational disability. But even if a person is not a professional driver the driving fear can impair occupational rehabilitation, as it might be impossible to reach the place of work by car.

Cognitive behavioural therapy, especially exposure therapy, is regarded as the method of choice when it comes to treating anxiety disorders. Increasingly, virtual methods are used (Mühlberger & Pauli, 2011). There are only occasional experiences and studies regarding the effectiveness of therapeutic exposure in driving simulation. These, however, can be seen as very promising (e.g. Beck et al., 2007). In particular, this is shown in the investigation of individual cases that have been and still are conducted with professional drivers in the driving simulator of the Institute for Work and Health of the German Social Accident Insurance (IAG). It is noticeable, however, that the intervention is very efficient for some, whereas others do not experience any fear in the driving simulation and thus do not have therapeutic success. According to the IAG it is not possible to systematically investigate the factors leading to successful exposure in the driving simulation during daily cooperation with the clinics of the workers' compensation board. For this reason the IAG approached the Würzburg Institute for Traffic Sciences (WIVW), being the researching supplier of the IAG driving simulator, with the explicit request for a systematic investigation. The solution to cooperate with the psychotherapeutic outpatient clinic of the University of Wuerzburg suggested itself due to the expertise in research and treatment of anxiety disorders and virtual exposure therapy (see Focus Ärzteliste 2013).

Many patients refuse or discontinue a classic therapy with in vivo exposure due to strong fear. A preceding virtual exposure might help as patients are cognitively aware of not being in real danger. For example, Garcia-Palacios et al. (2007) report that 27% of the examined patients suffering from a specific phobia refuse an in vivo exposure, whereas only 3% refuse a virtual exposure. When asked to choose, 76% of the patients favoured virtual instead of in vivo exposure. The advantage of a (preparatory) exposure in the driving simulator over mere in

vivo exposure therefore lies in the approach to exposure having a lower threshold. Exposure is the key therapy module in the treatment of anxiety.

Further advantages are offered, such as:

- Therapeutic control: Exposure can be interrupted immediately and at any time
- Situational control: The relevant situations can be realized systematically and presented in hierarchical order; it is not necessary to actively search for them under real driving conditions or to wait until they occur coincidentally (e.g. overtaking manoeuvre of an oncoming vehicle, passing an accident, emergency vehicles, rain). Moreover, no unexpected or unwanted situations happen in virtual exposure. This cannot be controlled in vivo.
- Evoking memories of circumstances / trigger in the course of therapy which were not conscious previously and can be implemented in the simulation
- No real endangering (not only being relevant for safety, but also having a high cognitive importance for patients).

Thus, in the project proposal at hand a pilot study is presented in which an exposure therapy in the driving simulation is to be developed and evaluated with a small, thoroughly selected sample of patients. Provided that the results are positive, subsequently a larger evaluation study will be conducted in order to identify predictive criteria for the success of such therapy and to retrieve the optimal design. In the long term it is intended to implement the developed virtual exposure into therapeutic practice.

2 CURRENT STATE OF KNOWLEDGE

2.1 Proof of a performed systematic literature research, description of the retrieval strategy

A systematic literature research was conducted to gain comprehensive insight in the current state of research regarding the therapy of driving fear. The database (Datenbank-Infosystem – DBIS) form the University of Würzburg, with access to 72 databases, was used to conduct the research. The databases PsycINFO, PSYNDEX and PsychTHERAPY from the “Psychology” department were used. The following key words in all possible combinations were used for the search:

- Virtual reality
- Exposure therapy
- Efficacy virtual exposure therapy
- Anxiety disorders
- PTSD (posttraumatic stress disorder)
- Driving phobia
- Fear of driving
- Road accident
- Motor vehicle / traffic accident.

Systematic reviews, meta-analysis, single case studies, quasi- and randomized experimental studies were included.

2.2 Summary of relevant Studies (own and external)

Virtual reality (VR) is increasingly being used in psychotherapy. Exposure therapy in virtual reality (VRET) is of particular importance in the treatment of anxiety disorders, as it shows broad evidence (Powers & Emmelkamp, 2008). In contrast to exposure in vivo or in sensu, the fear-inducing stimuli or situations are simulated by a computer in VRET (Rothbaum, 2009). Patients clearly prefer VRET over in vivo exposure (Garcia-Palacios et al., 2007). The clear advantage of VRET regarding in vivo exposure is the low-threshold approach, lower drop-out rates and a higher willingness to therapy of the patients. Another advantage shows in cases where the stimuli or situations are hard to recreate or hard to find, but need to be controlled. Situations where certain psychological or somatic symptoms could potentially produce a safety risk, as it is the case in driving, can be created.

VR is being used in the treatment of anxiety disorders and PTBS due to the fact that those disorders often come with a clearly defined anxiety inducing stimulus or situation (e.g. spider, battleground) which can be simulated. The research regarding the treatment of fear of flying conducted by the applicant (Lehrstuhl für Psychologie I der Universität Würzburg) shows great evidence for the use of VRET. The studies conducted by the applicant show that various flights in VR (Mühlberger et al., 2001), as well as solely one VR-flight (Mühlberger et al., 2003) can reduce the fear of flying significantly. The effectiveness of VRET treatment of arachnophobia is well established (Garcia-Palacios et al., 2002). Studies comparing in vivo exposure with VRET show similar results in effectiveness for both forms of treatment (e.g. Emmelkamp et al., 2002; Rothbaum et al., 2000; McLay et al., 2010; Michaliszyn et al., 2010).

Traffic accident victims that develop a fear of driving clearly show a change in driving behavior. They either drive overly cautious, interfering with other road users, or stop driving completely (Maercker, 2003). Stimuli reminding of the traffic accident are being avoided, as they produce a strong physical anxiety reaction. However, a strong excitation is necessary to induce a correction of the anxiety structure (Foa & Kozak, 1986; McNally, 2007). After extensive preparation (medical anamnesis, disorder model, fear hierarchy, rational of change) the patient is repeatedly confronted with the fear inducing stimulus without the possibility of avoidance. One has to pay attention to subtly used avoidance behavior (e.g. cognitive distraction), as complete avoidance of certain situations is not always the case. But to reach cognitive reorganisation complete exposure to fear and the fear inducing stimulus is necessary. The patient habituates to the fear symptoms through repeated and sufficiently long exposure. Beck et al. (2007) could show that a treatment with VRET in a high-fidelity driving simulator of 6 patients suffering from PTSD after a traffic accident lead to a reduction of PTSD-symptoms. The treatment consisted of 10 sessions, of which the first two were used as preparation for the exposure. Elements of which were information about PTSD, relaxation techniques and creating a fear hierarchy. The 8 following sessions consisted of exposure. The study showed that symptoms consisting of reliving, avoidance tendencies and emotional mental dullness were significantly reduced pre- to posttreatment (ES: $d = .79 – d = 1.49$). The authors could also show that this improvement could not solely be accredited to a decline in depression or other anxiety symptoms. The patients were highly satisfied with this approach.

The effectiveness of VRET could also be established for patients with driving fear. A single case by Wald and Taylor (2000) focused on treating a person with driving fear in a time span of 10 days with three therapy sessions through the use of VRET in a static driving simulator.

The patient had to drive through four different scenarios with increasing difficulty. The driving simulator consisted of a computer display with a steering wheel and acceleration pedal. The driving environment was presented through a head-mounted display. Once the patient reached a rating of 10 or lower on the anxiety scale reaching from 0-100, the next scenario was used. The patient showed a reduction of fear symptoms and avoidance tendency after three treatments. The success of the treatment was stable at the seven months after treatment follow-up.

Wald (2004) investigated the effectiveness of VRET for five patients with driving fear. Three of five patients showed an improvement of driving fear and less avoidance tendencies between pre- and posttreatment. For one patient the treatment lead to only a marginal improvement of the symptoms and the last patient did not profit from the treatment. The use of VRET could not lead to an increase in driving for all five patients. On the basis of the evidence regarding the effectiveness of VRET for driving fear (Wald & Taylor, 2000; Wald, 2004) it is suggested to use VRET as a preparative treatment for in vivo exposure for driving fear and not as sole treatment (Wald & Taylor, 2003).

2.3 Deficit analysis

The presented studies indicate that driving fear after a traffic accident can be treated with VRET. However, most of the studies are single case studies or non-randomized case studies (without control group) with small sample sizes, preventing a final statement of the specific effect of VRET. The study by Beck at al. (2007) investigated the effect of VRET-treatment on specific symptoms of PTSD but not on the driving behavior. Further, the few publicized studies and the experience of IAG show that some people profit greatly from VRET, while others do not profit at all. No differentiating or predictive characteristics are known yet. Further studies with a larger sample size, a controlled randomized design, as well as dependent variables on behavioral, subjective and physiological level are necessary to answer a number of questions regarding research and application:

- Which patient characteristics are predictors for therapeutic success? (e.g. characteristics of the accident, previous therapy experiences)
- How effective is a (preparative) VRET in a driving simulator compared to sole in vivo exposure?
- Which design characteristics of the driving simulator sessions increase therapy success (e.g. Level of individualization of the used scenarios or expansion stage of the driving simulation)?
- How does VRET change driving behavior?
- How far can VRET in driving simulation improve symptoms such as depression, anxiety and flashbacks?

3 OBJECTIVE AND TARGET GROUP

Patients

The planned pilot study should show that phobic avoidance behavior after a traffic accident can be reduced through confrontation- and practice sessions in virtual reality in a driving simulator. If effectiveness of this new therapy element can be proven a follow-up project will

concentrate on the therapeutic design and further research questions should be answered (see 2.3). Long-term this method of treatment should be implemented into psychotherapeutic treatment.

The target group therefore consists of persons that were involved in a traffic accident as the driver of a motorized vehicle and subsequently developed a fear of driving with avoidance of driving. Possible clinical-psychiatric diagnoses could be “adaption disorder”, “specific (isolated) phobia” or PTSD. The pilot study at first only looks at professional drivers that were reported to the BG Verkehr (German insurance association for transport and traffic) with the aforementioned symptoms. A detailed list of the inclusion and exclusion criteria can be found in section 5.1.1

Operators

The insurance associations (BGen) and their trauma-guides, which arrange the implementation of such exposure therapies, can be seen as the target group, besides the patients themselves. This study specifically mentions the BG for transport and traffic but the produced measure should be accessible to all BGen (insurance associations).

4 RELEVANCY FOR THE GERMAN SOCIAL ACCIDENT INSURANCE (DGUV)

Estimations made by trauma-guides of the traffic trade associations show that approximately 175 motorists a year need to be treated either outpatient or inpatient for driving fear following a traffic accident (see section 5.1.1). If the fear of driving is left untreated it can lead to a reduction in earning capacity (Minderung der Erwerbsfähigkeit, MdE) or even to occupational disability. If the reduction of earning capacity is at least 20% (SGB VII) the insured person receives a pension from the trade association. Efficient therapy of the disorder could lead to a quick resumption of occupational activity or even restoration of full earning capacity and the prevention of subsequent costs. The effectiveness of a short-term-psychotherapy of driving fear with a new module “exposure in driving simulation” should be demonstrated within the study described in the following sections. If the proof is successful, the treatment should be implemented as a one-week-block-therapy. It is time-efficient and can reduce treatment cost compared to the standard method. The exposure should be performed in the driving simulator of WIVW with the software SILAB. The exposure-module would be executable on the driving simulators of IAG and DGUV, as they use the same software. This offers a new field of application for the therapy of driving fear at the local simulators.

5 METHODS

The investigation is designed as a pilot study (in terms of a proof of concept) and is to be conducted in a research cooperation between the WIVW and the psychotherapeutic outpatient clinic of the University of Wuerzburg.

The aim of the study is to investigate the effectiveness of exposure therapy in the driving simulation. For this reason, a small sample of 2 x 10 patients with driving fear (treatment and waiting group) should be included in a week-long block therapy. The study does not aim at investigating a new method of therapy, but solely at testing the effectiveness of a new element of therapy, i. e. VRET in the driving simulation. The VRET will not be conducted separately, but will be prepared and followed-up psychotherapeutically. It is to be analysed if driving fear, coming along with strong avoidance behaviour, can be reduced for patients with the diagnoses „adjustment disorder“, „specific (isolated) phobia“ or „post-traumatic stress disorder“ in a way, that they can return to driving under real driving conditions with adequate driving behaviour. Additionally it will be investigated if symptoms of depression and anxiety as well as difficulties in concentrating may be reduced by the virtual exposure therapy. For this purpose different subjective, physiological and behavioural outcome parameters are examined. However, the primary criterion of treatment success for the VRET in the driving simulation will be the driving performance during the final driving test under real driving conditions, which will be accompanied by a driving instructor and will be completed subsequently to the VRET (and will at least be offered prior to the VRET for checking purposes). Driving performance will be evaluated by the driving instructor and a traffic psychologist, who will be blinded with regard to group membership. The waiting group is supposed to control for time effects, spontaneous remissions and potential therapeutic effects of study-related but treatment independent activities (medical consultation, psychotherapeutic anamnesis, contact with driving instructor / traffic psychologist). Only by comparing with a waiting group and by blinding with respect to the group membership a bias concerning the evaluation of the driving behaviour can be controlled (e. g. a generally milder judgement in the second driving test subsequent to therapy, which would be confounded with a positive effect of therapy).

If the effectiveness can be proven by preliminarily defined criterions, a follow-up study will be applied for. The follow-up study should include an evaluation of a larger, power-calculated sample and answer further exploratory and practice-oriented questions (e. g. identification of characteristics of patients that can predict therapeutic success, or minimum requirements for configuration stages of driving simulators). The aim of the follow-up project would thus be the most efficient and economic future implementation into therapeutic practice.

5.1 Methodological procedures

5.1.1 Recruitment of patients and definition of inclusion and exclusion criteria

The sample, which will only consist of patients, will be recruited throughout Germany with help of the district administrations of German insurance association for transport and traffic (BG Verkehr).

Assuming availability of all personnel and technical resources, it is expected that each month two week-long block therapies can take place. Thus, it can be assumed that the sample with the intended size of 2 x 10 patients can be investigated within one year. According to the trauma unit of the district administration in Dresden, each year about 100 motorists with mental disorders caused by work accidents are registered, with one quarter of them needing inpatient or at least ambulant psychotherapy. Assuming comparable numbers for the remaining district administrations, from a group of approximately 175 patients could be recruited within one year. The potential for recruitment indicates that it is realistic to conduct the study within one year.

After a patient’s registration at the worker’s compensation board the patient will be informed about the possibility of participating in the study. After having received the permission, the

staff of the psychotherapeutic outpatient clinic of the University of Wuerzburg will telephone the patients four weeks after the accident at the earliest in order to inform the patient about the study in more detail. If the patient is still interested in participating, a first telephonic inquiry of the inclusion and exclusion criteria as well as a first diagnosis according to the ICD-10 will follow. In order to replicate the results of Garcia-Palacios et al. (2007), showing that patients prefer VRET over in-vivo exposure, the patients will be asked during the screening call which type of exposure they would prefer. Based on relevant literature and the experiences of the IAG with the individual cases addressed at the beginning, only inclusion and exclusion criteria will be chosen that will – according to the current state of knowledge – allow to expect a high effectiveness of exposure.

Inclusion criteria:

- Professional drivers with “adjustment disorder”, “specific (isolated) phobia” or “post-traumatic stress disorder” as a consequence of a work / traffic accident with a motorised vehicle, the accident having been a frontal / lateral collision, and with the patient having been the driver (precondition for visual hazard cues)
- coming along with driving fear / driving avoidance
- for at least four weeks
- no prior exposure therapy
- no or stable therapy with psychoactive drugs for at least four weeks
- valid driving license and regular driving experience prior to the accident
- aged 18-63 years

Exclusion criteria:

- alcohol / drug addiction
- suicidal tendency
- Psychosis and other premorbid mental disorders and comorbidities such as coronary heart disease and epilepsy as well as other findings rated to be a contraindication to an exposure therapy during the investigation at the Medical Study Center Wuerzburg
- severe cognitive impairment (score < 27 in the Mini Mental Status Test, Folstein et al., 1975)
- For females only: positive pregnancy test

If all inclusion criteria and none of the exclusion criteria are met in the first assessment, the patients will be randomly assigned to one of the two experimental groups (treatment vs. waiting group) and will be invited for an extensive examination and treatment in Wuerzburg.

In the first instance, the avoidance of driving will be subjectively inquired by hypothetically offering a driving test. Immediately prior to the therapy the avoidance behaviour will be behaviourally verified by offering a real driving test. The driving test with driving instructor which will be offered at the beginning of the treatment should be rejected or only be completed with very strong anxiety and conspicuous driving behaviour[1] (so-called Behavioural Avoidance Text, BAT).

The randomisation will be decided by drawing lots. Each patient contacted by the psychotherapeutic outpatient clinic of the University of Wuerzburg will initially get a consecutive number, beginning with 1001. Each person that meets the inclusion criteria

[1] For an operationalisation of “conspicuous driving behaviour” see Chapter **Fehler! Verweisquelle konnte nicht gefunden werden.**.

according to the telephonic assessment of the psychotherapist and agrees to participate in the study will get a consecutive randomisation number, beginning with 101. The assignment to one of the two groups will be decided by drawing lots. For this purpose, ten lots labelled “treatment group” and ten lots labelled “waiting group” will be sealed and put into a box. At the end of the screening call and after assigning the randomisation number the psychotherapist will draw a lot in order to be able to make the appointments (which will differ between the two groups) with the patient. Screening number, randomisation number, and group assignment will be documented in a list. Dropouts will be replaced by adding another lot to the box, labelled with the particular group.

5.1.2 Treatment procedures

The procedures for the week-long block therapy are described separately for the treatment and waiting group (see Table 1). The group assignment of the patients will be by chance (see previous chapter).

After the screening call the treatment will start for both groups on a Monday morning. The psychotherapist will welcome the patient at the psychotherapeutic outpatient clinic. The patients once again will get detailed information about the study in person and will be asked to give their informed consent for the participation in written form (Informed Consent). Also, it will be made sure that the diagnosis “adjustment disorder”, “specific (isolated) phobia” or “post-traumatic stress disorder” as according to ICD-10 really applies to the patient. Then, the patients will be physically examined by the investigator of the Medical Study Center Würzburg, which will be subcontractor for this task. During the medical-neurological screening examination the investigator will check potential contraindications for exposure. This means an extensive medical anamnesis including psychiatric findings and a screening for psychiatric diseases (including suicidal tendency, addiction), a physical (internal-neurological) examination, ECG, EEG, and a drug screening (plus a pregnancy test for females) will be performed. For ruling out severe cognitive impairments the MMSE (Folstein et al., 1975) will be used.

When all inclusion criteria have been verified and none of the exclusion criteria are met, the patient will be referred back to the psychotherapist of the psychotherapeutic outpatient clinic of the University of Wuerzburg. The treatment can start immediately afterwards. The psychotherapist will adhere to a therapy manual, which will have been prepared on the basis of relevant manuals according to literature (Hamm, 2006, König, 2012, Zöllner et al., 2005; among others; also see chapter 5.5).

The treatment will start with a psychotherapeutic anamnesis interview. The contents will be as follows:

- Rough recording of the event of the accident (with help of the Motor Vehicle Accident Interview, Blanchard & Hickling, 2004)
- Collection of symptoms, avoided cognitions and particularly critical driving situations (“hot spots”)
- Repeated explanation of the treatment procedures
- Different questionnaires regarding anxiety and depression
- Recording of difficulties in concentrating (d2-test, Brickenkamp et al., 2010)

Additionally, as indication for operant factors, the criteria therapy motivation, workplace stress and psychosocial stressors are to be measured with help of the following standardized methods during anamnesis[2]:

- AVEM (work-related behaviour and experience patterns; Schaarschmidt & Fischer, 2003)
- FPTM (Fragebogen zur Psychotherapiemotivation; Nübling & Schulz, 2002)
- Social Readjustment Rating Scale (Holmes & Rahe, 1967)
- ABF (Daily Stress Inventory; Alltagsbelastungsfragebogen; Traue et al., 2000)

Table 1: Treatment procedures for patients in the treatment group (TG) and the waiting group (WG). PT-session = psychotherapeutic session.

	Day-x	Day1: Mon	Day2: Tue	Day3: Wen	Day4: Thu	Day5: Fr	Day8: Mon	Day9-12: Tue-Fr	+1.5/3 months
TG	Screening call	Physical examination<br>Psychoth. anamnesis BAT with driving instructor	PT-session with startle response measurement	PT-session	Simulator: Exposure 2 (medium hierarchy level)	Simulator: Exposure 5 (repetition of scenarios from medium and upper level)			Booster / Follow-up call
			Familiarizing with the simulator	Simulator: Exposure 1 (lower hierarchy level)	Simulator: Exposure 3 (upper hierarchy level)	BAT with/ without driving instructor<br>Psycho-therapeutic closing session with startle response measurement			Booster / Follow-up call
WG	Screening call	Physical examination						see TG	Booster / Follow-up call
		Psychoth. anamnesis Hypothetical BAT offer with driving instructor					BAT with driving instructor	see TG	

Subsequent to the anamnesis interview the patients of the treatment group will be offered to drive a short distance in a driving school vehicle, accompanied by the psychotherapist, a driving instructor, and a traffic psychologist of the WIVW.

As already described, patients will only be included in the study if they reject the driving test (Behaviour Avoidance Test, BAT), or if they complete the driving test with very conspicuous and anxious driving behaviour (according to the driving instructor and the traffic psychologist). The patients of the waiting group, which is serving as a control group, will have to wait one week from the anamnesis interview for the treatment to be continued. The waiting group will only be hypothetically offered the driving test. For this purpose, the psychotherapist will ask the following questions:

1. If we would now offer you to drive a short distance, accompanied by a psychotherapist and driving instructor, would you do this? Yes – No

[2] If this cannot be fulfilled during anamnesis because there is not enough time or because it would lead to too much stress for the patient, these questionnaires can also be given on day 2 (treatment group) or day 9 (waiting group).

2. If yes, how stressful would this be for you? Answer on 16-stage numeral scale with the verbal superordinate categories (not at all – very little – little – medium – much – very much)

Included will only be

- patients who answer “no” in the first question or
- patients who answer “yes” in the first question but give at least “much” as an answer in the second question

The patients of the waiting group will be re-invited for the coming week in order to receive the treatment as well. The ensuing Monday they will, once again, be offered the driving test. In the case of agreeing to do it they would really have to drive (corresponding the treatment group). Likewise, the questionnaires of the anamnesis will be given again. Only if the BAT is rejected or completed with very conspicuous and anxious driving behaviour, the VRET treatment will, equivalent to the treatment group, begin on Tuesday morning.

In order to counteract any worsening of the symptoms after the BAT or general crisis situations during the study, the psychotherapist will be available for a therapeutic session after the BAT. Furthermore, all patients will be handed out an emergency number for being able to call the psychotherapist of the psychotherapeutic outpatient clinic.

The treatment itself will begin with a psychotherapeutic session on Tuesday morning. The contents will be as follows:

- presentation of the explanatory model regarding anxiety disorder and development of an avoidance hierarchy for driving scenarios
- illustration of the treatment rationale (explanation how an exposure can help overcome anxiety)
- writing an accident report in presence of the therapist

The degree of driving anxiety will be measured by the so-called startle response during this session. This is a contraction of muscles at the eyelid, which is measured by electromyography at the musculus orbicularis oculi. The response is triggered by six uncomfortable auditory stimuli in a neutral situation and during a reading exposure with the self-written accident report. It is assumed that the response will be stronger during the reading exposure than in the neutral situation and that the difference between the two situations will be smaller after the therapy than prior to it. Moreover, patients will be asked to assess the degree of their anxiety in both situations (by using the 10-stage Subjective-Unit-of-Distress-Skala; for giving the so-called “SUD-ratings”).

Subsequent to the psychotherapeutic session the psychotherapist will discuss the developed hierarchy of driving scenarios with a traffic psychologist and a scenario developer of the WIVW, so that the relevant scenarios can be programmed for the driving simulation. It is the aim to hierarchically structure the short scenarios individually for each patient so that the anxiety prior to driving through a scenario will increase with hierarchy level and climax in the most avoided scenario. This will be ensured by presenting more and more so-called “hot spots” (fear-inducing stimuli), which have been identified in the therapeutic interview (e.g. crossroads with cross traffic, crossroads with emergency vehicles in cross traffic, emergency vehicles in parallel traffic, passing an accident scene with emergency vehicles). Where applicable, the exposure scenarios used by IAG, which had been programmed on the basis of the initially reported individual sessions, might be used, as both the IAG and the WIVW simulator, which will be used in the present study, are operated by the SILAB software.

On Tuesday afternoon the patients can visit and get acquainted with the driving simulator (see Figure 1). They can sit in the driving simulator and – if they want to – drive on a parking lot with few stimuli and an easy country road. The patients will deliberately not be exposed to the driving scenarios of the developed anxiety hierarchy.

Prior to, during, and after visiting the driving simulator the patients will be asked to rate their perceived anxiety with help of the SUD scale. Moreover, the psychotherapist will record the patients' avoidance behaviour by using partially standardised observation sheets. If a patient agrees to drive, the driving behaviour will be rated by a trained traffic psychologist. For this purpose, the psychologist will use an application for tablet pcs that has been developed by the WIVW for rating driving behaviour (Standardized Application for Fitness to Drive Evaluations S.A.F.E., see Figure 1). From experience, even healthy test persons initially often drive very slowly and carefully in the driving simulation. This has to be taken into account for the rating. At the IAG, however, extremely conspicuous behaviours were observed during sessions with PTSD patients (e.g. maximum speeds of less than 10 km/h). This will have to be recorded. As particularly the first drives in the simulation might be accompanied by sickness, "simulator sickness" will be controlled by a questionnaire regarding physical discomfort (based on Kennedy et al., 1993).

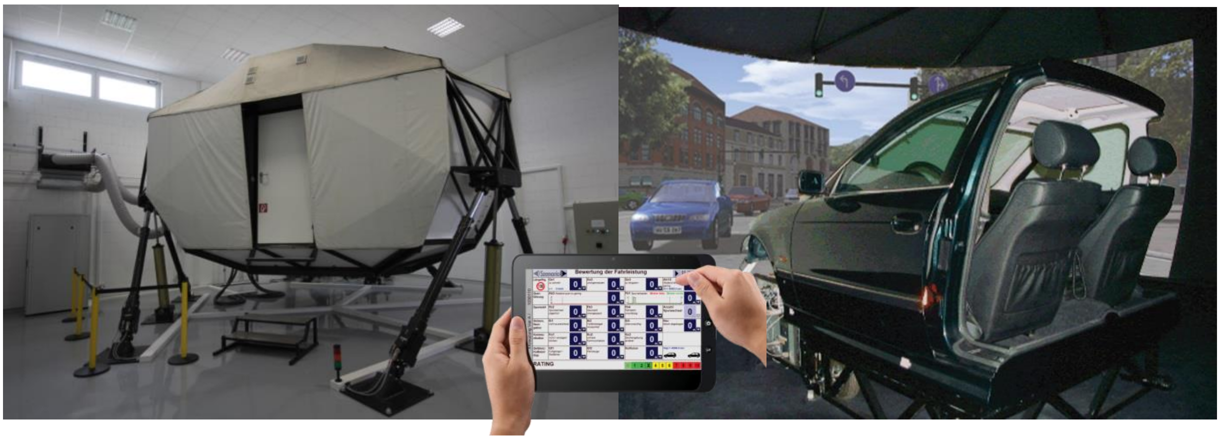

Figure 1: The dynamic driving simulator of the Würzburg Institute for Traffic Sciences which will be used for exposure therapy. The integrated vehicle's console contains all the necessary instrumentation and is identical with a production type BMW 520i with automatic transmission (right) which is situated in a dome. Three LCD projectors are installed in the dome of the simulator and provide the projection. Three channels provide a 180° screen image. The exterior and interior mirrors function as LCD displays. The psychotherapist will be in the nearby operator room and take care of the patient via microphone. The psychotherapist will be able to see the driver, the current driving scenario, and real-time driving data via different monitors. A trained traffic psychologist will record driving errors and conspicuous driving behavior with help of a partly automatized application for tablet-pcs (Standardized Application for Fitness to Drive Evaluations, S.A.F.E.).

Wednesday morning will begin with a psychotherapeutic session in which the visit of the driving simulator will be discussed and the patient will be prepared for exposure in the simulation that will take place in the afternoon. In the framework of three exposure sessions it is the aim to successively drive through the hierarchical order of the driving scenarios as it has been developed with the therapist and implemented into the simulation. Ideally, it will be the lower hierarchy level on Wednesday afternoon, the medium hierarchy level on Thursday morning, and the upper hierarchy level on Thursday afternoon. However, spontaneous changes that are individually adapted to the patient and seen as reasonable by the therapist may and should be considered and implemented. When programming the scenarios a slow

habituation to driving in the simulation should be taken into account by gradually increasing the necessity of manoeuvres such as braking, accelerating and steering. This kind of simulator habituation is standard procedure at the WIVW. It serves the development of a natural driving behaviour and reduces the risk of simulator sickness considerably (see Hoffmann & Buld, 2006).

Prior to each exposure session the traffic psychologist will present the scenarios that have been planned and programmed in the meantime to the psychotherapist. The exact course of action and the wording of the instruction prior to and during the drive will be discussed. Smaller adaptations will still be possible. During the exposure session the subjectively perceived anxiety, the avoidance and driving behaviour as well as symptoms of simulator sickness will be recorded. Figure 2 shows exemplary scenarios that might be thinkable for the lower and upper hierarchy level.

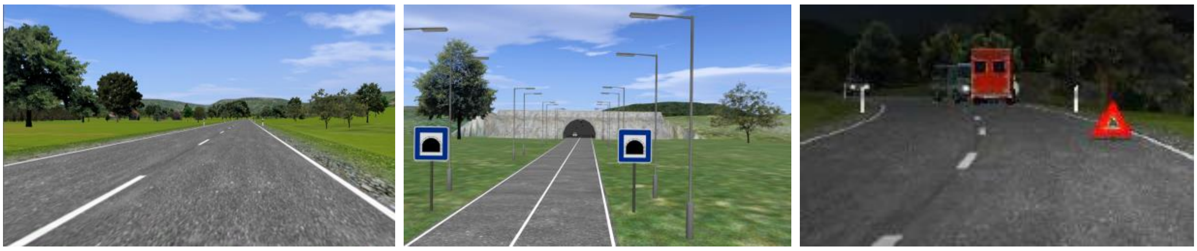

Figure 2: Exemplary scenarios of the software SILAB for the lower (left: continuing along an easy country road) and upper (centre: driving through a tunnel; right: passing a scene of accident with emergency vehicles at night) individual hierarchy level during exposure in the driving simulation.

During the last exposure session on Friday morning the effectiveness of the VRET treatment will be validated for the individual patient by repeating one simulation scenario from the medium and one from the upper hierarchy level. Again, the subjectively perceived anxiety (SUD ratings), the avoidance and driving behaviour as well as symptoms of simulator sickness will be recorded.

During all exposure sessions in the simulation the heart rate response will be recorded as objective indicator of psychological stress. This means that the heart rate of the beginning of an exposure will be compared to a baseline heart rate (after exposure, during a short relaxation, instructed by the psychotherapist). The baseline-corrected change between and within sessions will be used for analysis.

In the psychotherapeutic closing session on Friday afternoon the contents of therapy will be summarized, potential daily stress factors which might impede maintenance of treatment success will be determined, and means for relapse prevention as well as a training plan for the upcoming weeks will be developed. Additionally, the necessity of further therapy sessions and therapy contents with an outpatient therapist at home will be pointed out. As part of the closing session the d2-test (Brickenkamp et al., 2010) will be repeated with regard to a potential decrease of concentration difficulties. The same applies for the repeated measurement of the startle response during a neutral situation and a reading exposure situation (own accident report), in order to assess the effectiveness of therapy via this physiological indicator. SUD-ratings, too, will be recorded once again as a subjective measurement.

The primary outcome parameter for assessing the success of therapy will be, however, if a driving test accompanied by driving instructor, psychotherapist, and traffic psychologist will or will not be completed with adequate driving behaviour and reduced fear after the therapy. There will be various layers of the evaluation: driving instructor and traffic psychologist will rate the driving behaviour (by S.A.F.E.), the psychotherapist will rate the avoidance behaviour

(partly standardized observation sheet) and the patients will subjectively scale their fear themselves (SUD-rating). If the driving test is successful, patients will finally be asked to shortly drive an experimental vehicle of the WIVW unaccompanied in order to rule out that the success has to be attributed to the presence of driving instructor and psychologists. If the BAT still causes strong psychological stress, the psychotherapist will be available for a therapeutic follow-up afterwards.

After the week-long treatment patients will be referred to an outpatient therapist near their home by the worker's compensation board (if this has not already happened). However, six weeks after finishing the treatment a "booster session" by phone with the psychotherapist of the study will be planned in order to ask the patients about difficult situations and how they handled them as wells as to refresh the most important therapeutic insights. The study closure will be a follow-up examination three months after having finished the treatment. Different standardized and relevant questionnaires with regard to anxiety and depression, which have already been submitted during anamnesis, will be repeated. Moreover, a diagnostic interview will measure how distinct driving fear and the associated avoidance behaviour still are. Finally, the patient will be asked to evaluate the treatment overall.

5.1.3 Outcome measures to evaluate effectiveness

As shown in the study procedures, in the course of the study numerous outcome measures will be recorded in order to evaluate the effectiveness of the treatment on subjective, psychological, and behavioural level. For a better overview these (plus the information when exactly and by what kind of methods they will be collected) shall be listed here once again in detail:

Primary outcome:

- Driving performance and behaviour in the driving test under real driving conditions after and, if applicable, prior to the treatment. This will be rated by the driving instructor and the traffic psychologist using S.A.F.E. (operationalisation and dealing with inconsistencies see chapter 5.1.4).

Secondary outcomes:

- Avoidance behaviour during the driving test under real driving conditions prior to and after the treatment by partly standardized observation sheet of the psychotherapeutic outpatient clinic
- Subjective fear during the driving test prior to (if patient drives despite the driving fear) and after the treatment as well as in the course of the simulator sessions by SUD-scale
- Startle response (mean reaction to six acoustic stimuli) by EMG during a neutral situation and a reading exposure (own accident report) prior to and after the treatment
- Power of concentration by d2-test (Brickenkamp et al., 2010) prior to and after the treatment
- Baseline-corrected change of the heart rate between and within the exposure sessions
- Accident fear prior to treatment and at follow-up via „Accident Fear Questionnaire, AFQ“ (Kuch et al., 1995)
- "Posttraumatic Stress Scale Self-Report PSS-SR" (Foa et al., 1997) prior to treatment and at follow-up

- Symptoms of general anxiety and depression prior to treatment and at follow-up via “Beck Anxiety & Depression Inventory“ (Beck, Steer, & Brown, 1996; Beck & Steer, 1993)
- Driving and avoidance behaviour in the simulation during the simulator sessions via S.A.F.E. rating by the traffic psychologist and therapist rating with help of partly standardized observation sheet
- Simulator sickness prior to and after each drive in the simulation via list of symptoms based on Kennedy et al. (1993)

5.1.4 Operationalisation of the outcome measure driving performance and definition of responder vs. non-responder

Based on the BAT ratings of the driving instructor and the traffic psychologist on day 5 (treatment group) or day 8 resp. (waiting group), all patients will be divided into responders and non-responders. The rating of the driving instructor will be operationalised on a scale with 4 categories, as described by Brenner-Hartmann (2002) for standardized driving behaviour observation in the framework of medical-psychological examinations.

According to this, at the end of the drive, the driving instructor rates his or her overall impression on a scale with four categories: “not conspicuous – slightly conspicuous – substantially conspicuous – severely conspicuous”. Corresponding to Brenner-Hartmann (2002) the transition from slightly conspicuous to severely conspicuous will be the cut for adequate driving behaviour. The rating of the traffic psychologist (according to the project description) certifies an adequate driving behaviour if it is not higher than 3 on the 10-level Fitness-to-Drive scale, which is based on Neukum & Krüger (2003; see Figure 3), at the end of the drive. As the allocation concealment of group membership is easier to realise for the driving instructor[3], his or her rating shall have priority in the case of inconsistencies.

Inconsistencies shall be handled the following:

- If the driving instructor rates the driving behaviour to be substantially or severely conspicuous, the performance will be seen as inappropriate, regardless of the traffic psychologist’s rating.
- If the driving instructor rates the driving behaviour to be slightly conspicuous at the max, and the psychologist rates it to be critical (values 7-10 on the FtD-scale), the performance will be seen as inadequate.
- If the driving instructor rates the driving behaviour to be slightly conspicuous at the max, the driving behaviour will be seen as adequate even if the psychologist rates it to be conspicuous (values 4-6 on the FtD-scale).

[3] The therapy will take place at the WIVW. For this reason, the traffic psychologist will meet patients of the treatment group with a higher likelihood than the patients of the waiting group. Potential contact between patients and WIVW employees will be minimized. It will be guaranteed that the psychologist who will be rating the driving behaviour test will not have been involved in the therapy sessions and will not be informed in detail about the study procedures (particularly not about the fact that the driving test at the end of the treatment and waiting phase will take place on different weekdays). Of course the driving instructor will not be informed about the different scheduling either. In order to maintain the allocation concealment patients will be asked to talk as little as possible during the driving test.

Verbal category	Numerical subcategory
Absolutely unfit to drive	10
Critical driving behavior	9
	8
	7
Noticeable driving behavior	6
	5
	4
Normal driving behavior	3
	2
	1
Fit to drive without any restrictions	0

Figure 3: Fitness-to-Drive scale, based on Neukum & Krüger (2003).

5.1.5 Definition of success and stop criterion, including the development of research questions for a follow-up project

The treatment can be cancelled anytime by request of the patient. The planned project will be a pilot study with a small sample. Because of this, the decision to continue the project shall not be based on a confirmatory proven difference between treatment and waiting group with regard to the number of responders and non-responders. Descriptively, however, the percentage of responders (patients who complete the driving test with driving instructor entirely and with adequate driving behaviour after one week with vs. without treatment, see chapter 5.1.4) should be distinctly higher for the treatment group than for the waiting group. Schwarzer & Schumacher (2007) state a maximum deviation of 20% for equivalent responder rates in clinical therapy studies. Based on this, the effectiveness of the presented therapy will be seen as validated and the application for a follow-up project will be justified if the percentage of responders

- in the waiting group on day 8 will be 30% ($n \leq 3$) at the max *and*
- in the treatment group on day 5 will be 60% ($n \geq 6$) at the least and thus twice as high minimum

A percentage of at least 60% of responders in the waiting group on day 12 (i.e. after the belated treatment) will be pursued, but will not be postulated.

The secondary criteria of effectiveness are supposed to be decreasing SUD-ratings, decreasing heart rate within and between the sessions and a reduced startle response during the reading exposure. The avoidance behaviour during the BAT as recorded by the psychotherapist, changes in concentration performance during the d2, data regarding driving behaviour at the beginning of the treatment and at the follow-up, accident fear and symptoms regarding depression, PTSD and anxiety disorders in general (Beck Inventories, PSS-SR, AFQ) will not be seen as criteria for treatment success, but will be evaluated exploratory.

The research questions for the planned follow-up project can only be determined after having completed the pilot study. Possible questions might be for example:

- What kind of characteristics of the patient or the preceding accident predict a high success of therapy?
- How should a therapy including the module “Virtual Exposure in the driving simulation” be designed ideally?

- How effective is such a therapy as compared to the classic in-vivo therapy?
- What minimum configuration of the driving simulator will be necessary for therapeutic exposure?
- To what extent will the driving scenarios of the exposure have to be individualised? To what extent can the scenarios that have been developed in the scope of the proof of concept be used in the sense of a catalogue?

5.2 Allocation of responsibilities within the research cooperation

The exact allocation of tasks can be learned from chapters 5.1 and 5.5. In principle, all psychotherapeutic tasks will be taken care of by the psychotherapeutic outpatient clinic. The WIVW will be responsible for project coordination, programming and operation of the driving simulation as well as for rating the patients' driving behaviour.

5.3 Actions of quality assurance

All of the studies at the WIVW are conducted within the framework of a SOP-system, which has been established in 2008, in order to ensure quality. Particularly, SOPs specific for clinical studies are contained which have been developed on the basis of ICH/GCP and thus guarantee compliance with them. The declaration of Helsinki of the World Medical Association in its recent version (Fortaleza, 2013) is considered, too.

5.4 Involvement of target groups and a research advisory committee

The project is supposed to be conducted and evaluated with a subgroup of the later target group: professional motorists with strong driving fear resulting from a work accident (see chapter 5.1.1). For the moment, a research advisory committee is not planned. However, employees of the IAG are supposed to be involved, as they have had promising experience with the initially reported probatory exposures in their driving simulator and have initiated the project. This experience has already been very helpful for planning the project (regarding the definition of inclusion and exclusion criteria among others) and will surely be helpful for conducting the study.

5.5 Detailed working plan and time schedule with allocation of the partners

The research project consists of four working packages (WP). A duration of 18 months and a volume of work of 15.71 man-months (8.80 for the WIVW, 6.91 for the psychotherapeutic outpatient clinic[4], HA) are assessed. The driving school Kwiotek in Würzburg will be subcontracted for the organisation and conduction of the driving test under real driving conditions. The medical study centre Würzburg headed by the neurological specialists Dr. med. Klein and Dr. med. Oehler will be subcontracted for the medical examination. Both institutions have been chosen because of successful cooperation with the WIVW in past studies.

The different WPs with the related work steps are as follows:

WP1: Preparation of the study (man-months: 1 WIVW, 2 HA)

Different preparatory works serve as a bias for WP2:

[4] Costs for travelling and accommodation as well as proportionally for therapy (10 sessions per patient) will be absorbed by BG Verkehr. For time management it has to be taken into account that the writing of the final report is not eligible for funding. It will take additional 1.5 man-months which are not included in the calculated duration of 18 months and thus will prolong it correlatively.

- Preparation of a therapy manual by HA, review by WIVW
- Preparation of an ethics amendment by WIVW, review by HA
- Preparation and compilation of materials (questionnaires/observation sheets related to driving performance) by WIVW
- Preparation and compilation of materials (questionnaires/ observation sheets related to therapy / symptoms) by HA
- Design and testing of the driving test under real driving conditions with the application S.A.F.E. which will serve a standardized driving behaviour observation by WIVW

WP2: Study conduction

WP2.1: Conduction of the block therapy (man-months: 2.46 WIVW, 3.66 HA[5])

Conduction of the block therapy with 2 x 10 patients, as described in chapter 5.1.2:

- Screening call / recruitment, anamnesis session with the offer of/supervision in a driving test with driving instructor (pre-measurement), seven psychotherapeutic sessions (5 of them with exposure in the driving simulation), one psychotherapeutic closing session with debriefing and offer of / supervision in a driving test with driving instructor (post-measurement), booster session by phone, follow-up interview by HA
- Traffic psychological supervision in order to assess driving performance during the driving test under real driving conditions and during the five exposure sessions in the driving simulation (including application S.A.F.E., operation of the driving simulation) by WIVW

WP2.2: Implementation of the developed hierarchy of driving scenarios into the driving simulation (man-months: 1.5 WIVW)

The programming of the courses with the SILAB software can only begin after the anamnesis session and will be realised parallel to the first psychotherapeutic sessions. The possibility for further adaptations / additions which will only appear during the course of treatment will be given.

WP3: Analysis, evaluation, and dissemination of the results (man-months: 2.79 WIVW, 1 HA)

Subsequent to the block therapy the collected parameters will be analysed statistically in order to evaluate the effectiveness of the therapy. The decision to apply for a follow-up project will be based on these results. An early transfer of the project results is to be achieved by publications in journals and presentations at conventions (DGPPN, DGVP/DGVM, Fit to Drive). This results in the following work steps:

- Preparation of the data from the startle response so that it can be analysed statistically by the HA
- Data entry and synchronisation of the different data sources (driving simulation / S.A.F.E., startle response / varioport, heart rate, questionnaires and observation sheets) as well as development of a data entry mask by WIVW
- Statistical analysis by WIVW
- Report writing by WIVW

[5] As already described the costs of the psychotherapeutic outpatient clinic for conducting the therapy will proportionally be absorbed by BG Verkehr. These are not included in the 3.5 man-months.

- Report reviewing by HA
- Decision on follow-up project and development of *traffic psychological* adaptions, questions, and design by WIVW
- Decision on follow-up project and development of *psychotherapeutic* adaptions, questions, and design by HA
- Presentation of the results at *traffic psychological* congresses by WIVW
- Presentation of the results at *psychotherapeutic* congresses by HA

WP4 Project management and project meetings (man-months: 1.02 WIVW, 0.25 HA)

As the pilot study is a joint project with subcontractors, there will continuously be different tasks regarding project coordination, which will be performed by WIVW. Furthermore, there will be at least five project meetings for communicating the project and its preceding (once prior to, twice during and once after data collection, once after statistical analysis).

Table 2: Man-months (rounded) per project month for each work package. Man-months of the HA are green, man-months of the WIVW are blue).

month / WP	1	2	3	4	5	6	7	8	9	10	11	12	13	14	15	16	17	18	Σ
1	0.5	0.5																	1.00
	1	1																	2.00
2.1			0.21	0.21	0.21	0.21	0.21	0.21	0.21	0.21	0.21	0.21	0.21	0.21					2.46
			0.31	31	31	31	31	31	31	31	31	31	31	0.31					3.66
2.2			0.13	0.13	0.13	0.13	0.13	0.13	0.13	0.13	0.13	0.13	0.13	0.13					1.50
3								0.5			0.5				0.5	0.5	0.5	0.29	2.79
								0.13			0.13				0.25	0.25	0.14	0.1	1.00
4	0.06	0.06	0.06	0.06	0.06	0.06	0.06	0.06	0.06	0.06	0.06	0.06	0.06	0.06	0.06	0.06	0.06	0.06	1.02
		0.05				0.05				0.05				0.05			0.05		0.25

6 EXPECTED RESULTS

It is expected that at least 2/3 of the patients with driving fear due to a traffic accident will be treated successfully by the described therapy method (see chapter 5.1.5). If this expectation is confirmed, a larger evaluation study is to be applied for subsequently in order to identify predictive characteristics of patients and accidents for this kind of therapy and to develop an ideal therapy design. Thus, a comparison with a mere in-vivo therapy will be possible. It is the long-term objective to establish the developed therapy into therapeutic praxis (see next chapter).

7 PRACTICAL IMPLEMENTATION OF THE RESULTS

As the reported project is a pilot study no practical implication of the results can be expected immediately afterwards. In the case of validated effectiveness it should rather be applied for a follow-up project in order to form a basis for implementing the therapy form into therapeutic praxis (also see chapter 5.1.5). It is the long-term goal to offer the program that has been developed in this project to various workers' compensation boards, but also to different psychotherapeutic clinics. As described in chapter 4, the treatment would be possible at the driving simulation of the IAG, as it is operated by the SILAB software as well. By building up driving simulators at further workers' compensation boards and clinics, a nation-wide treatment is supposed to be initiated within 5 years.

With regard to questions such as fitness to drive among elderly drivers, under the influence of drugs and with diseases (as dementia, Parkinson's disease, stroke) the driving simulation is already becoming established as a promising tool for diagnosis as well as for maintaining and rehabilitating fitness to drive. Simulation is seen as superior to traditional methods of driver fitness determination (see Hartje, 2004, or Kaussner, 2007), because scenarios can be designed that are tailored to the particular symptoms. The scenarios can be standardized, are repeatable, and can be offered without any danger. Moreover, patients can compensate for performance impairments just like under real traffic conditions, thus experiencing a high face validity. Several clinics are already interested in establishing driving simulators.

Multiple clinics showed great interest towards WIVW in the assembly of a driving simulator. Including clinics whose employees are affiliated to the research group fitness to drive of the Gesellschaft für Neuropsychologie (German society for neuropsychology) and for this reason have been in contact with the WIVW for many years (Dr. Becker Klinikgesellschaft mbH & Co. KG, Kiliani-Klinik Bad Windsheim, Klinikum Karlsbad-Langensteinbach, Sachsenklinik, Evangelisches Geriatriezentrum Berlin GmbH/ Medizinische Fakultät der Humboldt-Universität zu Berlin (Charité), Campus Virchow-Klinikum, Bezirksklinikum Maikofen Deggendorf, Kliniken Schmieder). The list of clinics with interest in driving simulation was complemented by 50 additional clinics when the WIVW exhibited at the conference of the German society for neuropsychology in 2013. The Inn-Salzach-Klinikum in Wasserburg am Inn already has a driving simulator with the simulation software SILAB, which has been developed by the applicant, a scenario packet "driver fitness and ability" and the application S.A.F.E. The psychiatric and neurological department would surely be interested in extending the possible application of the driving simulation regarding therapeutic measures.

8 REFERENCES

Beck, J. G., Palyo, S. A., Winer, E. H., Schwagler, B. E., & Ang, E. J. (2007). Virtual reality exposure therapy for PTSD symptoms after a road accident: An uncontrolled case series. Behavior Therapy, 38, 39-48.

Beck, A. T., & Steer, R. A. (1993). Manual for the Beck Depression Inventory. San Antonio, TX: Psychological Corporation.

Beck, A. T., Steer, R. A., & Brown, G. K. (1996). Manual for the Beck Depression Inventory - II. San Antonio, TX: Psychological Corporation.

Blanchard, E. B., & Hickling, H. J. (2004). What are the psychosocial effects of MVAs on survivors? In E. B. Blanchard, E. J. Hickling (Eds.), After the crash: Psychological assessment and treatment of survivors of motor vehicle accidents (2ª ed., pp. 57-97).Washington, DC: American Psychological Association.

Brickenkamp, R., Schmidt-Atzert, L. & Liepmann, D. (2010). d2-R: Test d2 - Revision. Hogrefe, Göttingen.

Brenner-Hartmann, J. (2002). Durchführung standardisierter Fahrverhaltensbeobachtungen im Rahmen der medizinisch-psychologischen Untersuchung (MPU). Vortrag beim 38. BDP-Kongress für Verkehrspsychologie in Regensburg, 2002. http://www.bdp-verkehr.de/backstage2/ver/documentpool/kongress/brenner_01.pdf.

Emmelkamp, P.M., Krijn, M., Hulsbosch, A.M., deVries, S., Schuemie, M.J. & van der Mast, C.A. (2002). Virtual reality treatment versus exposure in vivo: a comparative evaluation in acrophobia. Behavior Research and Therapy. 40(5), 509-16.

Foa, E. B., & Kozak, M. J. (1986). Emotional processing of fear: Exposure to corrective information. Psychological Bulletin, 99, 20-35.

Foa, E.B., Cashman, L., Jaycox, L. & Perry, K. (1997). The validation of a self-report measure of posttraumatic stress disorder : the Posttraumatic Diagnostic Scale. In: Psychological Assessment, 9, 445–451.

Foa, E. B., & Meadows, E. A. (1997). Psychosocial treatments for posttraumatic stress disorder: A critical review. Annual Review of Psychology, 48, 449-480.

Folstein, J. F., Folstein, S. E., & McHugh, P. R. (1975). “Mini-mental state”: A practical method for grading cognitive state of patients for the clinician. Journal of Psychiatric Research, 43, 882-885.

Garcia-Palacios, A., Botella, C., Hoffman, H. & Fabregat, S. (2007). Comparing Acceptance and Refusal Rates of Virtual Reality Exposure vs. In Vivo Exposure by Patients with Specific Phobias. CyberPsychology & Behavior, 10 (5), 722-724.

Garcia-Palacios, A., Hoffman, H., Carlin, A., Furness, T. A. III, & Botella, C. (2002). Virtual reality in the treatment of spider phobia: A controlled study. Behaviour Research and Therapy, 40, 983-993.

Hamm, A. (2006). Spezifische Phobien. Göttingen: Hogrefe.

Hartje, W. (2004). Die neuropsychologische Testdiagnostik hat nur begrenzte Aussagekraft für die Fahreignung. In: C. Dettmers & C. Weiller (Hrsg.), Fahreignung bei neurologischen Erkrankungen (S. 19-22). Bad Honnef: Hippocampus Verlag.

Hoffmann, S. & Buld, S. (2006). Darstellung und Evaluation eines Trainings zum Fahren in der Fahrsimulation. In VDI-Gesellschaft Fahrzeug- und Verkehrstechnik (Hrsg.), Integrierte Sicherheit und Fahrerassistenzsysteme (VDI-Berichte, Nr. 1960, S. 113-132). Düsseldorf: VDI-Verlag.

Holmes, T.H. & Rahe, R.H. (1967). "The Social Readjustment Rating Scale". Journal of Psychosomatic Research 11 (2), 213–218.

Kaussner, Y. (2007). Fahrtauglichkeit bei M. Parkinson. Dissertation. Bayerische Julius-Maximilans-Universität Würzburg. Verfügbar unter http://opus.bibliothek.uni-wuerzburg.de/volltexte/2007/2250/

Kennedy, R.S., Lane, N. E., Berbaum, K.S., & Lilienthal, M.G. (1993). Simulator Sickness Questionnaire: An Enhanced Method for Quantifying Simulator Sickness. The International Journal of Aviation Psychology, 3, 203-220.

König, J. (2012). Posttraumatische Belastungsstörung: Ein Manual zur Cognitive Processing Therapy. Göttingen: Hogrefe.

Kuch, K., Cox, B. J. & Direnfeld, D. M. (1995). A brief self-rating scale for PTSD after road vehicle accident. Journal of Anxiety Disorders, 9, 503-514.

Maercker, A. (2003). Therapie der posttraumatischen Belastungsstörungen (2nd ed.). Berlin, Heidelberg: Springer-Verlag.

McNally, R. J. (2007). Mechanisms of exposure therapy: How neuroscience can improve psychological treatments for anxiety disorders. Clinical psychology review, 27, 750-9.

McLay, R. N., McBrien, C., Wiederhold, M. D., & Wiederhold, B. K. (2010). Exposure therapy with and without virtual reality to treat PTSD while in the combat theater: A parallel case series. Cyberpsychology, Behavior, and Social Networking, 13, 37-42.

Michaliszyn, D., Marchand, A., Bouchard, S., Martel, M.-O., & Poirier-Bisson, J. (2010). A randomized, controlled clinical trial of in virtuo and in vivo exposure for spider phobia. Cyberpsychology, Behavior, and Social Networking. 13, 689-695.

Mühlberger, A., Herrmann, M. J., Wiedemann, G., Ellgring, H. & Pauli, P. (2001). Repeated exposure of flight phobics to flights in virtual reality. Behaviour Research and Therapy, 39, 1033-1050.

Mühlberger, A., & Pauli, P. (2011). Virtuelle Realität in der Psychotherapie. PiD - Psychotherapie im Dialog, 2, 143-147.

Mühlberger, A., Wiedemann, G. & Pauli, P. (2003). Efficacy of a one-session virtual reality exposure treatment for fear of flying. Psychotherapy Research, 13, 323-336.

Neukum, A. & Krüger, H. P. (2003). Fahrerreaktionen bei Lenksystemstörungen - Untersuchungsmethodik und Bewertungskriterien. In: VDI-Gesellschaft Fahrzeug- und Verkehrstechnik (ed.). Reifen-Fahrwerk-Fahrbahn. VDI-Berichte, Nr. 1791. VDI-Verlag, Düsseldorf.

Nübling, R., Schmidt, J. & Schulz, H. (2002). FPTM Fragebogen zur Psychotherapiemotivation. In E. Brähler, J. Schuhmacher & B. Strauß (Hrsg.), Diagnostische Verfahren in der Psychotherapie. Göttingen: Hogrefe.

Nyberg, E., Stieglitz, R. D., Frommberger, U., & Berger, M. (2003). Psychological disorders after severe occupational accidents. Versicherungsmedizin, 55, 76-81.

Powers, M. B., & Emmelkamp, P. M. G. (2008). Virtual reality exposure therapy for anxiety disorders: A meta-analysis. Journal of Anxiety Disorders, 22(3), 561-569.

Rothbaum, B. O., Meadows, E. A., Resick, P., & Foy, D. W. (2000). Cognitive-behavioral therapy. In Foa, E. B., Keane, T. M., Friedman, M. J. (Eds.), Effective treatments for PTSD: Practice guidelines from the International Society for Traumatic Stress Studies (pp. 320-325). New York, NY: Guilford Press.

Rothbaum, B. O. (2009). Using virtual reality to help our patients in the real world.Depression and Anxiety, 26(3), 209-211.

Schaarschmidt, U. & Fischer, A. (2003). AVEM - Arbeitsbezogenes Verhaltens- und Erlebensmuster. Handanweisung. Zweite überarbeitete und erweiterte Auflage. Frankfurt: Swets & Zeitlinger.

Schwarzer, G., & Schumacher, M. (2007). Die Beurteilung der Gleichwertigkeit von Behandlungen. In M. Schumacher & G. Schulgen (Eds.), Methodik klinischer Studien. Methodische Grundlagen der Planung, Durchführung und Auswertung (2. Aufl. ed., pp. 113-127). Berlin: Springer.

Statistisches Bundesamt (2014). Verkehrsunfälle –Fachserie 8 Reihe 7 – Oktober 2013. Wiesbaden. Verfügbar unter: https://www.destatis.de/DE/Publikationen/Thematisch/TransportVerkehr/Verkehrsunfaelle/VerkehrsunfaelleMonat/VerkehrsunfaelleM2080700131104.pdf?__blob=publicationFile

Traue, H.C., Hrabal, V. & Kosarz, P. (2000). AlltagsBelastungsFragebogen (ABF): Zur inneren Konsistenz, Validierung und Stressdiagnostik mit dem deutschsprachigen Daily Stress Inventory. Verhaltenstherapie und Verhaltensmedizin, 21, 15-38.

Wald, J. (2004). Efficacy of virtual reality exposure therapy for driving phobia: A multiple baseline across-subjects design. Behavior Therapy, 35, 621-635.

Wald, J., & Taylor, S. (2000). Efficacy of virtual reality exposure therapy to treat driving phobia: A case report. Journal of Behavior Therapy and Experimental Psychiatry, 31, 249-257.

Wald, J., & Taylor, S. (2003). Preliminary research on the efficacy of virtual reality exposure therapy to treat driving phobia. CyberPsychology & Behavior, 6, 459-465.

Walshe, D., Lewis, E., O'Sullivan, K., & Kim, S. I. (2005). Virtually Driving: Are the driving environments "real enough" for exposure therapy with accident victims? An explorative study. CyberPsychology & Behavior, 8, 532-537.

Zöllner, T., Karl, A., Märcker, A., Hickling, E. J. & Blanchard, B. (2005) Manual zur Kognitiven Verhaltenstherapie von Posttraumatischen Belastungsstörungen bei Verkehrsunfallopfern.
