# Supplementary material for: Treating patients with driving phobia by virtual reality exposure therapy – a pilot study
Source: PLoS One. 2020 Jan 7;15(1):e0226937. doi: 10.1371/journal.pone.0226937 (PMC6946146; doi:10.1371/journal.pone.0226937)
Supplement: S2 Protocol — (PDF) [file pone.0226937.s005.pdf]

Prüfplan

Entwicklung und Evaluation einer Expositionstherapie in der Fahrsimulation zur Behandlung von Fahrangst nach Verkehrsunfällen

- Eine Pilotstudie

Dr. Yvonne Kaussner[1]
Dr. Ramona Kenntner-Mabiala[1]
Dr. Nora Walz[2]
Dr. Petra Markel[2]
Prof. Paul Pauli[2]

[1]**WIVW GmbH**
Würzburger Institut für Verkehrswissenschaften
Robert-Bosch-Straße 4
97209 Veitshöchheim

[2]**Hochschulambulanz für Psychotherapie**
Institut für Psychologie
Universität Würzburg
Marcusstraße 9-11
97070 Würzburg

Amended Version 24.09.2015

1 PROBLEMSTELLUNG

Jährlich ereignen sich in Deutschland etwa 2,5 Millionen Verkehrsunfälle, etwa 300.000 davon mit Personenschaden, wobei knapp 400.000 Menschen verunglücken (d.h. leicht verletzt, schwer verletzt oder getötet werden; s. Statistisches Bundesamt, 2014).

Nach einem Unfall leiden in den ersten Wochen bis zu 50% der Betroffenen an Belastungssymptomen, ca. 8% entwickeln eine Posttraumatische Belastungsstörung (PTBS). Das bedeutet, dass ungefähr jeder 12. Betroffene eines mittelschweren bis schweren Verkehrsunfalls eine behandlungsbedürftige Posttraumatische Belastungsstörung entwickelt (Maercker, 2003). Allerdings können auch weitere psychische Störungen, wie akute Belastungsreaktionen, Anpassungsstörungen oder spezifische (isolierte) Phobien auftreten, die zu einer Fahrangst nach Verkehrsunfällen führen können, so dass insgesamt mit einer höheren psychischen Morbidität von bis zu 30% zu rechnen ist (Nyberg et al., 2003). Die daraus resultierende „Fahrangst" kann sich auf spezielle Situationen, wie bspw. Fahren auf der Autobahn oder Fahren unbekannter Strecken beziehen oder auf das Fahren im Straßenverkehr ganz allgemein. Dabei kreisen die Gedanken um Katastrophen im Straßenverkehr oder um Befürchtungen, einen Unfall zu verursachen und andere zu verletzen. Beim Versuch, trotz der Angst zu fahren, reagieren die Betroffenen mit starken körperlichen Symptomen, was dazu führt, dass solche Situationen und das Autofahren vermieden werden.

Bei Berufskraftfahrern kann diese Fahrangst zu einer Minderung der Erwerbsfähigkeit oder sogar zur Berufsunfähigkeit führen. Aber auch für Personen anderer Berufe kann die Fahrangst den beruflichen Wiedereinstieg nach einem Verkehrsunfall behindern, wenn es nicht mehr möglich ist, den Weg zur Arbeitsstätte mit dem Auto zurückzulegen.

Die kognitive Verhaltenstherapie und hier insbesondere die Expositionstherapie gilt als Methode der Wahl zur Behandlung von Angststörungen, wobei zunehmend auch virtuelle Verfahren eingesetzt werden (Mühlberger & Pauli, 2011). Nur vereinzelt gibt es bislang Erfahrungen und Studien zur Wirksamkeit von expositionstherapeutischen Maßnahmen in der Fahrsimulation, wobei diese als höchst vielversprechend anzusehen sind (z.B. Beck et al., 2007). Insbesondere zeigte sich dies in Einzelfall-Untersuchungen, die im Fahrsimulator des Instituts für Arbeit und Gesundheit (IAG) der DGUV mit verunglückten Berufskraftfahrern durchgeführt wurden und auch aktuell werden. Auffällig ist allerdings, dass sich die Intervention bei manchen Personen als enorm effizient erweist, während andere in der Simulation keinerlei Angst verspüren und dadurch auch keinen therapeutischen Erfolg erfahren. Da es nach Einschätzung des IAG in der alltäglichen Zusammenarbeit mit Berufsgenossenschaftskliniken nicht möglich ist, eine systematische Untersuchung darüber aufzustellen, welche Faktoren dazu beitragen, dass eine Exposition in der Fahrsimulation erfolgreich ist, trat das IAG mit dem ausdrücklichem Wunsch nach einer systematischen Untersuchung an das Würzburger Institut für Verkehrswissenschaften (WIVW) als forschungsbetreibenden Zulieferer des IAG-Fahrsimulators heran. Die Kooperation mit der Hochschulambulanz für Psychotherapie der Universität Würzburg bietet sich aufgrund deren Expertise in der Erforschung und Behandlung von Angststörungen sowie in der virtuellen Expositionstherapie an (siehe Focus Ärzteliste 2013).

Viele Patienten verweigern aufgrund starker Ängste eine klassische Therapie mit einer Exposition in vivo oder brechen diese vorzeitig ab. Eine vorgeschaltete virtuelle Exposition könnte hier Abhilfe schaffen, da den Patienten zumindest kognitiv bewusst ist, dass keine reale Ge-

fahr besteht. So berichten z.B. Garcia-Palacios et al. (2007), dass 27% der untersuchten Patienten mit einer spezifischen Phobie eine in vivo Exposition verweigern, während nur 3% eine virtuelle Exposition ablehnen. Vor die direkte Wahl gestellt, entscheiden sich 76% der Patienten für eine virtuelle Exposition und somit gegen eine Exposition in vivo. Der Vorteil einer (vorbereitenden) Exposition in der Fahrsimulation im Vergleich zu einer reinen In-Vivo-Exposition liegt somit zunächst in einem niederschwelligeren Zugang zur Exposition, die einen entscheidenden Therapiebaustein in der Angstbehandlung darstellt. Darüber hinaus bieten sich aber auch weitere Vorteile, wie

- Therapeutische Kontrolle: Die Exposition kann stets und unmittelbar abgebrochen werden
- Situative Kontrolle: Die relevanten Situationen können gezielt hergestellt und hierarchisch dargeboten werden; man muss sie nicht im realen Verkehr aktiv aufsuchen oder abwarten, bis sie zufällig eintreten (z.B. Überholmanöver eines entgegenkommenden Fahrzeugs, Passieren eines Unfalls, Einsatz-/Rettungsfahrzeuge, Regen). Darüber hinaus treten in der virtuellen Exposition keine unerwarteten, nicht gewollten Situationen auf, was bei einer in vivo Exposition nicht kontrolliert werden kann.
- Auslösung von Erinnerungen an Umstände/Trigger im Verlauf der Therapie, die zuvor nicht bewusst waren und in der Simulation umgesetzt werden können
- Keine tatsächliche Gefährdung (wobei dies nicht nur sicherheitsrelevant, sondern für den Patienten auch von hoher kognitiver Bedeutung ist).

In der vorliegenden Vorhabensbeschreibung soll daher eine Pilotstudie vorgestellt werden, in deren Rahmen eine Expositionstherapie in der Fahrsimulation entwickelt und anhand einer kleinen, sorgfältig ausgewählten Patientenstichprobe evaluiert werden soll. Positive Ergebnisse vorausgesetzt, sollen in einer anschließend zu beantragenden größeren Evaluationsstudie prädiktive Erfolgskriterien für eine solche Therapie identifiziert und eine optimale Gestaltung derselben herausgearbeitet werden. Langfristig wird angestrebt, die entwickelte virtuelle Exposition in die therapeutische Praxis einzuführen.

2 STAND DER ERKENNTNISSE

2.1 Nachweis einer systematisch durchgeführten Literaturrecherche, Beschreibung der Suchstrategie

Für einen umfassenden Überblick zum aktuellen Stand der Forschung zur Therapie von Fahrangst, wurde eine systematische Literaturrecherche durchgeführt. Hierzu wurde das Datenbank-Infosystem (DBIS) der Universitätsbibliothek Würzburg mit Zugang zu 72 Datenbanken herangezogen. Es wurde auf die Datenbanken PsycINFO, PSYNDEX und PsychTHERAPY aus dem Fachbereich „Psychologie“ zurückgegriffen. Als Suchkriterien wurden folgende Stichwörter in allen Kombinationen verwendet:

- Virtual reality
- Exposure therapy
- Efficacy virtual exposure therapy
- Anxiety disorders
- PTSD (posttraumatic stress disorder)
- Driving phobia
- Fear of driving
- Road accident

- Motor vehicle / traffic accident.

Eingeschlossen wurden systematische Reviews, Metaanalysen, Einzelfallstudien, quasi- und randomisierte experimentelle Studien.

2.2 Zusammenfassung relevanter Forschungsarbeiten (eigene und externe)

Die Virtuelle Realität (VR) wird zunehmend in der Psychotherapie eingesetzt. Besondere Bedeutung hat die Expositionstherapie in der virtuellen Realität (VRET) in der Behandlung von Angststörungen, bei denen die Expositionstherapie breite Evidenz besitzt (Powers & Emmelkamp, 2008). VRET funktioniert genauso wie Exposition in vivo oder in sensu, mit dem Unterschied, dass der angstauslösende Reiz oder die angstauslösende Situation von einem Computer simuliert werden (Rothbaum, 2009). Von Patienten wird VRET gegenüber einer Exposition in vivo klar bevorzugt (Garcia-Palacios et al., 2007). Der offenkundigste Vorteil von VRET gegenüber der klassischen Exposition in vivo ist demnach der niederschwelligere Zugang, geringere Abbruchquoten und eine höhere Therapiebereitschaft der Patienten. Einen weiteren Vorteil bietet sie dann, wenn Stimuli oder Situationen in vivo nicht leicht herstellbar oder auffindbar sind, aber gut kontrolliert werden müssen. Außerdem können auch Situationen hergestellt werden, in denen eine bestimmte psychische oder somatische Symptomatik ein Sicherheitsrisiko darstellt. Dies trifft zum Beispiel auf das Autofahren zu.

Besonders spezifische Phobien und die PTBS werden bisher in VR behandelt. Dies hat damit zu tun, dass bei diesen Störungen der angstauslösende Reiz oder die angstauslösende Situation oft besonders klar umrissen und deshalb gut simulierbar sind (z.B. eine Spinne oder ein Kriegsschauplatz). Gute Evidenz für die Wirksamkeit von VRET liegt in eigenen Forschungsarbeiten des Antragstellers (Lehrstuhl für Psychologie I der Universität Würzburg) zum Beispiel für die Behandlung von Flugphobie vor. Die vom Antragsteller durchgeführten Studien weisen nach, dass sowohl mehrere Flüge in VR (Mühlberger et al., 2001) als auch ein einzelner VR-Flug (Mühlberger et al., 2003) Flugangst signifikant reduzieren. Auch bei Spinnenangst ist die Wirksamkeit einer Behandlung mit Hilfe der VRET gut belegt (Garcia-Palacios et al., 2002). Studien, in denen VRET mit einer Exposition in vivo verglichen wurde, weisen für beide Therapieformen eine ähnliche Wirksamkeit nach (z.B. Emmelkamp et al., 2002; Rothbaum et al., 2000; McLay et al., 2010; Michaliszyn et al., 2010).

Verkehrsunfallopfer, die eine Fahrangst ausbilden, zeigen ein deutlich verändertes Fahrverhalten. Sie fahren entweder sehr übervorsichtig, was mit einer Behinderung anderer Verkehrsteilnehmer einhergehen kann, oder trauen sich überhaupt nicht mehr zu fahren (Maercker, 2003). Gleichzeitig werden Stimuli vermieden, die an den Unfall erinnern, da sie eine starke körperliche Angstreaktion auslösen. Eine starke Erregung ist aber notwendig, um eine Korrektur der Furchtstruktur anzuregen (Foa & Kozak, 1986; McNally, 2007). Dies greift die Expositionstherapie auf, deren Wirkung bei der Behandlung der PTBS und Phobien klar belegt ist (Rothbaum et al., 2000; Foa & Meadows, 1997). Der Patient wird nach einer ausführlichen Vorbereitung (Krankheitsanamnese, Störungsmodell, Angsthierarchie, Veränderungsrational) wiederholt mit den angstauslösenden Stimuli konfrontiert, ohne diese vermeiden zu können. Dabei muss besonders auf subtil eingesetztes Vermeidungsverhalten geachtet werden (z.B. kognitive Ablenkung), denn nicht immer wird die Situation komplett vermieden. Eine vollkommene Aussetzung gegenüber der Angst und den angstauslösenden Stimuli ist aber wichtig, um eine kognitive Umstrukturierung zu erreichen. Durch wiederholte und ausreichend lange Exposition soll der Patient auch an die Angstsymptome habituieren.

Beck et al. (2007) konnten an sechs Patienten, die nach einem Verkehrsunfall eine PTBS ausgebildet hatten, zeigen, dass die Behandlung mit VRET, in der die Betroffenen in einem bewegten Fahrsimulator behandelt wurden, eine Reduktion einer PTBS-Symptomatik bewirkt. Dabei fanden insgesamt zehn Therapiesitzungen statt, von denen die ersten beiden der Vorbe-

reitung auf die Exposition dienten. Elemente waren die Aufklärung über die PTBS, Erlernen eines Entspannungsverfahrens und Erstellen einer Angsthierarchie. Daran schlossen sich acht Expositionssitzungen an. Die Studie konnte insgesamt belegen, dass sich Symptome wie Wiedererleben, Vermeidungsverhalten und emotionales Abgestumpftsein von Prä- zu Posttreatment deutlich reduzierten (ES: d = .79 – d = 1.49). Gleichzeitig konnten die Autoren auch zeigen, dass diese Verbesserung wahrscheinlich nicht auf einen alleinigen Rückgang der Depression oder anderer Angstsymptome zurückgeführt werden konnte. Die Zufriedenheit der Patienten mit diesem Vorgehen war sehr hoch.

Die Wirksamkeit der VRET konnte auch für Patienten mit Fahrphobie nachgewiesen werden. In einer Einzelfallstudie von Wald und Taylor (2000) wurde eine Person mit Fahrangst in einem Zeitraum von 10 Tagen im Rahmen von drei Therapiesitzungen mit VRET in einem statischen Fahrsimulator behandelt, wobei sie vier unterschiedliche Szenarien mit ansteigender Schwierigkeit durchfahren musste. Der Fahrsimulator bestand aus einem Computerdisplay mit Lenkrad und Gaspedal. Die Fahrumgebung wurde über ein Head-mounted Display dargeboten. Es wurde nur dann zum nächst schwierigeren Szenario übergegangen, wenn die Patientin auf einer Angstskala von 0-100 nur noch einen Wert von 10 oder weniger angab. Die Patientin zeigte nach drei Behandlungen eine Reduktion ihrer Angstsymptome und ihres Vermeidungsverhaltens. Der Therapieerfolg war bis zur Follow-up-Untersuchung nach sieben Monaten stabil.

Wald (2004) untersuchte die Wirksamkeit der VRET an fünf Patienten mit Fahrphobie. Drei von fünf Patienten zeigten zwischen der Vor- und Nach-Behandlung eine deutliche Besserung ihrer Fahrangst und wiesen weniger Vermeidungsverhalten auf. Bei einem Patienten führte die VRET nur marginal zu einer Verbesserung der Symptomatik. Ein weiterer Patient profitierte nicht von der Behandlung. Bei allen fünf Patienten konnte die Fahrhäufigkeit durch den Einsatz von VRET nicht gesteigert werden. Aufgrund der vorliegenden Evidenz zur Wirksamkeit der VRET bei Fahrangst (Wald & Taylor, 2000; Wald, 2004) ist diese als zusätzliche Behandlung beziehungsweise als vorbereitende Behandlung für eine in vivo Exposition der Fahr-Phobie zu empfehlen, nicht aber als alleinige Therapieform (Wald & Taylor, 2003).

2.3 Defizitanalyse

Die dargestellten Studien liefern erste Hinweise darauf, dass eine Fahrangst nach einem Autounfall mit Hilfe der VRET behandelbar ist. Jedoch handelt es sich dabei um Einzelfallstudien oder um nicht kontrollierte Fallstudien (keine Vergleichsgruppe) mit kleinen Stichproben, so dass keine endgültigen Aussagen über den spezifischen Effekt der VRET getroffen werden können. Darüber hinaus wurde in der Studie von Beck et al. (2007) der Einfluss der VRET-Behandlung auf spezifische Symptome der PTBS, jedoch nicht auf tatsächliches Fahrverhalten untersucht. Zudem zeigen sowohl die wenigen publizierten Studien als auch die Erfahrungen am IAG, dass zwar manche Personen sehr stark, andere aber wiederum gar nicht von der VRET profitieren, wobei bislang keine differenzierenden oder prädiktiven Merkmale bekannt sind. Weitere Studien mit einer höheren Anzahl an Patienten, mit einem kontrollierten randomisierten Design sowie verschiedenen abhängigen Variablen auf behavioraler, subjektiver und physiologischer Ebene sind notwendig, um eine Vielzahl von Forschungs- und Anwendungsfragen zu beantworten:

- Welche Merkmale von Patienten sind gute Prädiktoren für einen Therapieerfolg (z.B. bisherige Therapieerfahrung, Unfallcharakteristiken)?
- Wie wirksam ist eine (vorbereitende) VRET im Fahrsimulator im Vergleich zu einer reinen In-Vivo-Exposition?

- Welche Gestaltungsmerkmale der Fahrsimulatorsitzungen erhöhen den Therapiererfolg (z.B. Grad der nötigen Individualisierung der verwendeten Szenarien oder Ausbaustufe der Fahrsimulation)?
- Wie verändert sich durch VRET das Fahrverhalten?
- Inwiefern können durch eine VRET in der Fahrsimulation Symptome wie Depression, Angst und das Erleben von Flashbacks verbessert werden?

3 ZIELSETZUNG, ZIELGRUPPEN

Patienten

In der geplanten Pilotstudie soll gezeigt werden, dass phobisches Vermeidungsverhalten im Straßenverkehr nach einem Verkehrsunfall durch Konfrontations- bzw. Übungssitzungen in einer virtuellen Realität im Fahrsimulator verringert werden kann. Gelingt es, die Wirksamkeit dieses neuen Therapieelements nachzuweisen, können im Rahmen eines Anschlussprojektes die therapeutische Ausgestaltung optimiert und weitere Forschungsfragen beantwortet werden (s. Abschnitt 2.3).Langfristig soll diese Behandlungsmethode in die psychotherapeutische Praxis eingeführt werden.

Zur Zielgruppe gehören damit Personen, die als Fahrer eines motorisierten Fahrzeugs in einen Verkehrsunfall involviert waren und infolge dessen eine Fahrangst mit Fahrvermeidung entwickelt haben. Mögliche klinisch-psychiatrische Diagnosen sind dabei „Anpassungsstörung“, „spezifische (isolierte) Phobie“ oder PTBS. Um zunächst eine möglichst homogene Gruppe zu untersuchen, werden in die Pilotstudie nur *Berufskraftfahrer* aufgenommen, die der BG Verkehr mit der entsprechenden Symptomatik gemeldet werden. Eine detaillierte Auflistung von Ein- und Ausschlusskriterien findet sich in Abschnitt 5.1.1.

Anwender

Neben den Patienten selbst können auch die Berufsgenossenschaften (BGen) und ihre Trauma-Lotsen, die die Umsetzung einer solchen Expositionstherapie veranlassen können, als spätere Zielgruppe angesehen werden. Im Rahmen dieser Studie wird zwar speziell die BG Verkehr einbezogen, die entwickelte Maßnahme soll aber letztendlich für alle Unfallversicherungsträger (UVT) zugänglich sein.

4 RELEVANZ FÜR DIE GESETZLICHE UNFALLVERSICHERUNG

Nach Schätzungen von Trauma-Lotsen der BG Verkehr müssen jährlich etwa 175 Kraftfahrer wegen Fahrangst nach einem Arbeitsunfall ambulant oder sogar stationär therapiert werden (s. dazu auch Abschnitt 5.1.1). Wird die Fahrangst nicht erfolgreich behandelt, kann dies zu einer Minderung der Erwerbsfähigkeit (MDE) oder sogar zur Berufsunfähigkeit führen. Beträgt die MDE mindestens 20% (SGB VII) erhält der Versicherte von der BG eine Rente. Eine effiziente Therapie der Erkrankung dient demnach dazu, die Patienten schnellstmöglich wieder ihrer beruflichen Tätigkeit zuzuführen bzw. ihre volle Erwerbsfähigkeit wieder herzustellen und Folgekosten zu vermeiden. Im Rahmen der im folgenden Abschnitt beschriebenen Studie soll die Wirksamkeit einer Kurzzeit-Psychotherapie der Fahrangst mit einem neuartigen Mo-

dul „Exposition in der Fahrsimulation“ nachgewiesen werden. Sollte dieser Nachweis gelingen, ist davon auszugehen, dass diese als einwöchige Blocktherapie geplante Behandlung sehr zeiteffizient ist und somit im Vergleich zu den bisher angewandten Standardmethoden auch die Behandlungskosten reduziert werden können. Die Exposition soll in einem Fahrsimulator des WIVW mit der Software SILAB stattfinden. Da der Fahrsimulator am IAG der DGUV mit der gleichen Software betrieben wird, wäre das Expositionsmodul auch dort lauffähig, so dass sich die Therapie der Fahrangst auch für den dortigen Simulator als ein neues Anwendungsfeld erschließen würde.

5 METHODIK

Bei dem Vorhaben handelt es sich um eine Pilotstudie (i.S. eines Proof of Concept), die im Rahmen einer Forschungskooperation zwischen dem WIVW und der Hochschulambulanz für Psychotherapie der Universität Würzburg durchgeführt werden soll.

Ziel ist es, die Wirksamkeit einer Exposition in der Fahrsimulation im Rahmen einer einwöchigen Blocktherapie an einer kleinen Stichprobe von 2x10 Patienten mit Fahrangst (einer Behandlungs- und einer Wartegruppe) zu untersuchen. Die Studie dient explizit nicht dazu, eine „neue“ Therapie zu untersuchen, sondern lediglich die Wirkung eines neuen Therapieelements, nämlich der VRET in der Fahrsimulation, zu überprüfen. Die VRET wird dabei nicht isoliert durchgeführt werden, sondern wird psychotherapeutisch vor- und nachbereitet. Konkret soll geprüft werden, ob eine mit starkem Vermeidungsverhalten verbundene Fahrangst bei Patienten mit den Diagnosen „Anpassungsstörung“, „spezifische (isolierte) Phobie“ oder „PTBS“ nach einem Verkehrsunfall vermindert werden kann, so dass die Patienten nach der virtuellen Exposition wieder mit angemessenem Fahrverhalten am Straßenverkehr teilnehmen können. Zusätzlich wird geprüft, ob durch die virtuelle Exposition auch Depressions- oder Angstsymptome sowie potentiell vorliegende Konzentrationsschwierigkeiten verbessert werden können. Dazu werden verschiedene Parameter auf subjektiver, physiologischer und behavioraler Ebene betrachtet. Die entscheidende Zielgröße zur Erfolgsbeurteilung der VRET in der Fahrsimulation wird aber die Fahrleistung in einer Fahrverhaltensprobe im Realverkehr sein, die im Anschluss an die VRET absolviert wird (und zur Kontrolle auch vorher zumindest angeboten wird). Hierfür werden die Urteile eines Fahrlehrers und eines Verkehrspsychologen herangezogen, die gegenüber der Gruppenzugehörigkeit verblindet sind. Die Wartegruppe dient dabei der Kontrolle von Zeiteffekten, von Spontanremissionen sowie eines möglichen therapeutischen Effekts von studienspezifischen Aktivitäten, die unabhängig von der Exposition stattfinden (medizinische Konsiliaruntersuchung, Anamnesegespräch mit Psychotherapeut, Kontakt mit Fahrlehrer/Verkehrspsychologe). Nur durch den Vergleich mit einer Wartegruppe und eine Verblindung gegenüber der Gruppenzugehörigkeit können zudem Verzerrungen bei der Beurteilung des Fahrverhaltens kontrolliert werden (z.B. ein grundsätzlich milderes Urteil bei der zweiten Fahrprobe *nach* der Therapie, was sonst mit einem positiven Therapieeffekt konfundiert werden würde).

Lässt sich so die Wirksamkeit anhand vorher definierter Kriterien belegen, soll im Rahmen eines im Nachgang zu beantragenden Anschlussprojektes eine Evaluation anhand einer größeren, powerkalkulierten Stichprobe durchgeführt und weitere forschungs- und praxisrelevante Fragen beantwortet werden (wie z.B. die Identifikation von prädiktiven Patientenmerkmalen für einen hohen Therapie-Erfolg oder von Mindestanforderungen an die Ausbaustufe der Fahrsimulation). Ziel des Anschlussprojektes wäre damit, die spätere Anwendung in der therapeutischen Praxis möglichst effizient und ökonomisch zu gestalten.

5.1 Methodische Vorgehensweise

5.1.1 Rekrutierung der Patientenstichprobe und Definition von Ein- und Ausschlusskriterien

Die Patientenstichprobe soll deutschlandweit mit Unterstützung der Bezirksverwaltungen der BG Verkehr rekrutiert werden. Ausgehend von der Verfügbarkeit aller personellen und technischen Ressourcen ist anzunehmen, dass pro Monat zwei einwöchige Blocktherapien stattfinden können und damit eine Stichprobe in der angestrebten Größe von 2x10 Patienten innerhalb eines Jahres untersucht werden kann. Nach Angaben von Trauma-Lotsen der Bezirksverwaltung Dresden werden dort jährlich etwa 100 Kraftfahrer mit psychischen Problemen nach einem Arbeitsunfall gemeldet. Etwa ein Viertel davon benötigt eine stationäre oder zumindest eine ambulante Psychotherapie. Geht man für die übrigen Bezirksverwaltungen von vergleichbaren Zahlen aus, wären etwa 175 Fälle im Jahr rekrutierbar. Das Rekrutierungspotential spricht dafür, dass eine Studiendurchführung innerhalb eines Jahres realistisch ist.

Nach Meldung eines Patienten bei den BGen wird dieser von den Trauma-Lotsen über die Möglichkeit an einer Studienteilnahme informiert. Mit ihrer Erlaubnis werden die Patienten frühestens vier Wochen nach dem Unfall durch Mitarbeiter der Hochschulambulanz der Universität Würzburg telefonisch kontaktiert, um die Patienten genauer über die Studie zu informieren. Besteht weiterhin die Bereitschaft zur Teilnahme, erfolgt eine erste telefonische Abfrage der Ein- und Ausschlusskriterien und eine erste Diagnosestellung nach ICD-10. Um den von Garcia-Palacios et al. (2007) berichteten Befund zu replizieren, dass eine VRET gegenüber einer reinen In-Vivo-Exposition bevorzugt wird, sollen die Patienten im Rahmen des Screening-Telefonats befragt werden, welche Art der Exposition sie präferieren würden. Basierend auf der einschlägigen Fachliteratur und den Erfahrungen des IAG im Rahmen der eingangs angesprochenen Einzelfall-Sitzungen, werden die Ein- und Ausschlusskriterien zur Studienteilnahme so gewählt, dass nach bisherigem Kenntnisstand von einer hohen Wirksamkeit der Exposition auszugehen ist:

Einschlusskriterien:

- Berufskraftfahrer mit „Anpassungsstörung“, „spezifischer (isolierter) Phobie“ oder PTBS nach Arbeits-/Verkehrsunfall eines motorisierten Fahrzeugs mit einer frontalen/lateralen Kollision als Fahrer (Voraussetzung für visuelle Gefahrenreize)
- einhergehend mit Fahrangst/-vermeidung
- seit mindestens einem Monat
- keine bisherige Expositionstherapie
- keine oder seit mindestens vier Wochen stabile Psychopharmaka-Therapie
- Fahrerlaubnis und regelmäßige Fahrpraxis vor dem Unfall
- im Altersbereich 18-63

Ausschlusskriterien:

- Substanzabhängigkeit
- Suizidalität
- Psychosen u.a. (prämorbide) psychische Störungen und Komorbiditäten wie koronare Herzkrankheiten und Epilepsie sowie andere Befunde, die im Rahmen der Konsiliaruntersuchung am medizinischen Studienzentrum Würzburg festgestellt werden und nach Ermessen des untersuchenden Arztes möglicherweise eine Kontraindikation für eine Exposition darstellen

- Schwere kognitive Beeinträchtigungen (Score < 27 im Mini Mental Status Test nach Folstein et al., 1975)
- Nur für Frauen: positiver Schwangerschaftstest.

Sind alle Einschlusskriterien und keines der Ausschlusskriterien nach erster Einschätzung erfüllt, werden die Patienten per Zufall einer der beiden Gruppen (Behandlungs- vs. Wartegruppe) zugeteilt und zur ausführlichen Untersuchung und Behandlung nach Würzburg eingeladen.

Die Fahrvermeidung wird zunächst subjektiv durch ein hypothetisches Angebot zu einer Fahrprobe erfragt, unmittelbar vor der Therapie dann aber durch ein tatsächliches Angebot dazu auf der Verhaltensebene bestätigt. Dementsprechend sollte eine zu Beginn der Behandlung angebotene Fahrprobe mit Fahrlehrer verweigert oder nur mit großer Angst und auffälligem Fahrverhalten[1] absolviert werden (sog. Behavioral Avoidance Test, BAT).

Die Randomisierung erfolgt durch ein Losverfahren. Jeder Patient, mit dem die Hochschulambulanz Kontakt aufnimmt, erhält zunächst fortlaufend eine Screeningnummer beginnend mit 1001. Jede Person, die gemäß dieser telefonischen Einschätzung des Psychotherapeuten die Einschlusskriterien erfüllt und der Studienteilnahme zustimmt, erhält fortlaufend eine Randomisierungsnummer beginnend mit 101. Die Zuteilung zu den beiden Gruppen wird per Los entschieden. Dazu werden zehn Lose mit der Aufschrift „Behandlungsgruppe“ und zehn Lose mit der Aufschrift „Wartegruppe“ verschlossen und in einer Losbox aufbewahrt. Am Ende des Screeningtelefonats und nach Zuteilung der Randomisierungsnummer zieht der Therapeut ein Los, um mit dem Patienten die (für die beiden Gruppen unterschiedlichen) Termine vereinbaren zu können. Screeningnummer, Randomisierungsnummer sowie die geloste Gruppenzugehörigkeit werden in einer Liste dokumentiert. Dropouts werden ersetzt, indem der Losbox ein weiteres Los mit der jeweiligen Gruppe hinzugefügt wird.

5.1.2 Ablauf der Behandlung

Der Ablauf der einwöchigen Blockbehandlung ist getrennt für die Behandlungs- und Wartegruppe in Tabelle 1 dargestellt. Die Zuteilung der Patienten zu den beiden Gruppen erfolgt per Zufall (s. vorheriger Abschnitt).

Die Behandlung startet für beide Gruppen jeweils nach erfolgtem Screeningtelefonat an einem Montagvormittag mit dem Empfang durch den behandelnden Psychotherapeuten in der Hochschulambulanz. Dort werden die Patienten zunächst nochmals ausführlich *persönlich* über die Studie informiert und um ihr schriftliches Einverständnis zur Teilnahme gebeten (Informed Consent). Ebenso wird nochmals die Diagnose einer „Anpassungsstörung“, einer „spezifischen (isolierten) Phobie“ oder einer PTBS nach ICD-10 gesichert. Danach erfolgt die ärztliche Konsiliaruntersuchung durch einen Prüfarzt am Medizinischen Studienzentrum Würzburg, das für diese Leistung unterbeauftragt wird. Der Prüfarzt überprüft insbesondere mögliche Kontraindikationen für eine Exposition im Rahmen einer medizinisch-neurologischen Screeninguntersuchung. Konkret sollen dazu eine ausführliche medizinische Anamnese inkl. psychiatrischem Befund und Screening nach psychiatrischen Erkrankungen (inkl. Suizidalität, Substanzabhängigkeit), eine körperliche (internistisch-neurologische) Untersuchung, EKG, EEG und ein Drogenscreening (sowie bei Frauen ein Schwangerschaftstest) durchgeführt werden. Zum Ausschluss schwerer kognitiver Beeinträchtigungen wird die MMSE (Folstein et al., 1975) angewandt.

[1] Für eine Operationalisierung eines „auffälligen Fahrverhaltens“ s. Abschnitt 5.1.4.

Sind alle Einschlusskriterien verifiziert und liegt kein Ausschlusskriterium vor, werden die Patienten wieder an die Psychotherapeuten der Hochschulambulanz der Universität Würzburg verwiesen. Direkt im Anschluss startet dann die Behandlung. Dabei wird sich der Psychotherapeut an ein Therapiemanual halten, das zu Beginn des Projekts in Anlehnung an einschlägige Therapiemanuale der Fachliteratur (u.a. Hamm, 2006, König, 2012, Zöllner et al., 2005) erarbeitet werden wird (s. dazu auch Abschnitt 5.5).

Die Behandlung beginnt mit einem psychotherapeutischen Anamnese-Gespräch mit folgenden Inhalten:

- Grobe Erfassung des Unfallgeschehens (mit Hilfe des Motor Vehicle Accident Interview nach Blanchard & Hickling, 2004)
- Sammlung von Symptomen, vermiedenen Kognitionen und besonders kritischen Fahrsituationen („Hot Spots“)
- nochmalige Erläuterung des Behandlungsablaufs
- Vorgabe von verschiedenen Fragebögen zu Ängsten und Depression
- Erfassung von Konzentrationsschwierigkeiten (d2-Test nach Brickenkamp et al., 2010).

Als Hinweise auf operante Faktoren sollen im Rahmen der Anamnese-Sitzung[2] auch die Kriterien Teilnahmemotivation, Arbeitsplatzbelastung und psychosoziale Stressoren mit Hilfe der folgenden Verfahren standardisiert erfasst werden:

- AVEM (Arbeitsbezogenes Verhaltens- und Erlebensmuster von Schaarschmidt & Fischer, 2003)
- FPTM (Fragebogen zur Psychotherapiemotivation von Nübling et al., 2002)
- Social Readjustment Rating Scale (Holmes & Rahe, 1967)
- ABF (Alltagsbelastungsfragebogen von Traue et al., 2000).

[2] Falls dies im Rahmen der Anamnese-Sitzung aus zeitlichen Gründen oder einer zu starken Belastung der Patienten nicht mehr zu leisten ist, können die Verfahren auch an Tag 2 (Behandlungsgruppe) bzw. Tag 9 (Wartegruppe) durchgeführt werden.

Tabelle 1: Behandlungsablauf für Patienten der Behandlungs- (BG) und der Wartegruppe (WG). PT-Sitzung = Psychotherapiesitzung; Sim=Simulatorsitzung.

	Tag –x:	Tag 1: Mo	Tag 2: Di	Tag 3: Mi	Tag 4: Do	Tag 5: Fr	Tag 8: Mo	Tag 9-12: Di-Fr	+1.5/3 Monate
BG	Screening-telefonat	Med. Konsiliar-unter-suchung<br><br>Psychoth. Anamnese Fahrprobe m. Fahr-lehrer	PT-Sitzung m. Schreck-reflex-messung	PT-Sitzung	Sim3 (mittlerer Hierarchie - bereich)	Sim5 (mittlerer/ oberer Hierarchie-bereich)			Telef. Booster-Sitzung / Follow-up
			Sim1 (nur Kennen-lernen)	Sim2 (unterer Hierarchie-bereich)	Sim4 (oberer Hierarchie-bereich)	Fahrprobe m./o. Fahrlehrer<br><br>Nachbespr. m. Schreck-reflex-messung			
WG	Screening-telefonat	Med. Konsiliar-unters-uchung<br><br>Psychoth. Anamnese hypoth. Angebot zu Fahr-probe m. Fahrlehrer						wie BG	Telef. Booster-Sitzung / Follow-up
							Fahrpro-be m. Fahr-lehrer	wie BG	

Im Anschluss an das Anamnesegespräch wird den Patienten der Behandlungsgruppe angeboten, eine kurze Strecke in einem Fahrschulauto begleitet vom Psychotherapeuten, einem Fahrlehrer und einem Verkehrspsychologen des WIVW zu fahren. Wie bereits erläutert wird ein Patient in die Studie nur dann aufgenommen, wenn diese Fahrprobe (Behavior Avoidance Test, BAT) verweigert bzw. mit (laut Fahrlehrer- und Verkehrspsychologenurteil) sehr auffälligem und ängstlichem Fahrverhalten absolviert wird. Für die Teilnehmer der Wartegruppe, die als Kontrollgruppe dient, wird nach dem Anamnesegespräch die Behandlung für eine Woche unterbrochen. Der Wartegruppe wird die Fahrprobe nur hypothetisch mit folgenden Fragen durch den Therapeuten am Ende des Anamnesegesprächs angeboten:

1. Wenn wir Ihnen nun anbieten würden, eine kurze Strecke in Begleitung (Psychotherapeut und Fahrlehrer) zu fahren, würden Sie das tun? Ja - Nein
2. Wenn ja, wie belastend wäre das für Sie? Antwort auf 16-stufiger Numeralskala mit den verbalen Oberkategorien (gar nicht - sehr wenig – wenig – mittel – stark –sehr stark)

Eingeschlossen werden nur

- Patienten, die auf 1. mit Nein antworten oder
- Patienten, die auf 1. mit Ja antworten, aber bei 2. mindestens „stark“ angeben.

Für die kommende Woche werden die Patienten der Wartegruppe dann erneut eingeladen um die Behandlung ebenfalls erhalten zu können. Dazu wird ihnen am darauffolgenden Montag zunächst die Fahrprobe erneut angeboten, im Falle einer positiven Beantwortung („ja, würde ich machen“) dann aber auch tatsächlich durchgeführt (analog zu dem Vorgehen bei der Behandlungsgruppe). Ebenso werden die in der Anamnese vorgegebenen Fragebögen nochmals erhoben. Nur wenn der BAT verweigert bzw. mit sehr auffälligem und ängstlichem Fahrver-

halten absolviert wurde, wird die VRET-Behandlung am Dienstagvormittag äquivalent zur Behandlungsgruppe begonnen,

Um potentiellen Symptomverschlimmerungen nach dem BAT oder allgemeinen Krisensituationen in der Zeit der Studiendurchführung entgegenzuwirken, wird der Psychotherapeut den Patienten nach dem BAT zu einem therapeutischen Anschlussgespräch zur Verfügung stehen. Weiterhin erhalten alle Patienten eine Notfallnummer, unter der sie einen Psychotherapeuten der Hochschulambulanz telefonisch erreichen können.

Die eigentliche Behandlung beginnt am Dienstagvormittag mit einer Psychotherapie-Sitzung und den folgenden Inhalten:

- Darstellung eines Erklärungsmodells zur Angststörung mit Erarbeitung einer Vermeidungshierarchie von Fahrszenarien
- Vermittlung des Behandlungsrationals (Erläuterung inwiefern eine Exposition helfen kann, die Angst zu bewältigen)
- Verfassen eines Unfallberichts im Beisein des Therapeuten

Als physiologisches Maß für die Ausprägung der Fahrangst soll in dieser Sitzung der sog. „Schreckreflex“ erhoben werden. Es handelt sich dabei um eine Muskelkontraktion am Augenlid, die mittels Elektromyogramm am Musculus orbicularis oculi abgeleitet wird. Ausgelöst wird die Reaktion durch jeweils sechs unangenehme auditive Reize in einer neutralen Situation und während einer Lesekonfrontation mit dem selbstverfassten Unfallbericht. Dabei wird angenommen, dass die Reaktion während der Lesekonfrontation stärker ist als während der neutralen Situation und dass dieser Unterschied am Ende der Therapie geringer ist als zu Beginn. Außerdem sollen die Patienten die Stärke ihrer Angst in beiden Situationen selbst einschätzen (unter Verwendung der 10-stufigen Subjective-Unit-of-Distress-Skala, Abgabe sog. „SUD-Ratings“).

Im Anschluss an die Therapiesitzung wird die erarbeitete Hierarchie von Fahrszenarien vom Psychotherapeuten mit einem Verkehrspsychologen und einem Szenarioentwickler des WIVW besprochen, damit dieser entsprechende Szenarien für die Simulation programmieren kann. Ziel ist es, eine hierarchisch geordnete Reihe von kurzen Szenarien für jeden Patienten individuell so zu gestalten, dass die Angst vor dem Durchfahren eines Szenarios mit der jeweiligen Hierarchiestufe ansteigt und in einem am stärksten vermiedenen Szenario gipfelt. Dies wird gewährleistet, indem zunehmend mehr sog. „Hot Spots“ (angstauslösende Reize), die im therapeutischen Gespräch identifiziert wurden, dargeboten werden (z.B. Kreuzung mit normalem Querverkehr, Kreuzung mit Einsatzfahrzeugen im Querverkehr, Einsatzfahrzeuge im Längsverkehr, Passieren einer Unfallstelle mit Einsatzfahrzeugen). Ggf. kann hier auf die vom IAG verwendeten Expositionsstrecken zurückgegriffen werden, die dort im Rahmen der eingangs berichteten Einzelfall-Sitzungen programmiert wurden, da sowohl der dortige als auch der für das geplante Vorhaben eingesetzte Simulator des WIVW mit der Software SILAB betrieben werden.

Am Dienstagnachmittag erhalten die Patienten die Gelegenheit, den Simulator zu besichtigen und kennenzulernen (s. Abbildung 1). Sie können sich in die Fahrzeugkabine setzen und – wenn sie möchten - auch eine kurze Probefahrt auf einem reizarmen Parkplatz und einer einfachen Landstraße durchführen. Eine Exposition gegenüber der mit dem Therapeuten erarbeiteten Hierarchie von Fahrszenarien soll an diesem Tag bewusst noch nicht stattfinden.

Vor, während und nach diesem Besichtigungstermin wird die von den Patienten empfundene Angst wieder subjektiv über die SUD-Skala erfasst. Unter Verwendung halbstandardisierter Beobachtungsbögen registriert der Psychotherapeut zudem das Vermeidungsverhalten der Patienten. Erklärt sich ein Patient dazu bereit, zu fahren, wird sein Fahrverhalten von einem

geschulten Verkehrspsychologen mit Hilfe einer vom WIVW entwickelten Applikation für Tablet-PCs zur Fahrverhaltensbeobachtung beurteilt (Standardized Application for Fitness to Drive Evaluations S.A.F.E., s. auch Abbildung 1). Erfahrungsgemäß fahren allerdings auch gesunde Probanden die ersten Strecken im Simulator oft sehr vorsichtig und langsam. Dies ist bei der Bewertung zu berücksichtigen. Allerdings wurden bei den Sitzungen mit PTBS-Patienten am IAG extrem auffällige Verhaltensweisen beobachtet (wie bspw. eine Maximalgeschwindigkeit von unter 10km/h), was entsprechend zu registrieren ist. Da insbesondere die ersten Fahrten in der Fahrsimulation mit Übelkeit einhergehen können, wird das Auftreten von „Simulator Sickness“ durch einen Fragebogen zu körperlichen Beschwerden in Anlehnung an Kennedy et al. (1993) kontrolliert.

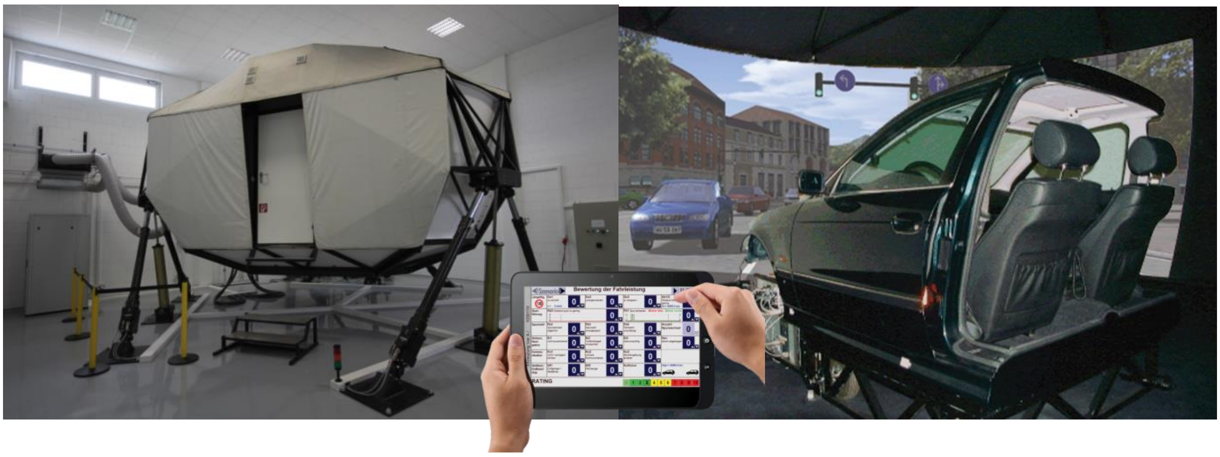

Abbildung 1: Der Fahrsimulator des Würzburger Instituts für Verkehrswissenschaften mit Bewegungssystem, in dem die Expositionstherapie stattfinden soll. Er besteht aus einem voll instrumentierten seriengefertigten BMW 520i mit Automatikgetriebe (rechts), der sich in einem Dom (links) befindet. Das Sichtfeld beträgt 180° und wird über drei Projektoren auf eine sphärische Leinwand projiziert. Sowohl die Außen- als auch der Innenspiegel werden durch LCD-Monitore simuliert. Der Psychotherapeut wird sich in einem benachbarten Operatorraum aufhalten und den Patienten während der Fahrten per Mikrophon betreuen. Mittels verschiedener Monitore sind für den Psychotherapeuten der Fahrer selbst, das aktuell durchfahrene Fahrszenario sowie aktuelle Fahrdaten in Echtzeit zu sehen. Mittels einer teilautomatisierten Applikation für Tablet-PCs (Mitte) wird ein geschulter Verkehrspsychologe Fahrfehler und Auffälligkeiten im Fahrverhalten registrieren (Standardized Application for Fitness to Drive Evaluations, S.A.F.E.).

Der Mittwochvormittag startet mit einer Psychotherapie-Sitzung, in der die Besichtigung des Simulators nachbesprochen wird und die Patienten auf die für den Nachmittag vorgesehene Exposition in der Simulation vorbereitet werden. Im Rahmen von insgesamt drei Expositionssitzungen wird angestrebt, die hierarchische Abfolge von Fahrszenarien, wie sie mit dem Therapeuten erarbeitet und in die Simulation implementiert wurden, sukzessive zu durchfahren. Idealerweise wäre dies am Mittwochnachmittag der untere, am Donnerstagvormittag der mittlere und am Donnerstagnachmittag der obere Hierarchiebereich. Spontane und an den individuellen Patienten angepasste Abweichungen, die nach Ermessen des Therapeuten sinnvoll sind, dürfen und sollen hier aber durchaus berücksichtigt und umgesetzt werden. Bei der Gestaltung der Strecken ist zudem zu berücksichtigen, dass eine langsame Gewöhnung an das Fahren im Simulator stattfinden muss, indem das Ausmaß notwendiger Brems-, Beschleunigungs- und Lenkmanöver allmählich ansteigt. Diese Form der Simulatorgewöhnung wird am WIVW standardmäßig durchgeführt und dient einerseits dazu, ein natürliches Fahrverhalten herzustellen und andererseits das Risiko einer Simulator Sickness erheblich zu vermindern (s. Hoffmann & Buld, 2006). Vor jeder Expositionssitzung werden die geplanten und zwischen-

zeitlich programmierten Strecken dem Psychotherapeuten durch einen Verkehrpsychologen präsentiert und das genaue Vorgehen sowie die Formulierung von Instruktionen vor und während der Fahrten gemeinsam besprochen. Kleinere Anpassungen sind auch zu diesem Zeitpunkt noch möglich. Während der Expositionssitzung werden die subjektiv empfundene Angst, das Vermeidungs- und Fahrverhalten sowie Symptome der Simulator Sickness erfasst. Abbildung 2 zeigt beispielhafte Szenarien wie sie für den unteren und oberen Bereich einer solchen Hierarchie denkbar wären.

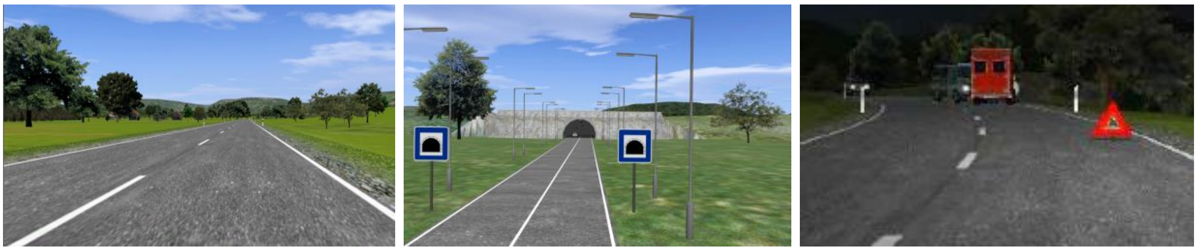

Abbildung 2: Beispielhafte Szenarien der Software SILAB für den unteren (links: einer einfachen Landstraße folgen) und oberen (Mitte: Tunnel durchfahren, rechts: Unfallstelle bei Nacht mit Einsatzfahrzeugen passieren) Bereich individuell gestalteter Hierarchien zur Exposition in der Fahrsimulation.

In einer letzten Expositionssitzung soll schließlich am Freitagvormittag die Wirksamkeit der VRET-Behandlung am einzelnen Patienten überprüft werden, in dem jeweils eine Strecke aus dem mittleren und dem oberen Bereich der eigenen Hierarchie in der Simulation nochmals durchfahren wird. Dazu werden wiederum die subjektiv empfundene Angst (SUD-Ratings) erfasst, das Vermeidungs- sowie das Fahrverhalten beurteilt und Symptome der Simulator Sickness kontrolliert.

Als objektiver Indikator für die psychische Belastung soll bei allen Expositionsitzungen in der Simulation der Puls der Probanden erfasst werden. Konkret soll das Pulssignal zu Beginn der Expositionen mit einer Baselinemessung (nach der Exposition während einer vom Therapeuten angeleiteten kurzen Entspannung) verglichen werden. Ausgewertet werden soll schließlich die baseline-korrigierte Veränderung zwischen und innerhalb der Sitzungen.

Im psychotherapeutischen Abschlussgespräch am Freitagnachmittag werden die Therapieinhalte zusammengefasst, potentielle Belastungen im Alltag eruriert, welche die Aufrechterhaltung des Behandlungserfolgs erschweren könnten, sowie Möglichkeiten zur Rückfallprophylaxe und ein Übungsplan für die nächsten Wochen erarbeitet. Ebenso wird auf die Notwendigkeit weiterer Therapiesitzungen und –inhalte mit einem ambulanten Therapeuten vor Ort hingewiesen. Im Rahmen dieses Abschlussgesprächs wird auch der d2-Test (Brickenkamp et al., 2010) im Hinblick auf eine mögliche Besserung von Konzentrationsschwierigkeiten wiederholt. Gleiches gilt für die Messung des Schreckreflexes während einer neutralen Situation und einer Lesekonfrontation gegenüber dem eigenen Unfallbericht, um die Wirksamkeit der Therapie anhand dieses physiologischen Indikators zu bewerten. Ebenso werden dabei die SUD-Ratings als subjektives Maß ein weiteres Mal erhoben.

Das entscheidende Kriterium für die Bewertung des Therapieerfolgs ist aber, ob nach der Therapie eine Fahrverhaltensprobe mit Fahrlehrer, Psychotherapeut und Verkehrspsychologe mit angemessenem Fahrverhalten und verminderter Angst absolviert werden kann oder nicht. Die Beurteilung erfolgt auf mehreren Ebenen anhand des Urteils von Fahrlehrer und Verkehrspsychologe zum Fahrverhalten (unterstützt durch S.A.F.E.), anhand des Urteils des Psychotherapeuten zum Vermeidungsverhalten (halbstandardisierter Beobachtungsbogen) und subjektiv durch die Skalierung der Angst durch die Patienten selbst (SUD-Ratings). Verläuft diese Fahrprobe erfolgreich, werden die Patienten zum Abschluss zusätzlich darum gebeten, eine kurze Fahrt mit einem Versuchsträger des WIVW alleine zu unternehmen, um zu prüfen,

ob der Erfolg nicht nur auf die Anwesenheit von Fahrlehrer oder Psychologen zurückzuführen ist. Ist der BAT für die Patienten noch immer mit einer starken psychischen Belastung verbunden, steht der Psychotherapeut im Anschluss zu einer therapeutischen Nachbereitung zur Verfügung.

Nach der einwöchigen Behandlung werden die Patienten durch die zuständige BG an ihren (bzw. falls noch nicht geschehen an einen) ambulant tätigen Therapeuten in der Nähe ihres Wohnorts überwiesen. Sechs Wochen nach Ende der Behandlung soll aber noch eine telefonische „Booster-Sitzung" mit dem Studientherapeuten stattfinden, in deren Rahmen die Patienten zu schwierigen Situationen und ihrem Umgang damit befragt werden und die wichtigsten therapeutischen Erkenntnisse nochmals aufgefrischt werden. Den Studienabschluss bildet schließlich eine Follow-up-Untersuchung drei Monate nach Ende der Behandlung, bei der zum einen die verschiedenen standardisierten und einschlägigen Fragebögen zu Ängsten und Depression, die bereits in der Anamnese vorgelegt wurden, erneut erhoben werden und zum anderen in einem diagnostischen Interview erfasst wird, wie stark die Fahrangst und das damit verbundene Vermeidungsverhalten wieder oder noch ausgeprägt ist. Schließlich soll die gesamte Behandlung durch die Patienten evaluiert werden.

5.1.3 Maße zur Beurteilung der Wirksamkeit (Endpunkte)

Wie der Darstellung des Studienablaufs zu entnehmen ist, werden im Verlauf der Studie zahlreiche Endpunkte zur Beurteilung der Wirksamkeit der Behandlung auf subjektiver, physiologischer und behavioraler Ebene erhoben. Zur besseren Übersicht sollen diese hier (zusammen mit den Zeitpunkten ihrer Erhebung und den dafür angedachten Methoden) nochmals detailliert aufgelistet werden:

Primärer Endpunkt:

- Fahrleistung/-verhalten bei der Fahrprobe im Realverkehr nach und ggf. auch vor der Behandlung anhand von Fahrlehrerurteil sowie Verkehrspsychologenurteil unterstützt durch S.A.F.E. (Operationalisierung und Umgang mit Inkonsistenzen s. Abschnitt 5.1.4)

Sekundäre Endpunkte

- Vermeidungsverhalten bei der Fahrverhaltensprobe im Realverkehr vor und nach der Behandlung mit halbstand. Beobachtungsbogen der Hochschulambulanz
- Subjektive Angst bei den Fahrverhaltensproben nach und (falls Patient trotz seiner Fahrangst fährt) vor der Behandlung sowie im Verlauf der Simulatorsitzungen mittels SUD-Skala
- Schreckreflex (mittlere Reaktion auf je sechs akustische Signale) vor und nach der Behandlung mit EMG-Ableitung während neutraler Situation und einer Lesekonfrontation gegenüber dem eigenen Unfallbericht
- Konzentrationsfähigkeit mittels d2-Test (Brickenkamp et al., 2010) vor und nach der Behandlung
- Baseline-korrigierte Veränderung des Pulses zwischen und innerhalb der Expositionssitzungen
- Unfallangst vor der Behandlung und im Follow-up mittels „Accident Fear Questionnaire, AFQ" (Kuch et al., 1995)
- Posttraumatic Stress Scale Self-Report PSS-SR (Foa et al., 1997) vor der Behandlung und im Follow-up

- Symptome von Angststörungen und Depressionen im Allgemeinen vor der Behandlung und im Follow-up mittels „Beck Anxiety & Depression Inventory“ (Beck, Steer, & Brown, 1996 bzw. Beck & Steer, 1993)
- Fahr- und Vermeidungsverhalten im Simulator während der Simulatorsitzungen mittels Verkehrspsychologenurteil unterstützt durch S.A.F.E. und Therapeutenurteil anhand von halbstandardisiertem Beobachtungsbogen
- Simulator Sickness vor und nach jeder Simulatorstrecke mittels Symptomliste in Anlehnung an Kennedy et al. (1993).

5.1.4 Operationalisierung des Endpunkts Fahrleistung und Definition von Respondern vs. Non-Respondern

Alle Patienten werden basierend auf dem Fahrlehrer-/Verkehrspsychologenurteil zur Fahrverhaltensprobe an Tag 5 (Behandlungsgruppe) bzw. Tag 8 (Wartegruppe) in Responder und Non-Responder unterteilt. Das *Fahrlehrerurteil* soll auf einer 4-kategorialen Skala operationalisiert werden, wie sie von Brenner-Hartmann (2002) für standardisierte Fahrverhaltensbeobachtungen im Rahmen medizinisch-psychologischer Untersuchungen beschrieben wird.

Verbalkategorie	Numeralkategorie
absolut fahrunsicher	10
kritisches Fahrverhalten	9
	8
	7
auffälliges Fahrverhalten	6
	5
	4
normales Fahrverhalten	3
	2
	1
ohne Einschränkung fahrsicher	0

Abbildung 3: Fitness-to-Drive-Skala nach Neukum & Krüger (2003).

Demnach stuft der Fahrlehrer am Ende der Fahrt seinen Gesamteindruck auf der Skala mit den vier Ausprägungen „keine – vereinzelte – nicht unerhebliche – schwerwiegende Auffälligkeiten“ ein. Entsprechend der Ausführungen von Brenner-Hartmann (2002) soll der Übergang von vereinzelten zu nicht unerheblichen Auffälligkeiten als Cut für ein angemessenes Fahrverhalten gelten. Das Urteil des *Verkehrspsychologen* attestiert (entsprechend der Ausführungen in der Vorhabensbeschreibung) dann ein angemessenes Fahrverhalten, wenn (am Ende der Fahrt) auf der 10-stufigen Fitness-to-Drive-Skala in Anlehnung an Neukum & Krüger (2003; s. Abbildung 3) maximal ein Wert von 3 vergeben wird. Da die Verblindung gegenüber der Gruppenzugehörigkeit für den Fahrlehrer leichter zu realisieren ist[3], soll seinem Urteil im Falle von Inkonsistenzen Priorität gegeben werden. Konkret soll mit Inkonsistenzen wie folgt umgegangen werden:

- Attestiert der Fahrlehrer nicht unerhebliche oder schwerwiegende Auffälligkeiten, gilt das Fahrverhalten als unangemessen unabhängig vom Urteil des Psychologen.
- Attestiert der Fahrlehrer allenfalls leichte Auffälligkeiten, der Psychologe aber ein kritisches Fahrverhalten (Werte 7-10 auf der FtD-Skala), gilt das Fahrverhalten als unangemessen.

[3] Die Therapie wird in den Räumen des WIVW stattfinden, so dass der Verkehrspsychologe Patienten der Behandlungsgruppe mit einer höheren Wahrscheinlichkeit begegnet als Patienten der Wartegruppe. Mögliche Kontakte zwischen Patienten und WIVW Mitarbeitern werden aber minimiert und es wird in jedem Fall gewährleistet, dass der urteilende Psychologe bei den Fahrverhaltensproben weder an den Therapiesitzungen beteiligt, noch über den exakten Studienablauf informiert ist (insbesondere darüber, dass die Fahrprobe am Ende der Behandlungs- bzw. Wartephase für die beiden Gruppen an unterschiedlichen Wochentagen stattfindet). Selbstverständlich wird auch der Fahrlehrer nicht über die unterschiedliche Terminierung der Gruppen informiert. Zur Aufrechterhaltung der Verblindung werden die Patienten ferner darum gebeten, während der Fahrverhaltensproben möglichst wenig zu sprechen.

- Attestiert der Fahrlehrer allenfalls leichte Auffälligkeiten, gilt das Fahrverhalten auch dann noch als angemessen, wenn der Psychologe ein auffälliges Fahrverhalten angibt (Werte 4-6 auf der Fitness-to-Drive-Skala).

5.1.5 Festlegung von Erfolgs- und Abbruchkriterien mit Erarbeitung von Forschungsfragen für ein Anschlussprojekt

Die Behandlung kann jederzeit auf Wunsch des Patienten abgebrochen werden. Da es sich bei dem geplanten Vorhaben um eine Pilotstudie mit einer geringen Fallzahl handelt, soll bei der Entscheidung für eine Weiterführung kein konfirmatorisch nachgewiesener Unterschied zwischen Behandlungs- und Wartegruppe bezüglich der Anzahl von Respondern vorausgesetzt werden. Deskriptiv sollte aber der Anteil an Respondern (Patienten, die die Fahrprobe mit Fahrlehrer nach einer Woche mit vs. ohne Behandlung *vollständig*, mit *angemessenen Fahrverhalten* absolvieren, s. Abschnitt 5.1.4) in der Behandlungsgruppe deutlich höher sein als in der Wartegruppe. In Anlehnung an Schwarzer & Schumacher (2007), die für gleichwertige Responderquoten in klinischen Therapiestudien eine maximale Abweichung von 20% nennen, soll die Wirksamkeit der hier vorgestellten Therapie als nachgewiesen gelten und die Beantragung eines Anschlussprojekts rechtfertigen, wenn der Anteil an Respondern

- in der Wartegruppe an Tag 8 maximal 30% ($n \leq 3$) beträgt *und*
- in der Behandlungsgruppe an Tag 5 mindestens 60% ($n \geq 6$) beträgt und somit mindestens doppelt so hoch ist.

Eine Responderrate von mindestens 60% in der Wartegruppe an Tag 12 (also nach der nachträglichen Behandlung) wird angestrebt, aber nicht vorausgesetzt.

Als sekundäre Wirksamkeitskriterien sollen abnehmende SUD-Ratings, abnehmende Pulswerte innerhalb und zwischen den Sitzungen sowie ein verminderter Schreckreflex bei der Lesekonfrontation gelten. Nicht als Erfolgskriterien herangezogen, aber explorativ bewertet werden sollen das vom Psychotherapeuten dokumentierte Vermeidungsverhalten beim BAT, die Veränderung der Konzentrationsleistung im d2-Test, die zu Behandlungsbeginn und im Follow-up erfassten Daten zum Fahrverhalten, zur Unfallangst sowie zu Symptomen von Depression, PTBS und Angststörungen im Allgemeinen (Beck Inventories, PSS-SR, AFQ).

Die Forschungsfragen für das geplante Anschlussprojekt werden sicher erst nach Durchführung der Pilotstudie konkret zu formulieren sein. Beispielhaft denkbar wären aber folgende Fragestellungen:

- Welche Merkmale der Patienten oder des vorangegangen Unfalls prädizieren einen hohen Therapieerfolg?
- Wie sollte eine Therapie mit dem Baustein „Virtuelle Exposition in der Fahrsimulation" optimal gestaltet werden?
- Wie wirksam ist eine solche Therapie im Vergleich zu einer klassischen In-Vivo-Therapie?
- Welche Mindestausstattung ist für einen Fahrsimulator zum expositionstherapeutischen Einsatz vorauszusetzen?
- Wie stark müssen die in der Exposition dargebotenen Fahrszenarien individualisiert programmiert werden? Inwieweit kann auf die im Proof of Concept entwickelten Szenarien i.S. eines Katalogs zurückgegriffen werden?

5.2 Aufgabenverteilung innerhalb der Forschungskooperation

Die genaue Aufgabenverteilung ist den Abschnitten 5.1.2 und 5.5 zu entnehmen. Grundsätzlich werden alle psychotherapeutischen Arbeiten von der Hochschulambulanz übernommen. Das WIVW ist für die Projektkoordination, die Programmierung und Bedienung der Fahrsimulation sowie die Beurteilung des Fahrverhaltens der Patienten verantwortlich.

5.3 Maßnahmen zur Qualitätssicherung

Alle Studien am WIVW werden zur Qualitätssicherung nach einem etablierten SOP-System durchgeführt, das im Jahr 2008 eingeführt wurde. Insbesondere sind SOPs speziell zu klinischen Studien enthalten, die in Anlehnung an ICH/GCP entwickelt wurden und somit deren Einhaltung gewährleisten. Grundsätzlich beachtet wird auch die Deklaration des Weltärztebundes von Helsinki in ihrer letzten Version (Fortaleza, 2013).

5.4 Einbezug von Zielgruppen und eines Forschungsbegleitkreises

Das Vorhaben soll an einer Subgruppe der späteren Zielgruppe durchgeführt und evaluiert werden: Berufskraftfahrer mit starker Fahrangst infolge eines Arbeitsunfalls (s. Abschnitt 5.1.1). Ein Forschungsbegleitkreis ist vorerst nicht vorgesehen. Mitarbeiter des IAG sollen aber miteinbezogen werden, da sie aufgrund der eingangs berichteten probatorisch durchgeführten Expositionen im dortigen Fahrsimulator äußerst erfolgversprechende Erfahrungen gemacht und das Vorhaben initiiert haben. Die entsprechenden Erfahrungen waren bereits für die Planung des Vorhabens sehr hilfreich (u.a. im Hinblick auf die Definition von Ein- und Ausschlusskriterien) und werden dies sicher auch für die Studiendurchführung sein.

5.5 Detaillierter Arbeits- und Zeitplan mit Zuordnung der Partner

Das Forschungsvorhaben besteht aus vier Arbeitspaketen (AP), für deren Bearbeitung eine Laufzeit von 18 Monaten und ein Arbeitsvolumen von 15.71 Personenmonaten (8.80 Monate für das WIVW, 6.91 Monate für die Hochschulambulanz, HA[4]) veranschlagt werden. Die Gestaltung und Durchführung der Fahrverhaltensproben im Realverkehr werden im Unterauftrag mit der Fahrschule Kwiotek aus Würzburg erfolgen. Für die medizinische Konsiliaruntersuchung wird das Medizinische Studienzentrum Würzburg unter der Leitung der neurologischen Fachärzte Dr. med. Klein und Dr. med. Oehler unterbeauftragt. Beide Institutionen wurden aufgrund einer sehr erfolgreichen Zusammenarbeit mit dem WIVW in anderen Studien gewählt.

Die verschiedenen APs mit den zugehörigen Arbeitsschritten sollen im Folgenden kurz skizziert werden:

AP1: Studienvorbereitung (Personenmonate: 1 WIVW, 2 HA)

Als Grundlage für AP2 sind verschiedene Vorarbeiten nötig:

- Erstellung Therapiemanual durch HA, Review durch WIVW
- Erstellung Ethik-Antrag durch WIVW, Review durch HA
- Materialienzusammenstellung/-erstellung (fahrleistungsbezogene Frage-/Beobachtungsbögen) durch WIVW
- Materialienzusammenstellung/-erstellung (therapie-/symptombezogene Frage-/Beobachtungsbögen) durch HA

[4] Kosten für Reise und Unterkunft der Patienten sowie anteilig auch die Therapiedurchführung (10 Sitzungen pro Patient) werden von der BG Verkehr übernommen werden. Für die Zeitplanung ist zu berücksichtigen, dass für die Erstellung des Schlussberichts, der nicht zuwendungsfähig ist, 1.5 weitere Personenmonate zu veranschlagen sind, was in die kalkulierte Laufzeit von 18 Monaten nicht eingeht und diese entsprechend verlängert.

- Gestaltung und Testung einer Fahrverhaltensprobe im Realverkehr mit der Applikation S.A.F.E. zur standardisierten Fahrverhaltensbeobachtung durch WIVW

AP2: Studiendurchführung

AP2.1 Durchführung der Blocktherapie (Personenmonate: 2.46 WIVW, 3.66 HA[5])

Durchführung der Blocktherapie an 2x10 Patienten wie unter 5.1.2 beschrieben:

- Screening-Telefonat/Rekrutierung, Anamnese-Sitzung mit Angebot zu/Begleitung bei einer Fahrverhaltensprobe mit Fahrlehrer (prä-Messung), sieben psychotherapeutische Sitzungen (je fünf davon mit Exposition in der Fahrsimulation), eine psychotherapeutische Abschlusssitzung mit Nachbesprechung und Angebot zu/Begleitung bei einer Fahrverhaltensprobe mit Fahrlehrer (post-Messung), telefonische Booster-Sitzung und Follow-up-Befragung durch HA
- Verkehrspsychologische Begleitung zur Beurteilung der Fahrleistung bei den Fahrverhaltensproben im Realverkehr und den fünf Expositionssitzungen in der Fahrsimulation (inkl. Anwendung der Applikation S.A.F.E., Bedienung der Fahrsimulation) durch WIVW

AP2.2 Implementation der erarbeiteten Hierarchie von Fahrszenarien in die Fahrsimulation (Personenmonate: 1.5 WIVW)

Diese Programmierung der Strecken mit der Software SILAB kann frühestens nach den Anamnese-Sitzungen beginnen und wird dann parallel zur den ersten psychotherapeutischen Sitzungen durchgeführt. Die Möglichkeit zu weiteren notwendigen Anpassungen/Ergänzungen, die sich erst im Verlauf ergeben können, soll gegeben sein.

AP3: Analyse, Bewertung und Dissemination der Befunde (Personenmonate: 2.79 WIVW, 1 HA)

Im Anschluss an die Durchführung der Blocktherapie wird eine statistische Auswertung der erhobenen Parameter stattfinden, um die Wirksamkeit der Therapie zu beurteilen, worauf wiederum die Entscheidung zur Beantragung eines Anschlussprojekts basieren wird. Ein frühzeitiger Transfer der erarbeiteten Projektergebnisse soll durch Veröffentlichungen in Fachzeitschriften und Vorträgen auf Fachkongressen (DGPPN, DGVP/DGVM, Fit to Drive) erreicht werden. Demzufolge ergeben sich folgende Arbeitsschritte:

- Aufbereitung der Schreckreflex-Daten zur statistischen Weiterverarbeitung durch HA
- Eingabe und Synchronisation der Daten aus den verschiedenen Datenquellen (Fahrsimulation/S.A.F.E., Schreckreflex/Varioport, Puls, Frage- und Beobachtungsbögen) mit Erstellung entsprechender Dateneingabemasken durch WIVW
- Statistische Auswertung durch WIVW
- Erstellung Zwischenberichte durch WIVW
- Review Zwischenberichte durch HA
- Entscheidung über Anschlussprojekt mit Erarbeitung von *verkehrspsychologischen* Anpassungen, Fragestellungen und Design durch WIVW
- Entscheidung über Anschlussprojekt mit Erarbeitung von *psychotherapeutischen* Anpassungen, Fragestellungen und Design durch HA

[5] Wie bereits erläutert werden die Kosten der Hochschulambulanz für die Therapiedurchführung anteilig von der BG Verkehr übernommen. Diese sind in den 3.5 Personenmonaten nicht berücksichtigt.

- Präsentation der Befunde auf *verkehrspsychologischen* Tagungen durch WIVW
- Präsentation der Befunde auf *psychotherapeutischen* Tagungen durch HA

AP4: Projektmanagement und Projekttreffen (Personenmonate: 1.02 WIVW, 0.25 HA)

Da es sich bei der Pilotstudie um ein Verbundvorhaben mit Unterbeauftragungen handelt, werden durchgängig verschiedene Arbeiten zur Projektkoordination anfallen, die vom WIVW übernommen werden. Weiterhin werden mindestens fünf Projekttreffen stattfinden, um sich über das Vorhaben und dessen Verlauf auszutauschen (einmal vor, zweimal während und einmal nach der Daten-Erhebung, sowie einmal nach der Datenauswertung).

Tabelle 2: Anzahl Personenmonate (gerundet) pro Projektmonat für jedes Arbeitspaket. Personenmonate der Hochschulambulanz sind orange, Personenmonate des WIVW sind blau unterlegt.

Monat / AP	1	2	3	4	5	6	7	8	9	10	11	12	13	14	15	16	17	18	∑
1	0.5	0.5																	1.00
	1	1																	2.00
2.1			0.21	0.21	0.21	0.21	0.21	0.21	0.21	0.21	0.21	0.21	0.21	0.21					2.46
			0.31	31	31	31	31	31	31	31	31	31	31	0.31					3.66
2.2			0.13	0.13	0.13	0.13	0.13	0.13	0.13	0.13	0.13	0.13	0.13	0.13					1.50
3								0.5			0.5				0.5	0.5	0.5	0.29	2.79
								0.13			0.13				0.25	0.25	0.14	0.1	1.00
4	0.06	0.06	0.06	0.06	0.06	0.06	0.06	0.06	0.06	0.06	0.06	0.06	0.06	0.06	0.06	0.06	0.06	0.06	1.02
		0.05				0.05				0.05				0.05			0.05		0.25

6 ERWARTETE ERGEBNISSE

Es wird erwartet, dass mindestens 2/3 der Patienten mit Fahrangst nach einem Verkehrsunfall durch die hier beschriebene Therapieform erfolgreich behandelt werden können (s. Abschnitt 5.1.5). Wird diese Erwartung bestätigt, sollen in einer anschließend zu beantragenden größeren Evaluationsstudie prädiktive Patienten-/ Unfallmerkmale für eine solche Therapie identifiziert und eine optimale Therapiegestaltung herausgearbeitet werden, um schließlich auch einen Vergleich mit einer reinen In-Vivo-Therapie durchzuführen. Fernziel ist es, die entwickelte Therapieform in die therapeutische Praxis einzuführen (s. nächster Abschnitt).

7 PRAKTISCHE UMSETZUNG DER ERGEBNISSE

Bei dem beschriebenen Vorhaben handelt es sich um eine Pilotstudie, so dass direkt im Anschluss keine praktische Umsetzung der Ergebnisse zu erwarten ist. Vielmehr soll im Falle einer nachgewiesenen Wirksamkeit ein Anschlussprojekt beantragt werden, um die Grundlagen für eine Einführung der Behandlungsform in die therapeutische Praxis zu schaffen (s. auch Abschnitt 5.1.5). Längerfristiges Ziel ist es, das im Rahmen dieser Arbeiten entwickelte

Programm an mehreren Berufsgenossenschafts-, aber auch anderen psychotherapeutisch arbeitenden Kliniken anzubieten. Wie unter Abschnitt 4 erläutert, wäre die Durchführung der Therapie am Fahrsimulator am IAG der DGUV direkt möglich, da dieser ebenfalls mit der Software SILAB betrieben wird. Durch den Aufbau von Simulatoren in weiteren Berufsgenossenschafts- und anderen Kliniken soll in einem Zeithorizont von etwa 5 Jahren eine deutschlandweite Versorgung initiiert werden.

Im Hinblick auf Fragen zur Fahrtauglichkeit im Alter, unter Medikamenteinfluss und bei Erkrankungen (wie Demenz, M. Parkinson, Schlaganfall, u.v.a.) beginnt sich die Fahrsimulation schon jetzt als ein vielversprechendes Werkzeug zur Diagnose, aber auch zum Erhalt und zur Rehabilitation von Fahrtauglichkeit zu etablieren. Insbesondere weil hier spezifisch auf die Symptomatik zugeschnittene Szenarien standardisiert, reproduzierbar und gefahrfrei dargeboten werden und die Patienten bei einer hohen Augenscheinvalidität Leistungsbeeinträchtigungen wie im tatsächlichen Verkehr kompensieren können, wird die Simulation gegenüber den traditionellen Methoden der Fahreignungsdiagnostik als überlegen angesehen (s. Hartje, 2004 oder Kaussner, 2007). So haben bereits mehrere Kliniken beim WIVW ein großes Interesse am Aufbau von Fahrsimulatoren bekundet. Dazu zählen u.a. Kliniken, deren Beschäftigte dem Arbeitskreis Fahreignung der Gesellschaft für Neuropsychologie angegliedert sind und zu denen im Rahmen des Arbeitskreises langjährige Kontakte zum WIVW bestehen (Dr. Becker Klinikgesellschaft mbH & Co. KG, Kiliani-Klinik Bad Windsheim, Klinikum Karlsbad-Langensteinbach, Sachsenklinik, Evangelisches Geriatriezentrum Berlin GmbH/ Medizinische Fakultät der Humboldt-Universität zu Berlin (Charité), Campus Virchow-Klinikum, Bezirksklinikum Maikofen Deggendorf, Kliniken Schmieder). Die Liste der Kontaktkliniken, die an Fahrsimulatoren interessiert sind, wurde 2013 durch die Teilnahme des WIVW als Aussteller bei der Tagung der Deutschen Gesellschaft für Neurologie um 50 weitere ergänzt. Bereits heute besitzt das Inn-Salzach-Klinikum in Wasserburg am Inn einen Fahrsimulator, der mit der vom Antragsteller entwickelten Fahrsimulationssoftware SILAB, dem Szenarienpaket „Driver Fitness and Ability“ und der Applikation S.A.F.E. ausgestattet ist. Auch dort bestünden von Seiten der psychiatrischen und neurologischen Abteilung sicher Interessen an einer Erweiterung der Einsatzmöglichkeiten des Fahrsimulators um psychotherapeutische Maßnahmen.

8 LITERATUR

Beck, J. G., Palyo, S. A., Winer, E. H., Schwagler, B. E., & Ang, E. J. (2007). Virtual reality exposure therapy for PTSD symptoms after a road accident: An uncontrolled case series. Behavior Therapy, 38, 39-48.

Beck, A. T., & Steer, R. A. (1993). Manual for the Beck Depression Inventory. San Antonio, TX: Psychological Corporation.

Beck, A. T., Steer, R. A., & Brown, G. K. (1996). Manual for the Beck Depression Inventory - II. San Antonio, TX: Psychological Corporation.

Blanchard, E. B., & Hickling, H. J. (2004). What are the psychosocial effects of MVAs on survivors? In E. B. Blanchard, E. J. Hickling (Eds.), After the crash: Psychological assessment and treatment of survivors of motor vehicle accidents (2[a] ed., pp. 57-97).Washington, DC: American Psychological Association.

Brickenkamp, R., Schmidt-Atzert, L. & Liepmann, D. (2010). d2-R: Test d2 - Revision. Hogrefe, Göttingen.

Brenner-Hartmann, J. (2002). Durchführung standardisierter Fahrverhaltensbeobachtungen im Rahmen der medizinisch-psychologischen Untersuchung (MPU). Vortrag beim 38. BDP-Kongress für Verkehrspsychologie in Regensburg, 2002. http://www.bdp-verkehr.de/backstage2/ver/documentpool/kongress/brenner_01.pdf.

Emmelkamp, P.M., Krijn, M., Hulsbosch, A.M., deVries, S., Schuemie, M.J. & van der Mast, C.A. (2002). Virtual reality treatment versus exposure in vivo: a comparative evaluation in acrophobia. Behavior Research and Therapy. 40(5), 509-16.

Foa, E. B., & Kozak, M. J. (1986). Emotional processing of fear: Exposure to corrective information. Psychological Bulletin, 99, 20-35.

Foa, E.B., Cashman, L., Jaycox, L. & Perry, K. (1997). The validation of a self-report measure of posttraumatic stress disorder : the Posttraumatic Diagnostic Scale. In: Psychological Assessment, 9, 445–451.

Foa, E. B., & Meadows, E. A. (1997). Psychosocial treatments for posttraumatic stress disorder: A critical review. Annual Review of Psychology, 48, 449-480.

Folstein, J. F., Folstein, S. E., & McHugh, P. R. (1975). "Mini-mental state": A practical method for grading cognitive state of patients for the clinician. Journal of Psychiatric Research, 43, 882-885.

Garcia-Palacios, A., Botella, C., Hoffman, H. & Fabregat, S. (2007). Comparing Acceptance and Refusal Rates of Virtual Reality Exposure vs. In Vivo Exposure by Patients with Specific Phobias. CyberPsychology & Behavior, 10 (5), 722-724.

Garcia-Palacios, A., Hoffman, H., Carlin, A., Furness, T. A. III, & Botella, C. (2002). Virtual reality in the treatment of spider phobia: A controlled study. Behaviour Research and Therapy, 40, 983-993.

Hamm, A. (2006). Spezifische Phobien. Göttingen: Hogrefe.

Hartje, W. (2004). Die neuropsychologische Testdiagnostik hat nur begrenzte Aussagekraft für die Fahreignung. In: C. Dettmers & C. Weiller (Hrsg.), Fahreignung bei neurologischen Erkrankungen (S. 19-22). Bad Honnef: Hippocampus Verlag.

Hoffmann, S. & Buld, S. (2006). Darstellung und Evaluation eines Trainings zum Fahren in der Fahrsimulation. In VDI-Gesellschaft Fahrzeug- und Verkehrstechnik (Hrsg.), Integrierte Sicherheit und Fahrerassistenzsysteme (VDI-Berichte, Nr. 1960, S. 113-132). Düsseldorf: VDI-Verlag.

Holmes, T.H. & Rahe, R.H. (1967). "The Social Readjustment Rating Scale". Journal of Psychosomatic Research 11 (2), 213–218.

Kaussner, Y. (2007). Fahrtauglichkeit bei M. Parkinson. Dissertation. Bayerische Julius-Maximilans-Universität Würzburg. Verfügbar unter http://opus.bibliothek.uni-wuerzburg.de/volltexte/2007/2250/

Kennedy, R.S., Lane, N. E., Berbaum, K.S., & Lilienthal, M.G. (1993). Simulator Sickness Questionnaire: An Enhanced Method for Quantifying Simulator Sickness. The International Journal of Aviation Psychology, 3, 203-220.

König, J. (2012). Posttraumatische Belastungsstörung: Ein Manual zur Cognitive Processing Therapy. Göttingen: Hogrefe.

Kuch, K., Cox, B. J. & Direnfeld, D. M. (1995). A brief self-rating scale for PTSD after road vehicle accident. Journal of Anxiety Disorders, 9, 503-514.

Maercker, A. (2003). Therapie der posttraumatischen Belastungsstörungen (2nd ed.). Berlin, Heidelberg: Springer-Verlag.

McNally, R. J. (2007). Mechanisms of exposure therapy: How neuroscience can improve psychological treatments for anxiety disorders. Clinical psychology review, 27, 750-9.

McLay, R. N., McBrien, C., Wiederhold, M. D., & Wiederhold, B. K. (2010). Exposure therapy with and without virtual reality to treat PTSD while in the combat theater: A parallel case series. Cyberpsychology, Behavior, and Social Networking, 13, 37-42.

Michaliszyn, D., Marchand, A., Bouchard, S., Martel, M.-O., & Poirier-Bisson, J. (2010). A randomized, controlled clinical trial of in virtuo and in vivo exposure for spider phobia. Cyberpsychology, Behavior, and Social Networking. 13, 689-695.

Mühlberger, A., Herrmann, M. J., Wiedemann, G., Ellgring, H. & Pauli, P. (2001). Repeated exposure of flight phobics to flights in virtual reality. Behaviour Research and Therapy, 39, 1033-1050.

Mühlberger, A., & Pauli, P. (2011). Virtuelle Realität in der Psychotherapie. PiD - Psychotherapie im Dialog, 2, 143-147.

Mühlberger, A., Wiedemann, G. & Pauli, P. (2003). Efficacy of a one-session virtual reality exposure treatment for fear of flying. Psychotherapy Research, 13, 323-336.

Neukum, A. & Krüger, H. P. (2003). Fahrerreaktionen bei Lenksystemstörungen - Untersuchungsmethodik und Bewertungskriterien. In: VDI-Gesellschaft Fahrzeug- und Verkehrstechnik (ed.). Reifen-Fahrwerk-Fahrbahn. VDI-Berichte, Nr. 1791. VDI-Verlag, Düsseldorf.

Nübling, R., Schmidt, J. & Schulz, H. (2002). FPTM Fragebogen zur Psychotherapiemotivation. In E. Brähler, J. Schuhmacher & B. Strauß (Hrsg.), Diagnostische Verfahren in der Psychotherapie. Göttingen: Hogrefe.

Nyberg, E., Stieglitz, R. D., Frommberger, U., & Berger, M. (2003). Psychological disorders after severe occupational accidents. Versicherungsmedizin, 55, 76-81.

Powers, M. B., & Emmelkamp, P. M. G. (2008). Virtual reality exposure therapy for anxiety disorders: A meta-analysis. Journal of Anxiety Disorders, 22(3), 561-569.

Rothbaum, B. O., Meadows, E. A., Resick, P., & Foy, D. W. (2000). Cognitive-behavioral therapy. In Foa, E. B., Keane, T. M., Friedman, M. J. (Eds.), Effective treatments for PTSD: Practice guidelines from the International Society for Traumatic Stress Studies (pp. 320-325). New York, NY: Guilford Press.

Rothbaum, B. O. (2009). Using virtual reality to help our patients in the real world.Depression and Anxiety, 26(3), 209-211.

Schaarschmidt, U. & Fischer, A. (2003). AVEM - Arbeitsbezogenes Verhaltens- und Erlebensmuster. Handanweisung. Zweite überarbeitete und erweiterte Auflage. Frankfurt: Swets & Zeitlinger.

Schwarzer, G., & Schumacher, M. (2007). Die Beurteilung der Gleichwertigkeit von Behandlungen. In M. Schumacher & G. Schulgen (Eds.), Methodik klinischer Studien. Methodische Grundlagen der Planung, Durchführung und Auswertung (2. Aufl. ed., pp. 113-127). Berlin: Springer.

Statistisches Bundesamt (2014). Verkehrsunfälle –Fachserie 8 Reihe 7 – Oktober 2013. Wiesbaden. Verfügbar unter: https://www.destatis.de/DE/Publikationen/Thematisch/TransportVerkehr/Verkehrsunfaelle/VerkehrsunfaelleMonat/VerkehrsunfaelleM2080700131104.pdf?__blob=publicationFile

Traue, H.C., Hrabal, V. & Kosarz, P. (2000). AlltagsBelastungsFragebogen (ABF): Zur inneren Konsistenz, Validierung und Stressdiagnostik mit dem deutschsprachigen Daily Stress Inventory. Verhaltenstherapie und Verhaltensmedizin, 21, 15-38.

Wald, J. (2004). Efficacy of virtual reality exposure therapy for driving phobia: A multiple baseline across-subjects design. Behavior Therapy, 35, 621-635.

Wald, J., & Taylor, S. (2000). Efficacy of virtual reality exposure therapy to treat driving phobia: A case report. Journal of Behavior Therapy and Experimental Psychiatry, 31, 249-257.

Wald, J., & Taylor, S. (2003). Preliminary research on the efficacy of virtual reality exposure therapy to treat driving phobia. CyberPsychology & Behavior, 6, 459-465.

Walshe, D., Lewis, E., O’Sullivan, K., & Kim, S. I. (2005). Virtually Driving: Are the driving environments “real enough” for exposure therapy with accident victims? An explorative study. CyberPsychology & Behavior, 8, 532-537.

Zöllner, T., Karl, A., Märcker, A., Hickling, E. J. & Blanchard, B. (2005) Manual zur Kognitiven Verhaltenstherapie von Posttraumatischen Belastungsstörungen bei Verkehrsunfallopfern.
